# Periodic polar vortices in BaTiO_3_/SrTiO_3_ superlattices monolithically grown on silicon

**DOI:** 10.1038/s41467-026-77270-0

**Published:** 2026-09-08

**Authors:** Valentin Väinö Hevelke, Ines Häusler, Aiden Ross, Israel Ibukun Olaniyan, Sven Wiesner, Minh Anh Luong, Leifeng Zhang, Sylvie Schamm-Chardon, Kristiane Elsner, Benedikt Haas, Adnan Hammud, Florian Bertram, Christoph T. Koch, Long-Qing Chen, Dong-Jik Kim, Catherine Dubourdieu

**Affiliations:** 1 Helmholtz-Zentrum Berlin für Materialien und Energie GmbH, Hahn-Meitner Platz 1, 14109 Berlin, Germany; 2 Freie Universität Berlin, Physical and Theoretical Chemistry, Arnimallee 22, 14195 Berlin, Germany; 3 Humboldt-Universität zu Berlin, Department of Physics, Newtonstraße 15, 12489 Berlin, Germany; 4 Department of Materials Science and Engineering and Materials Research Institute, The Pennsylvania State University, University Park, PA 16802 USA; 5 CEMES-CNRS and Université de Toulouse, 29 rue Jeanne Marvig, 31055 Toulouse, France; 6 Department of Inorganic Chemistry, Fritz Haber Institute of the Max Planck Society, 14195 Berlin, Germany; 7 Deutsches Elektronen-Synchrotron (DESY), 22607 Hamburg, Germany

**Keywords:** Structural properties, Ferroelectrics and multiferroics

## Abstract

Polar textures in ferroelectric superlattices have been so far reported almost exclusively in PbTiO_3_-based systems and predominantly on oxide substrates. Owing to their nanometer-scale dimensions and emergent functionalities, whirling topological polar textures would be especially compelling when realized on silicon with Complementary-Metal-Oxide-Semiconductor-compatible materials. Here, we demonstrate the stabilization of vortex tubes in BaTiO_3_/SrTiO_3_ superlattices epitaxially grown on silicon. The vortices in each BaTiO_3_ layer order along the <110> _BaTiO3_ crystallographic directions, forming an in-plane 90-degree stripe pattern. This polar configuration represents the ground state of the system as shown by phase field modelling and further validated by temperature dependent X-ray diffraction. Phase field modelling shows a strong dependence of the domain morphology on the strain and highlights the elastic and electrostatic coupling of the BaTiO_3_ and SrTiO_3_ layers. Our findings expand the materials and substrate platforms known to host polar topological textures, opening new opportunities for integrating complex ferroelectric states with silicon-based technologies.

## Abstract (web summary)

The authors present the stabilization of polar vortex tubes in epitaxial BaTiO_3_/SrTiO_3_ superlattices monolithically grown on silicon, and their temperature behavior. Phase field simulations give insight into the strain-dependent evolution of the polar domains.

## Introduction

The discovery of topological polar textures in nanoscale ferroelectrics has sparked tremendous interest due to their fascinating physics and potential for next-generation high-density electronic devices. Over the last fifteen years, the experimental observations of continuous dipole rotations^1,2^, periodic flux closures^3,4^, vortices^5–11^, center-type domains^12,13^, bubbles^14,15^, skyrmions^16,17^, vortex-antivortex pairs^18–20^, polar waves^21,22^, supercrystals^23–25^, merons^17,26^ and polar Bloch-points^27^ have been reported. These rotational polarization textures have nanoscale dimensions (typically below 10 nm for the diameter of polar skyrmion bubbles or vortex tubes) and may exhibit emergent properties such as negative capacitance^28,29^ and chirality^30,31^. Hence, polar topologies offer opportunities for ultra-high-density memory devices and for novel functionalities in low-power electronics, or photonics, broadly captured under the term topotronics^32^. Well-known examples in this context are magnetic racetrack memories based on magnetic skyrmions^33,34^. Analogue to this, recent theoretical predictions of high electric-field-driven mobilities (25 m/s) of polar skyrmion bubbles^35^ suggest the possibility of realizing electric racetrack memory devices. Electrically driven architectures could offer improved energy efficiency compared to their magnetic counterparts. In addition, the highly tunable dielectric response and negative capacitance behavior of polar textures under applied electric fields could be exploited in low-power and reconfigurable electronic components, such as adaptive capacitors or steep-slope transistors^29,36^. Recent progress in the manipulation of polar textures using external stimuli, such as electric fields and light, reveals exciting opportunities for creating novel device functionalities. For instance, the erasure of the toroidal order of polar vortices^21,37^ and the switching of vortex tube chirality^31^ demonstrate the possible manipulation of these polar structures. Furthermore, the recent discovery of ultra-fast electric field responses from polar vortices in the sub-THz regime highlights their potential for high-speed data processing and storage applications^38^. Also, the interconversion of different polar topologies through light and temperature show exciting pathways to engineering polar architectures^17,24,39–42^.

The formation of the topological polarization textures depends on a delicate balance between electrostatic boundary conditions, mechanical boundary conditions (epitaxial strain), and polar gradients^43^. Most of the studies on polar textures have focused on PbTiO_3_ or Pb(Zr,Ti)O_3_/SrTiO_3_ bilayers, trilayers or superlattices grown on SrTiO_3_ or scandate substrates. In these prototypical model systems, boundary conditions can be precisely controlled by tuning the thickness and the period of the constituent layers^9,44^ and by selecting appropriate substrates^4,8^. BiFeO_3_-based heterostructures have also been investigated, such as GdScO_3_/BiFeO_3_/GdScO_3_ on TbScO_3_ substrates^45^ and BiFeO_3_/SrTiO_3_ superlattices on LaAlO_3_ substrates^7^.

For BaTiO_3_/SrTiO_3_ superlattices, there is only one report, to the best of our knowledge, of curled polarization on SrTiO_3_ substrates^46^. In this study, no periodic pattern and no particular polar textures were reported, but rather irregular domains of localized curled polarization. This might suggest that the conditions imparted by the substrate are not favorable for the stabilization of a periodic polar pattern. Overall, there are very scarce realization of polar textures in BaTiO_3_-based materials. Free standing BaTiO_3_ nanoparticles^47,48^, free standing BaTiO_3_ lamellae^49^ and twisted BaTiO_3_ membranes^10^ have been shown to host vortex-like polar textures. Recently, chiral center-type polar nanodomains have been realized in trapezoidal-shaped nano islands (30-60 nm) grown on silicon^50^, and topological states have been reported in 100 nm-diameter BaTiO_3_ nanodisks^51^, suggesting the feasibility of realizing complex polarization states on Si without the use of lead-based materials.

Bringing the functionality of polar topologies into the world of Complementary Metal Oxide Semiconductor (CMOS) nanoelectronics has hardly been addressed. On silicon substrates, the only realization, to the best of our knowledge, concerns bilayers grown on a sacrificial Sr_3_Al_2_O_6_ layer on a SrTiO_3_ substrate and then transferred onto silicon, where the authors report skyrmion-like polar nanodomains^52^. Such route faces issues of mechanical stability and strain-induced curling^53^. The monolithic integration of polar textures on Si would facilitate their robust integration in nanoelectronics. For this purpose, BaTiO_3_ is a suitable candidate, whereas Pb-based compounds pose environmental and health risks, and Bi-based compounds are undesirable in an industrial cleanroom due to high Bi volatility. The monolithic growth of epitaxial BaTiO_3_ on silicon by molecular beam epitaxy using a SrTiO_3_-buffer layer on Si^54–60^ is well established. Epitaxial superlattices of BaTiO_3_/SrTiO_3_ on Si have been synthesized, however, with no report of polar textures^61–63^.

In this work, we report the realization of ordered clockwise / counterclockwise vortices in (4.8 nm BaTiO_3_/ 2.0 nm SrTiO_3_)_10_ superlattices grown on silicon. Synchrotron X-ray diffraction reciprocal space maps indicate that the periodicity of the polar pattern is along the <110> directions of BaTiO_3_ and SrTiO_3_, in contrast to the <100> ones typically observed in PbTiO_3_-SrTiO_3_ superlattices. The polar pattern ordering disappears at about 450 K, which corresponds to the Curie temperature of the tetragonal to cubic phase transition of the BaTiO_3_ ultrathin films, as shown by Raman spectroscopy. The polar whirling patterns are evidenced at the nanoscale by the Ti-displacement maps determined from aberration-corrected high-angle annular dark-field (HAADF) scanning transmission electron microscopy (STEM). Phase field modeling closely matches the experimental results and shows that the ordering of the polar pattern is highly sensitive to changes in epitaxial strain. With these results, we show that periodic polar patterns can be established in BaTiO_3_ superlattices, moreover, on silicon, which will allow further development of CMOS-compatible topotronic devices on silicon.

## Results and discussion

### Structural characterization

**Fig. 1:**
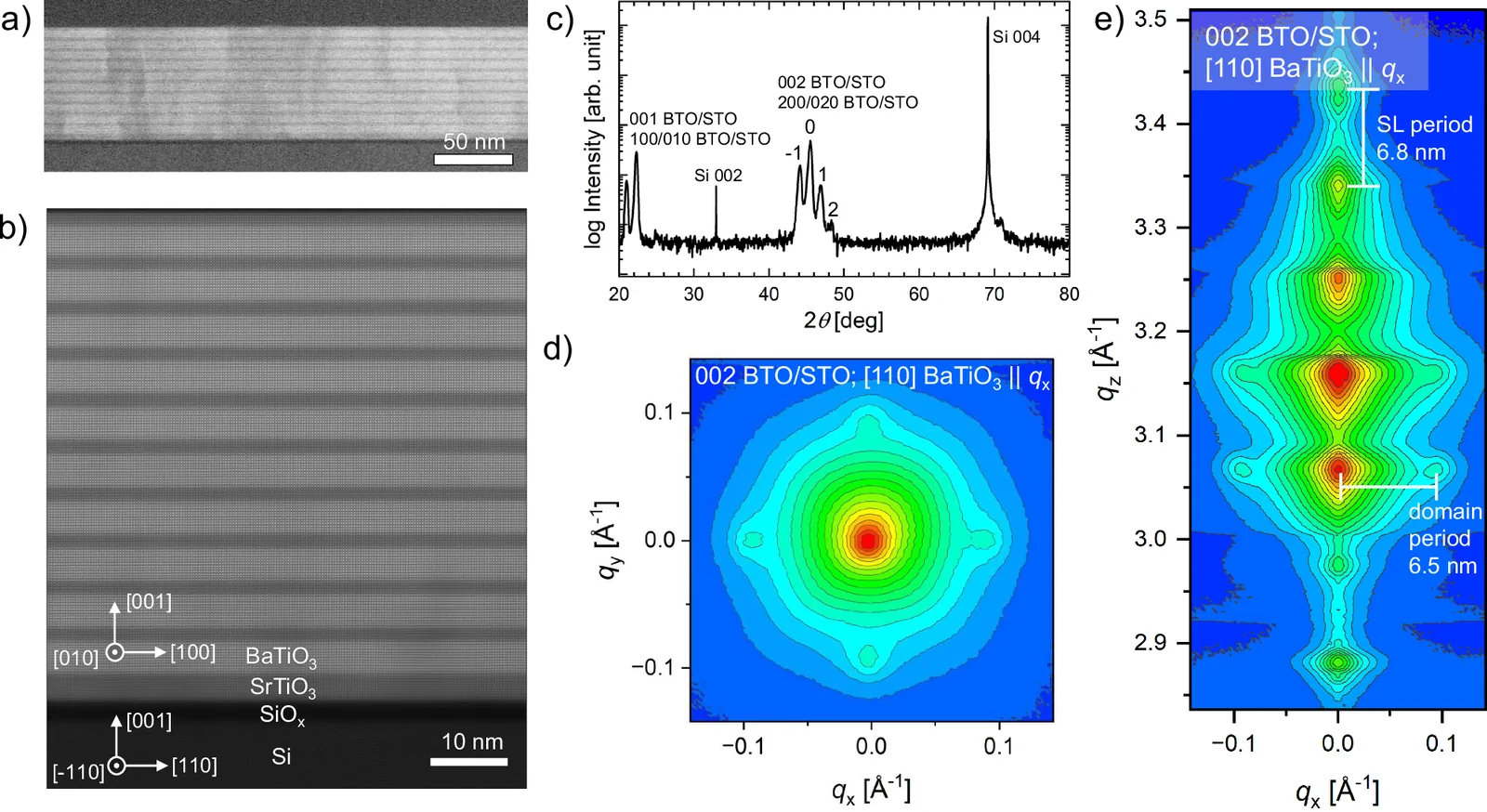
Structural characterization of a (12 u.c. BaTiO_3_ / 5 u.c. SrTiO_3_)_10_ superlattice on silicon Si(001). **a** Low-magnification HAADF-STEM image of the superlattice. Dislocation defects are seen as lateral contrast variations extending vertically. **b** High-resolution HAADF-STEM image of the superlattice. A thin SiO_x_ layer at the interface between the 4 nm SrTiO_3_ buffer layer and the Si substrate is formed during the growth of the superlattice. **c** $\theta /2\theta$ X-ray diffraction scan showing the 00l Bragg-peaks of the Si substrate and of the superlattice. **d**, **e** Synchrotron-based reciprocal space maps around the 002 Bragg peak **d**) in the *q*_x_^_^*q*_y_ plane and **e**) in the *q*_x_^_^*q*_z_ plane. For these measurements (**d**, **e**), the samples were oriented with the [110] crystallographic axis of BaTiO_3_/SrTiO_3_ parallel to the X-ray beam (Fig. S6.d of the Supporting Information).

The epitaxial BaTiO_3_/SrTiO_3_ superlattices were grown on SrTiO_3_-buffered Si (001) by molecular beam epitaxy. We focus here on superlattices with ten repetitions of 12 unit-cells (u.c.) BaTiO_3_ (~4.8 nm) and 5 u.c. SrTiO_3_ (~2.0 nm). Details of the growth are given in the Method Section. The cross-sectional high-angle annular dark field scanning transmission electron microscopy (HAADF-STEM) images in Fig. 1a, b show the superlattice structure with alternating layers of BaTiO_3_ and SrTiO_3_, in which the BaTiO_3_ layers appear brighter than SrTiO_3_ layers. The contrast throughout the thickness in Fig. 1a indicates the presence of dislocations, typical for superlattices grown on silicon due to the large difference in thermal expansion coefficients between silicon and perovskite oxides. Sharp interfaces are observed between the ultrathin BaTiO_3_ and SrTiO_3_ layers (Fig. 1.b). However, atomically resolved chemical maps obtained by energy-dispersive X-ray spectroscopy (EDX) reveal A-site cation (Ba, Sr) intermixing at each interface over one unit cell (Fig. S1 of the Supporting Information). The intermixing is favored by a non-perfect 2D growth mode and MBE co-deposition growth (Ba and Ti for BaTiO_3_, Sr and Ti for SrTiO_3_), and therefore, the absence of monolayer-precise growth termination. X-ray diffraction and RHEED images confirm the epitaxial nature of the superlattice with the following epitaxial relationship: BaTiO_3_/SrTiO_3_ (001) || Si (001) and BaTiO_3_/SrTiO_3_ [100] || Si [110] (Fig. 1.c and Figs. S2–S4 of the Supporting Information). The average out-of-plane and in-plane lattice parameters of the superlattice are 3.992 Å and 3.982 Å, respectively. Raman measurements (Fig. S5.a of the Supporting Information) indicate the coexistence of *c*- and *a*-oriented unit cells of BaTiO_3_ (i.e., the long axis of the tetragonal cell is perpendicular and parallel to the substrate plane, respectively). The A(TO_3_) mode at 519 cm^−1^ indicates the presence of *a*-axis orientation, while the A(LO_2_) + E(LO_3_) and A(LO_3_) + E(LO_4_) modes at 475 cm^−1^ and 725 cm^−1^, respectively, indicate the presence of *c*-axis orientation^64^. Reference spectra measured on *c*-axis and *a*-axis BaTiO_3_ single crystals are shown in Fig. S5.b of the Supporting Information. Synchrotron reciprocal space maps (RSMs) were measured with the direction of *q*_x_ aligned along the [110] direction (Fig. 1.d, e) and [100] direction (Fig. S6 of the Supporting Information) of BaTiO_3_, respectively. Figure 1.c, e show satellite peaks along the out-of-plane direction, indicating a superlattice periodicity of 6.8 nm, which corresponds indeed to the 4.8 nm BaTiO_3_ and 2.0 nm SrTiO_3_. Each superlattice reflection is accompanied by in-plane satellite peaks indicating a lateral long-range ordering of ferroelectric domains along the <110> _BaTiO3_ directions with a period of ~6.5 nm (Fig. 1.e and Fig. S6.f of the Supporting Information). The lateral ordering is also clearly observed on the *q*_x_-*q*_y_ reciprocal space map (Fig. 1.d). Note that the polar textures reported in (PbTiO_3_/SrTiO_3_) superlattices order along <100> _PbTiO3_ directions, while here it is along the <110> _BaTiO3_ ones. We will discuss this point later.

The study of two other superlattices, (12 u.c. BaTiO_3_/*n* u.c. SrTiO_3_)_10_ with *n* = 7 u.c. (~2.8 nm) and 10 u.c. (~4.0 nm), shows the presence of similar lateral satellites corresponding to periodicities of 6.7 ± 0.1 nm and 6.5 ± 0.1 nm, respectively (Fig. S7 of the Supporting Information). There is no significant change in the periodicity or density of the polar vortices in the investigated range of SrTiO_3_ thicknesses (2.0–4.0 nm).

### Local study of the periodic polar textures

**Fig. 2:**
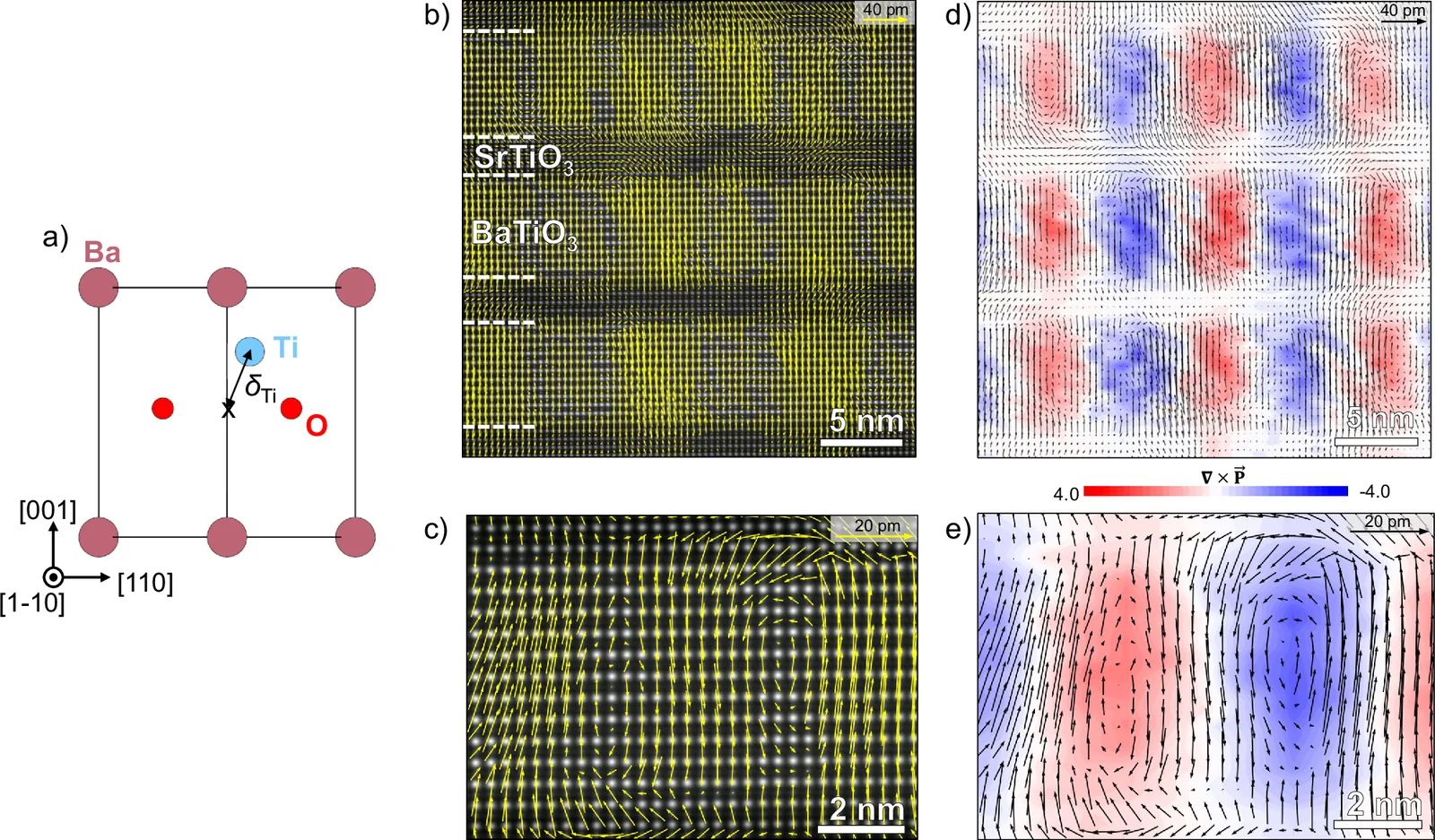
Study of the periodic polar vortices in a (12 u.c. BaTiO_3_ / 5 u.c. SrTiO_3_)_10_ superlattice on silicon Si(001) from STEM Ti-displacement maps. **a** Projection of the tetragonal unit cell of BaTiO_3_ along the [110] direction. The displacement of the Ti ion from the center of the unit cell determines the polarization direction and magnitude. **b**, **c** HAADF-STEM images obtained on a [110]_BaTiO3_ cut lamellae of the superlattice (with zone axis of [100] relative to the Si substrate) overlaid with the corresponding Ti-displacement map. The small yellow arrows indicate the direction of the Ti-displacement. The length of the arrows scales with the magnitude of the Ti-displacement. **d**, **e** Ti displacement map underlaid with the curl of the Ti-displacement vector field. Clockwise and counterclockwise rotation is indicated by red and blue colors, respectively.

The displacement of the Ti ions relative to the barycenter of the Ba (Sr) atoms in each unit cell (Fig. 2.a) was determined from cross-sectional atomic-resolution HAADF-STEM images. The resulting displacement maps obtained on [110]_BaTiO3_ cut lamellae show periodic arrays of clockwise and counterclockwise polar vortices (Fig. 2.b–e). We determined an average vortex periodicity of ∼6.9 nm from several STEM images recorded on different locations of the sample, which is close to the ﻿∼6.5 nm periodicity determined by RSM (there are slight inhomogeneities in the superlattice as indicated by the dispersion of the observed periodicity ranging from 6.4 nm to 7.5 nm depending on the location on the sample). Due to the strong and highly inhomogeneous strain fields around dislocations, the atomic positions and, therefore, the Ti-displacement vectors can not be precisely determined in their vicinity. Hence, we cannot determine whether the dislocations affect the periodicity of the polar patterns.

The Ti-displacement maps obtained on a [100]_BaTiO3_ cut lamellae are shown in Fig. S8 of the Supporting Information. It shows vortices but not as well defined and, in some locations, a polar wave in the BaTiO_3_ layers. The stability of polar waves versus polar vortices is largely governed by the competition between electrostatic and gradient energy^22,41^. The electrostatic energy promotes the stability of vortices over the polar waves as they minimize the polar discontinuity at the BaTiO_3_/SrTiO_3_ interface, while the gradient energy penalty destabilizes the polar vortices and promotes the dipolar wave since the gradient of the polarization is more gradual in the dipolar wave structure. As the magnitude of the spontaneous polarization decreases, the electrostatic energy penalty decreases, thereby stabilizing the polar wave, as evidenced by the observation that the polar wave is stabilized at higher temperatures^41^. In addition to electrostatic considerations, local strain inhomogeneities may play an important role in the appearance of polar vortices or polar waves. Phase diagrams obtained from phase-field simulations for the PbTiO_3_/SrTiO_3_ superlattices show that polar waves are stabilized under higher tensile strain and that under certain strain conditions, both polar waves and vortices appear^22^. In our superlattice, dislocation defects introduce strongly inhomogeneous strain fields that locally modify the elastic energy landscape. Such strain gradients could locally favor specific polarization orientations.

SrTiO_3_ in the superlattice is polarized at the interfaces with BaTiO_3_ layer and shows in-plane and out-of-plane components (Fig. 2 and Figs. S8 and S9 of the Supporting Information). The direction of Ti-displacement in the SrTiO_3_ layers follows the one in the adjacent BaTiO_3_ layers. The SrTiO_3_ unit cells are more polarized at the interfaces to the adjacent BaTiO_3_ layers than inside the layers. SrTiO_3_ is paraelectric in its bulk form and known as an incipient ferroelectric^65^. Thin films under tensile strain have been reported to be ferroelectric^66^. Similarly, highly polarized ultrathin SrTiO_3_ layers in PbTiO_3_/SrTiO_3_/PbTiO_3_ heterostructures were reported^44,67,68^. This polarization, in turn, mediates coupling between the BaTiO_3_ layers, creating an ‘in-phase’ ordering of the vortices in the growth direction (Fig. 2.b, d). These points will be discussed later with the phase field modelling results.

**Fig. 3:**
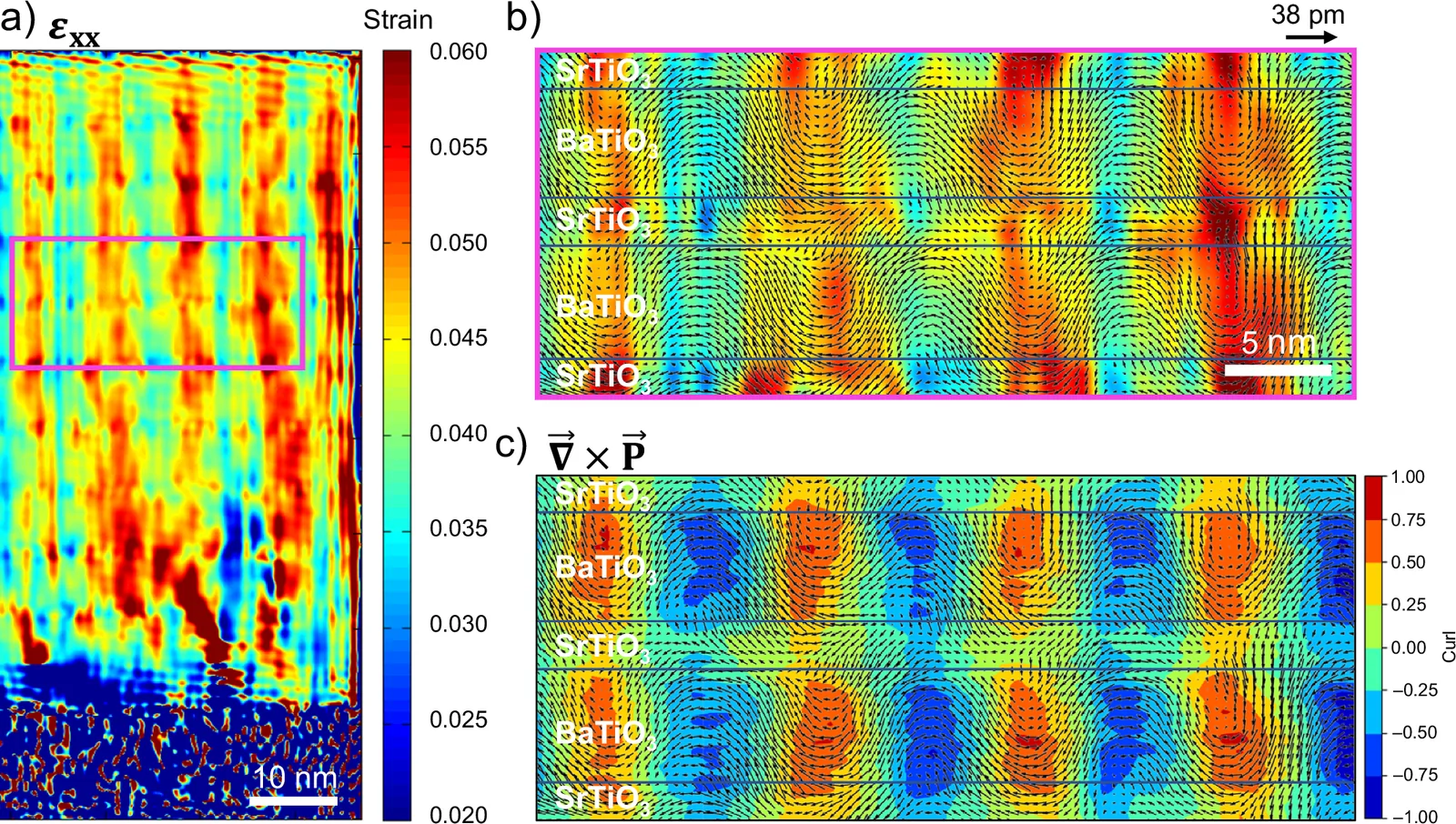
Geometric phase analysis for the strain analysis of a (12 u.c. BaTiO_3_ / 5 u.c. SrTiO_3_)_10_ superlattice on silicon Si(001) (silicon is taken as the reference for the strain). **a** Map of the in-plane strain *ε*_xx_. **b** Zoom of the region in the pink box in (**a**) overlaid with the Ti-displacement vector map. **c** Ti-displacement vector map superimposed to the map of the curl (rotational) of the Ti displacement vector.

The map of the in-plane strain *ε*_xx_ (Fig. 3.a, b) determined by geometric phase analysis (GPA) shows a periodic lateral modulation of the strain in the superlattice. There is a strong correlation/coupling between the strain pattern and the rotation of the polarization: regions characterized by negative and positive curls, as shown on the map of the rotation of the polarization ($\vec{\nabla }\times \vec{P}$) in Fig. 3.c correspond to areas of low and high in-plane strain, respectively (Fig. 3.b).

### In-plane ordering

**Fig. 4:**
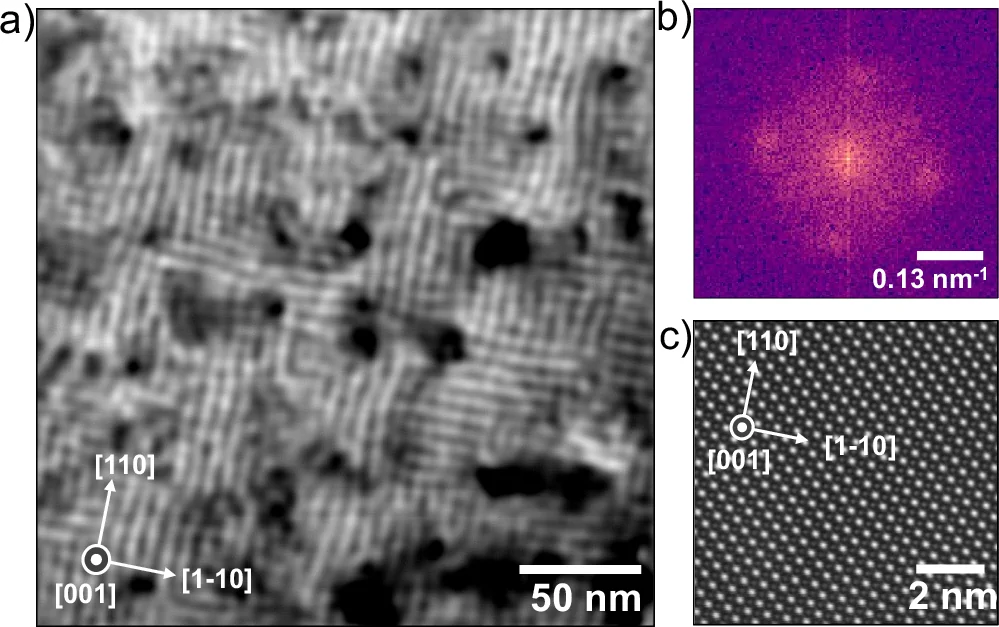
STEM planar view of a (12 u.c. BaTiO_3_ / 5 u.c. SrTiO_3_)_10_ superlattice on silicon Si(001). **a** Low magnification planar-view HAADF-STEM image showing a stripe pattern arising from different polarization orientations. **b** Fast Fourier transform of the image in (**a**) showing periodicity along <110> directions of BaTiO_3_. **c** High resolution HAADF-STEM image taken from a region in (**a**) showing the crystal orientation.

HAADF-STEM plane view images (Fig. 4.a) show a 90° stripe pattern. This observation indicates that the vortices observed in cross-section images extend as vortex tubes along the third dimension and that they are indeed ordered along the <110> directions of BaTiO_3_ (Fig. 4.a–c). The periodic vortex tubes are disrupted by dislocation defects which appear as regions of dark contrast in Fig. 4.a. The periodic stripe pattern terminates or locally loses coherence in the regions where dislocations are present, indicating that these defects disturb the long-range lateral ordering of the polar vortices. The average periodicity along <110> _BaTiO3_ is of ~7.0 nm as determined by Fast Fourier transform (Fig. 4.b), quite close to the value determined from cross-view lamellae (~6.9 nm).

Note that we have attempted to image the polar vortices using piezo-response force microscopy (PFM) but could not resolve the polar pattern (the size of individual vortices is of ~3.5 nm) due to the tip-limited resolution (the diameter of the tip is of ~20 nm).

### Polar topologies under temperature cycling

**Fig. 5:**
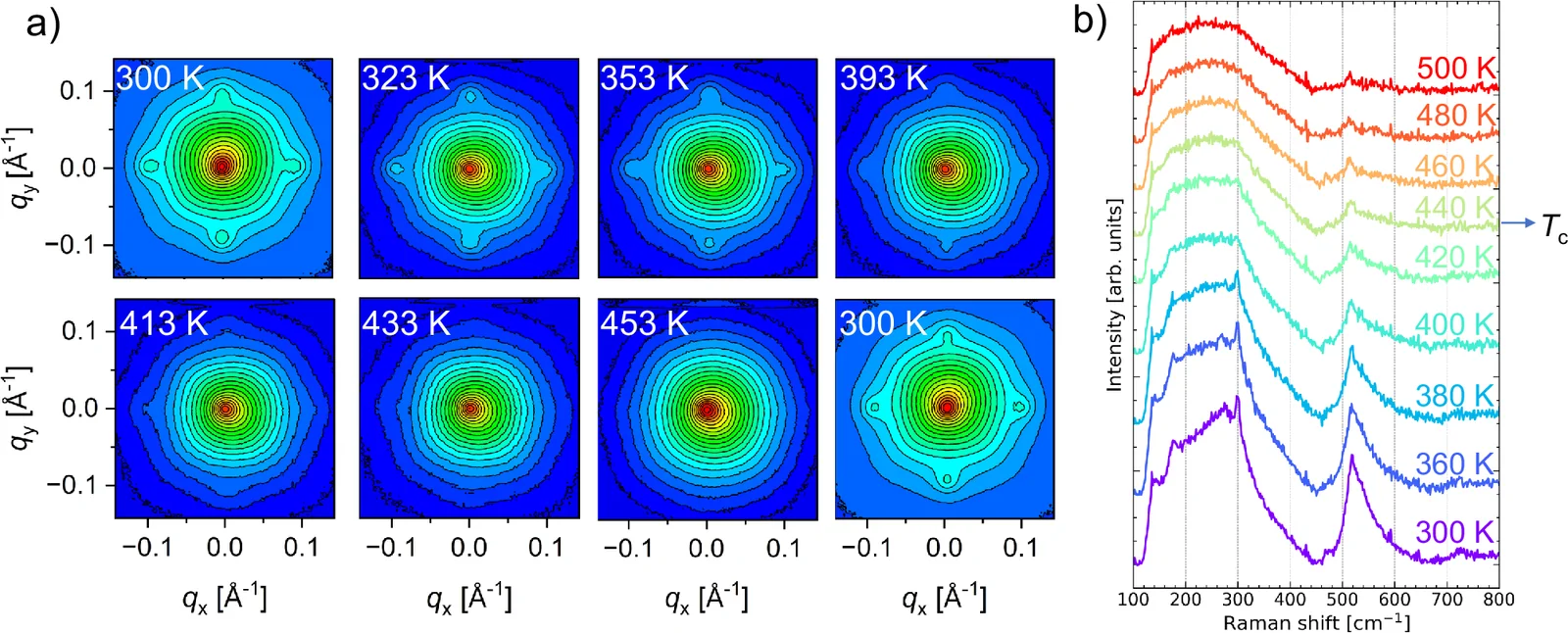
Temperature dependence of the long-range order of polar vortices in a (12 u.c. BaTiO_3_ / 5 u.c. SrTiO_3_)_10_ superlattice on silicon Si(001). **a** Synchrotron-based reciprocal space maps recorded at different temperatures. The corresponding line profiles at *q*_y_ = 0 along *q*_x_ are presented in Fig. S10 of the Supporting Information. **b** Temperature-dependent Raman spectra showing the disappearance of the Raman modes indicative of tetragonal BaTiO_3_ at a temperature of ∼440 K. The indexed Raman spectrum recorded at 300 K is presented in Fig. S5a of the Supporting Information.

Synchrotron RSM measurements (Fig. 5.a) were performed at different temperatures from 300 up to 453 K. As the temperature increases, the intensity of the satellite peaks indicative of lateral periodic ordering gradually diminishes and almost disappears at 453 K. The periodicity of the polar vortices gradually decreases upon heating, from 6.5 nm at 300 K down to 5.5 nm at 433 K (Fig. S10 of the Supporting Information). Upon cooling the superlattice back to room temperature, the satellite peaks reappear at their original positions and intensity. These observations suggest that the vortex pattern ordered along <110 > _BaTiO3_ is the ground state of the BaTiO_3_/SrTiO_3_ superlattice on Si. As we do not have intermediate data upon cooling down for the vortex periodicity, we can not conclude whether the vortex formation is hysteretic or not with temperature.

Raman spectroscopy and XRD measurements on the same sample in the temperature range 300–500 K (Fig. 5.b) and 300–673 K (Fig. S11), respectively, reveal a structural phase transition in BaTiO_3_. The intensity of the B_1_ + E(LO + TO) at ∼300 cm^−1^, indicative of the BaTiO_3_ tetragonality^69^, vanishes at 440 K, which marks the ferroelectric-to-paraelectric phase transition, as shown by temperature-dependent Raman measurements performed on a *c*-oriented BaTiO_3_ single crystal (Fig. S5.c of the Supporting information). In XRD, a change in slope for the *c*-axis lattice parameter as a function of temperature (Fig. S11) appears at 449 K (176 °C), in very good agreement with the Raman spectroscopy results, considering the different set-up/temperature measurements. The *T*_c_ of the superlattice is therefore in the range 440–450 K. Note that there is no hysteretic behavior of the lattice parameters upon heating/cooling.

The *T*_c_ of the superlattice is lower than that of 20 nm-thick BaTiO_3_ films on SrTiO_3_-buffered Si (*T*_c_ ~ 543 K) grown in identical conditions^51^, but larger than the value for bulk BaTiO_3_ (*T*_c_ = 393–403 K^70^). It is well known that the *T*_c_ of ferroelectric thin films is reduced when the thickness is decreased due to the depolarization field. However, the strain state also has a determining role in *T*_c_. Ultrathin (below 5 nm) epitaxial BaTiO_3_ films have been shown to have *T*_c_ exceeding the bulk’s *T*_c_. For a 4.0 nm BaTiO_3_ film fully strained to SrTiO_3_, Tenne et al. reported a *T*_c_ (experimental) of ~525 K if the film was uncapped and of ~560 K if the film was capped with a 10 nm SrTiO_3_ film^71^. If we extrapolate their results, the *T*_c_ for a 4.8 nm BaTiO_3_ film fully strained to SrTiO_3_ is ~740 K. For superlattices like (8 u.c. BaTiO_3_ / 4 u.c. SrTiO_3_)_10_ grown on SrTiO_3,_ a *T*_c_ value of ~640 K was reported and the extrapolation to (12 u.c. BaTiO_3_ / 4 u.c. SrTiO_3_)_10_ gives a *T*_c_ much larger than 700 K^69^. Hence, despite the depolarization field, the Curie temperature in ultrathin BaTiO_3_ films (< 5 nm) fully strained to SrTiO_3_ in superlattices is significantly larger than that in bulk BaTiO_3_. In our case, since BaTiO_3_ is not fully strained to SrTiO_3_ but partially relaxed^51^, *T*_c_ is smaller but still significantly larger than the bulk value.

From the Raman spectroscopy and X-ray diffraction measurements, we deduce that the periodicity of polar vortices observed at room temperature along <110>_BaTiO3_ decreases upon heating and gradually disappears as the Curie temperature is approached. The gradual decrease of the intensity of the satellite peak indicates a second-order rather than a first-order phase transition.

### Phase-field simulations

**Fig. 6:**
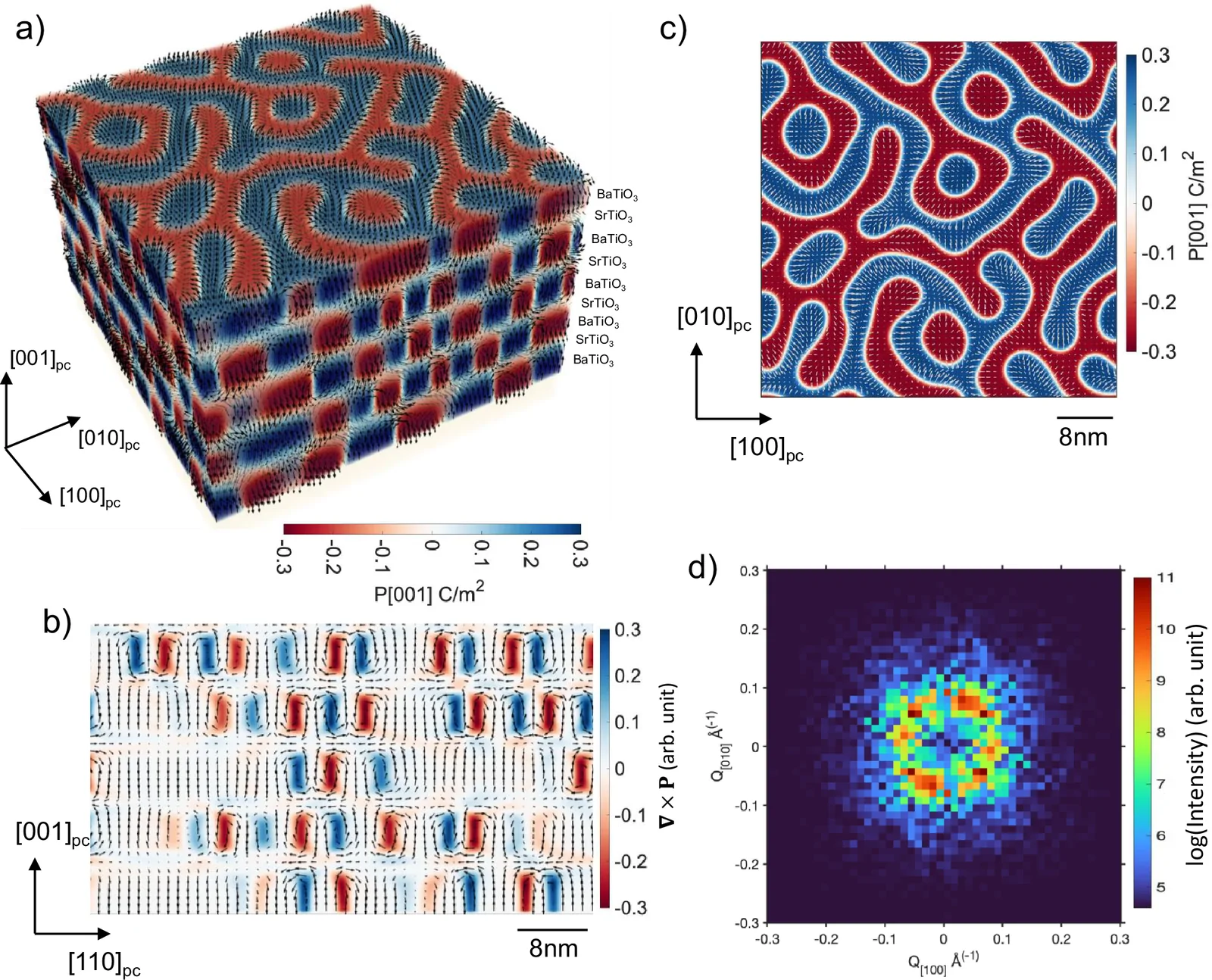
Phase-field simulations of a (12 u.c. BaTiO_3_ / 5 u.c. SrTiO_3_)_10_ superlattice on silicon Si(001). **a** Three-dimensional rendering of the polarization distribution. **b** Two-dimensional slice of the polarization projected onto the (110) plane showing the curl of the polarization. **c** Slice projected onto the (001) plane colored by the polarization along the [001] direction. **d** Fast Fourier transform (FFT) of the (001) polarization slice showing distinct peaks corresponding to preferential ordering along the <110> directions. All calculations were done using an effective lattice parameter of 3.99 Å.

Phase-field simulations were performed to further investigate the mesoscale ordering of the polar textures. Figure 6.a presents a three-dimensional rendering of the polarization distribution, with the black arrows representing the polarization magnitude and direction and the color denoting the polarization along the out-of-plane direction ([001]_pc_). The simulations reveal a labyrinthian structure with a preferential alignment of the vortex tubes along the <110> directions, consistent with the STEM and RSM observations. Figure 6.b shows a two-dimensional slice along the (110) plane, with the color denoting the curl of the polarization ($\vec{\nabla }\times \vec{P}$) normal to the surface. In some areas, the simulations show an ordered array of vortices where adjacent vortices exhibit opposite curl directions forming a checkerboard-like pattern, where in other areas, there is no curl normal to the surface due to the vortex tube running parallel to the (110) plane. Indeed, the appearance of discrete polarization states, rather than a continuous vortex texture, arises from the fact that the polar vortices are ordered along two orthogonal directions. In each 2D slice, the cutting plane intersects some vortex cores perpendicular to their axis while remaining parallel to others. As a result, vortices whose cores lie parallel to the slicing plane do not appear as circulating polarization patterns in that projection, leading to regions that exhibit apparently uniform polarization rather than vortex-like features.

A corresponding slice along the (001) plane is shown in Fig. 6.c, highlighting the in-plane labyrinthian ordering. The simulations indicate a moderate preference for the vortex tubes to align along the <110> family of directions, with isolated skyrmions scattered across the domain structure. To further investigate the spatial ordering, Fig. 6.d presents the Fast Fourier Transform (FFT) of the (001) plane, which exhibits distinct intensity peaks along the <110> directions, with a periodicity of 7.24 nm, which closely matches the in-plane periodicity found in experiment (7.0 nm in plane-view STEM).

A quantitative comparison of the key parameters between the experiment and phase field simulations is presented in Table S1 of the Supporting Information.

Phase-field simulations indicate that the termination of polar vortex tubes can occur through the formation of polar Néel-type disclinations (Fig. S12 of the Supporting Information).

To further investigate the influence of strain on the ordering of the polarization textures, we performed a series of simulations under different strain conditions. Since the BaTiO_3_ and SrTiO_3_ are elastically coherent, the average in-plane lattice parameter in each layer must match. Although their lattice parameters must match, they possess different reference lattice parameters (${a}_{{BaTiO}3}^{{eq},\,{cubic}}=4.015{{\mathrm{\AA}}},\,{a}_{{SrTiO}3}^{{eq},\,{cubic}}=3.905\,{{\mathrm{\AA}}}$) which gives rise to different strains in each layer. For instance, if the average in-plane lattice parameter is between 3.905 Å and 4.015 Å, then SrTiO_3_ will experience a tensile strain, while BaTiO_3_ will experience a compressive strain. Here we shall define an effective substrate lattice parameter that denotes the average in-plane lattice parameter across the entire superlattice, and using it we may calculate the in-plane strain in both the BaTiO_3_ and SrTiO_3_ layers:

1 $${\varepsilon }_{11}^{{BaTi}{O}_{3}}={\varepsilon }_{22}^{{BaTi}{O}_{3}}=\frac{{{a}^{{eff}}-a}_{{BaTiO}3}^{{eq},{cubic}}}{{a}_{{BaTiO}3}^{{eq},{cubic}}},{\varepsilon }_{11}^{{SrTi}{O}_{3}}={\varepsilon }_{22}^{{SrTi}{O}_{3}}=\frac{{{a}^{{eff}}-a}_{{SrTiO}3}^{{eq},{cubic}}}{{a}_{{SrTiO}3}^{{eq},{cubic}}}.$$

**Fig. 7:**
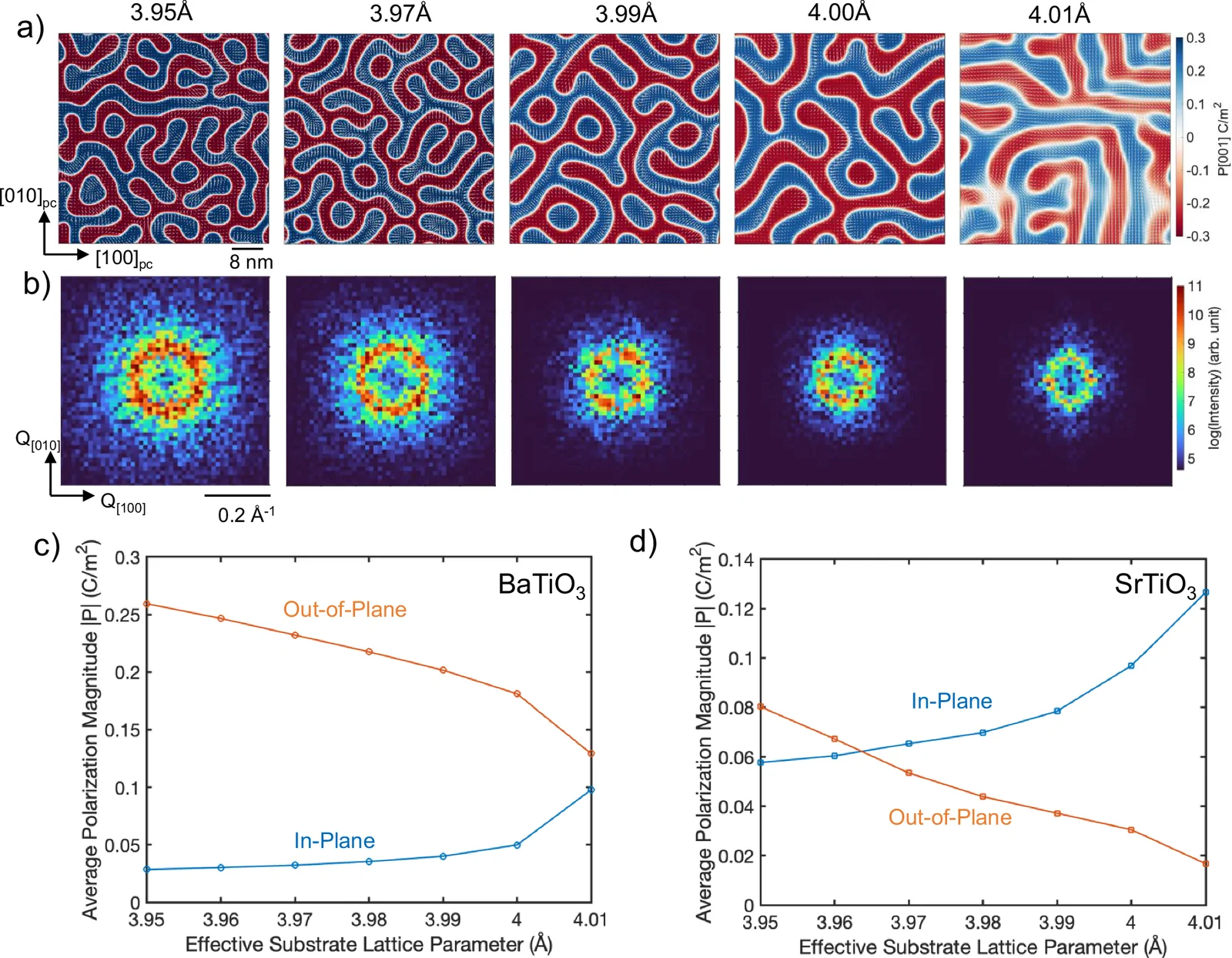
Evolution of the polarization distribution in a (12 u.c. BaTiO_3_ / 5 u.c. SrTiO_3_)_10_ superlattice on silicon Si(001) with strain. **a** Polarization slice projected onto the (001) plane, colored by the polarization component along the [001] direction, shown for a series of effective substrate lattice parameters. **b** Corresponding Fast Fourier Transform (FFT) of the (001) polarization slice for each case, highlighting the evolution of vortex ordering with strain. **c**-**d** Average polarization magnitude in the** c** BaTiO_3_ and **d** SrTiO_3_ layers as a function of the effective substrate lattice parameter.

Figure 7.a shows slices of the (001) plane with the color indicating the polarization along the out-of-plane direction ([001]_pc_) for a series of effective substrate lattice parameters, and Fig. 7.b shows the corresponding FFT. As the effective substrate lattice parameter increases, the polarization evolves from a disordered labyrinthian structure characterized by a nearly isotropic FFT (below 3.98 Å), to a partially ordered structure, which preferentially aligns along the <110> directions (between 3.98 Å and 4.00 Å), and moves to a structure with ordering along the <100> directions at 4.01 Å. The best agreement between experiments and simulations is obtained for an effective parameter of 3.99 Å (Table S1 of the Supporting Information).

The strain-dependent evolution of the average polarization magnitude for BaTiO_3_ and SrTiO_3_ is shown in Fig. 7.c, d, respectively.

The strain-induced transformations to the polar texture arise from the interplay between coupled elastic and electrostatic interactions between the BaTiO_3_ and SrTiO_3_ layers. When the effective lattice parameter is near 3.95 Å, there is compressive strain on the BaTiO_3_ layer $({\varepsilon }_{11}={\varepsilon }_{22}=-1.62\%),$ whereas the SrTiO_3_ layer is under a tensile strain $({\varepsilon }_{11}={\varepsilon }_{22}=1.22\%).$ This strain state drives the polarization towards the out-of-plane direction for the BaTiO_3_ layers and toward in-plane directions for SrTiO_3_. As the effective lattice parameter increases, the strain in the BaTiO_3_ layer is gradually relaxed, and the strain in the SrTiO_3_ becomes stronger.

Importantly, each layer does not behave independently, and the polar texture is the result of a delicate balance between primarily the elastic, gradient, Landau, and electrostatic energy in each of these layers. This interplay is reflected in the strain-dependent evolution of the average polarization magnitude for BaTiO_3_ (Fig. 7.c) and SrTiO_3_ (Fig. 7.d). For BaTiO_3_, as the effective substrate lattice parameter is increased, the out-of-plane polarization decreases due to the reduced compressive strain, which follows the expected square-root-like dependence. In contrast, SrTiO_3_ is an incipient ferroelectric, which only becomes ferroelectric under a sufficient strain. The strain-dependent behavior in SrTiO_3_ is governed by its intrinsic thermodynamic driving forces (how SrTiO_3_ would behave in isolation under the same thermodynamic conditions) and its coupling with BaTiO_3_, which results in a large deviation from the expected square-root-like dependence. Near ${a}^{{eff}}=3.95$Å, although tensile strain would typically favor an in-plane polarization in SrTiO_3_, the strong depolarization field from the BaTiO_3_ layer induces a significant out-of-plane component of polarization. As the effective substrate lattice parameter increases further, the tensile strain-driven in-plane polarization of SrTiO_3_ becomes more prominent (SrTiO_3_ under tensile strain is ferroelectric and exhibits polarization along <110> ^72,73^). This in-plane polarization breaks the degeneracy and causes the vortex tubes to preferentially align along the <110> direction. Beyond a critical lattice parameter, the BaTiO_3_ layer itself begins to develop a substantial in-plane component of polarization, along the <100>, which further alters the vortex alignment towards the <100> directions.

### Discussion on the polar textures on Si

The formation of polar vortices in BaTiO_3_/SrTiO_3_ superlattices on SrTiO_3_-buffered Si is governed by mechanical, electrostatic, and chemical boundary conditions and is the result of a delicate balance between primarily the elastic, gradient, Landau, and electrostatic energy. For superlattices grown on oxide substrates and on Si, the mechanical boundary conditions (and therefore the elastic energy) are quite different. On oxide substrates, the mechanical boundary conditions are primarily set by the lattice mismatch between the substrate and the layers. For example, polar vortices in PbTiO_3_/SrTiO_3_ superlattices have been reported when substrates such as DyScO_3_ (*a*_pc_ = 3.944 Å), Sr_1.04_Al_0.12_Ga_0.35_Ta_0.50_O_3_ (*a* = 3.915 Å) and SrTiO_3_ (*a* = 3.905 Å) are used. These substrates have lattice mismatches of 1.01%, 0.28%, and 0.03%, respectively, relative to bulk PbTiO_3_ (*a* = 3.904 Å), imposing a tensile strain to the ferroelectric layer, which reduces the anisotropy of the polarization vector and allows it to rotate away from the high-symmetry crystallographic out-of-plane axis. Therefore, moderate tensile strain is a key ingredient for the formation of polar vortices.

For perovskite oxides on Si, the epitaxial strain is not tensile but compressive, since the atomic distance along the [110] direction of Si, relevant to the epitaxy, is of$\,\frac{{a}_{{Si}}}{\sqrt{2}}$ = 3.84 Å. The in-plane lattice mismatch with a perovskite structure is more than −4%. Such a large value explains why the direct growth of epitaxial BaTiO_3_ on Si is not possible but requires the use of a buffer layer like SrTiO_3_^55^. However, there is also a tensile strain, which arises from the large difference in thermal expansion coefficients between the perovskite oxides (∼8.7×10^−6^/˚C for SrTiO_3_^74^; ∼10.8×10^−6^/˚C for BaTiO_3_^75^) and Si (∼2.6×10^−6^/˚C^76^). Note that the formation of an amorphous SiO_x_ layer between the SrTiO_3_ buffer and the Si substrate is also a contributor to the strain^77,78^. During the growth of the superlattices at 650 ˚C under p(O_2_) = 5.0 × 10^−7^ Torr, oxygen diffuses down to the Si substrate, leading to an interfacial amorphous SiO_x_ or silicate layer. Upon cooling to room temperature, the thermal contraction mismatch between the perovskite layers and Si generates significant tensile stress. We believe that the amorphous SiO_x_ interface mechanically decouples partially the superlattice from the Si substrate, and allows it to retain a residual tensile strain, which give rise to the formation of polar vortices in the BaTiO_3_/SrTiO_3_ superlattices. Notably, BaTiO_3_/SrTiO_3_ superlattices grown on SrTiO_3_ substrates, which impose compressive strain (−2.27% relatively to bulk BaTiO_3_), do not exhibit periodic polar vortices but only local whirling dipoles^46^, which we attribute to the absence of a tensile component in the system. This is confirmed by our own experiments on SrTiO_3_ substrates. These results underline the importance of the contribution of a tensile stress in the stabilization of long-range ordered polar textures in superlattices and demonstrate that growth on Si provides the required tensile stress.

Surprisingly, the polar vortex tubes in the BaTiO_3_/SrTiO_3_ superlattices are ordered along the <110> directions of the perovskite structure as shown both by the RSMs and STEM in-plane stripe patterns and confirmed by phase field modelling. This is in contrast to the stripe patterns in PbTiO_3_/SrTiO_3_ superlattices and PbTiO_3_/SrRuO_3_ trilayers, with polar vortex tubes^6,8,31,79,80^ and polar waves^41^ ordered along the <100> directions of PbTiO_3_.

In bulk, BaTiO_3_ exhibits polarization along the <110> directions in its low-symmetry orthorhombic phase, which is stable in the temperature range 183 − 278 K and along the <001> directions in the tetragonal phase (278 < T < 393 K)^81^. Bulk SrTiO_3_ is cubic at room temperature and does not exhibit an orthorhombic phase but a cubic to tetragonal antidistortive phase transition at 105 K^82^. However, under tensile strain, SrTiO_3_ is orthorhombic and ferroelectric. Experimental studies on BaTiO_3_/SrTiO_3_ superlattices have shown that SrTiO_3_ layers adopt an orthorhombic crystal structure at room temperature and that BaTiO_3_ layers keep a tetragonal structure^83–85^. Note that our Raman measurements on the superlattice and on a *c*-oriented single crystal (Fig. S5 of the Supporting Information) indicate also that the BaTiO_3_ layers in the superlattice are in the tetragonal structure (strong contribution of the B_1_ + E(LO + TO) modes).

The lattice parameter profiles determined from the STEM images and from the GPA analysis indicate that the in-plane parameters of BaTiO_3_ and SrTiO_3_ layers are identical (BaTiO_3_ is fully strained on SrTiO_3_ and vice versa) and that the value does not vary much throughout the thickness of the superlattice. The in-plane lattice parameter ranges between 3.97 and 4.00 Å depending on the lateral location chosen on the lamellae (corresponding to a bi-axial tensile strain for SrTiO_3_ and a bi-axial compressive strain for BaTiO_3_). These results are in qualitative good agreement with the phase field modelling, which predicts vortices aligned along <110> directions for an effective substrate parameter in the range 3.98 − 4.00 Å.

The temperature-dependent RSM measurements show that the vortex periodicity decreases from 6.5 to 5.5 nm upon heating and disappears at the Curie temperature *T*_c_ of ~450 K. The same periodicity is recovered when cooling back to room temperature. High-temperature STEM studies would be of interest to explore whether there are any microscopic changes during the heating or cooling processes. The vortices stabilized in PbTiO_3_/SrTiO_3_ superlattices on DyScO_3_ have shown a temperature-dependent evolution of the polar pattern. For instance, in a 16 u.c. PbTiO_3_/16 u.c. SrTiO_3_ superlattice, a transition was observed^37^ from a mixed vortex/ *a*_1_/*a*_2_ polar pattern at room temperature to purely *a*_1_/*a*_2_ domains at 473 K (and to a paraelectric phase at 598 K). A thermally activated transition from vortex phase to polar wave and polar solitons was reported for a 12 unit-cell PbTiO_3_/10 unit-cell SrTiO_3_ superlattice^41^. In the future, low-temperature STEM and Raman experiments would be of particular interest to see whether the polar pattern changes or whether it is locked by the substrate’s clamping, when cooling down through the expected phase transitions of BaTiO_3_, from tetragonal to orthorhombic (∼280 K) and from orthorhombic to rhombohedral (∼190 K) structures.

The electrical behavior of the superlattices could not be investigated by macroscopic measurements due to large leakage currents in BaTiO_3_. Switching spectroscopy PFM shows a butterfly shape in the amplitude signal (Fig. S13.a, b of the Supporting Information), and a hysteretic phase with a change of 180º, which is the typical response of a ferroelectric material. However, such a response can also be obtained in non-ferroelectric materials^86^. By scanning the sample surface with $\pm \,$6 V DC tip bias in adjacent regions (Fig. S13.c, d of the Supporting Information), we observe a 180º phase difference for regions poled with opposite biases. The amplitude signal shows a zero response at the interface regions, which could correspond to ferroelectric domain walls. However, the written regions exhibit a strong contrast in amplitude, which may originate from electrostatic, charge injection, ionic and/or electrochemical contributions^86^, effects that are likely enhanced by the fact that the superlattice terminates with a SrTiO_3_ layer. Hence, the PFM measurements do not allow to conclude on the ability to unwind the vortices under an applied electrical field.

In conclusion, we have demonstrated the realization of periodic polar vortices in (4.8 nm BaTiO_3_/2.0 nm SrTiO_3_)_10_ superlattices grown on SrTiO_3_-buffered silicon that were previously exclusive to PbTiO_3_/SrTiO_3_ systems on oxide substrates. The vortices are ordered along the <110> crystallographic directions of BaTiO_3_ and extend in tubes in the perpendicular directions. This particular ordering is due to the coupled elastic and electrostatic interactions between BaTiO_3_ and SrTiO_3_ layers: the tensile strain in SrTiO_3_ induces an in-plane polarization along its <110> directions, which in turn induces the vortices to align along the same directions.

Our observations demonstrate that topologically non-trivial polarization textures can be realized on silicon, a technologically relevant platform, using lead-free materials such as BaTiO_3_. We believe that the tensile strain imparted by silicon at the growth temperature (650˚C) and which is maintained upon cooling the samples to room temperature, is a key ingredient to stabilize the ordered vortex tubes.

This work marks a first step for bringing functionality of topological polar textures into the world of nanoelectronics, but several challenges remain before such materials could be integrated into practical CMOS nanoscale devices. Further studies are required to evaluate the impact of patterning and etching processes on the integrity of the topological textures. If thermal budget constraints restrict high-temperature oxide growth to front-end-of-line architectures, approaches such as wafer bonding can be used for back-end-of-line integration^87^. Ongoing and future work will focus on reducing the leakage currents in BaTiO_3_, which is a critical step toward assessing their ferroelectric properties in micro- and nano-scale capacitors. The behavior and potential control of the vortices under electric field is of particular interest. There are several questions to be tackled: can the vortices be “switched” (from clockwise to anticlockwise and vice versa), can they be unwound, can they be moved. Another perspective involves the search for conditions to stabilize polar skyrmions, and the investigation of the chirality and electrical response of the different polar textures on silicon.

## Methods

### Growth of BaTiO_3_/SrTiO_3_ superlattices on an epitaxial SrTiO_3_ buffer on silicon

The Si wafers were treated with UV/ozone at 100˚C for 10 min using an ozone-generator to remove organic contaminants. The Si wafers were then immediately loaded into the load-lock chamber of a DCA R450 MBE system and outgassed at 200˚ C for 2 h. The growth of the 4 nm epitaxial SrTiO_3_ buffer involved four steps monitored in situ by reflection high-energy electron diffraction (RHEED). First, the native SiO_x_ layer on the Si surface was removed by sending a Sr flux and annealing under ultrahigh vacuum at 870˚C until the 2×1 reconstruction of the Si surface was observed by RHEED. Second, the temperature was lowered to 380˚C and additional metallic Sr was deposited, followed by a vacuum anneal at 670˚C to have ½ monolayer of Sr (row of Sr atoms in-between the dimer rows of Si atoms). The 2×1 Sr surface observed by RHEED indicated that the passivation steps were completed. Third, SrTiO_3_ was grown by co-depositing Sr and Ti under an oxygen partial pressure of 5.0 × 10^−8^ Torr at 380˚C. Finally, the SrTiO_3_ layer was annealed at 490˚C in vacuum to improve the crystallinity. The (12 u.c. BaTiO_3_ / 5 u.c. SrTiO_3_)_10_ superlattice was grown by co-evaporating Sr or Ba and Ti at 650˚C under a molecular O_2_ partial pressure of 5.0 × 10^−7^ Torr. The sample was slowly cooled to 200˚C at an O_2_ partial pressure of 1.0 × 10^−5^ Torr.

### Structural analysis by X-ray diffraction

X-ray diffraction patterns were recorded with Cu Kα radiation (*λ* = 1.5405 Å) using a Panalytical X’Pert Pro diffractometer. Synchrotron-based reciprocal space maps were measured to obtain information on the in-plane ordering of ferroelectric domains. The experiments were carried out at the P08 high-resolution diffraction beamline at Deutsches Elektronen Synchrotron (DESY) in Hamburg, providing a flux of 10^11^ − 10^12^ photons per second. The X-ray energy was fixed to 10 keV. Reciprocal space maps were measured using a Pilatus 100k pixel detector.

### Raman spectroscopy

Raman spectra were acquired using a Horiba HR-Evolution Raman spectrometer in back-scattered geometry, equipped with a line grating of 1800 l/mm and using a UV 325 nm continuous-wave laser. For room-temperature Raman measurements, a 40× objective (NA = 0.49) was used. Temperature-dependent Raman measurements were performed using a 20× objective (NA = 0.39) and a Microstat Hires environmental chamber. The temperature of the chamber was controlled with a MercuryiTC controller.

### Piezoresponse force microscopy

PFM measurements were performed with a NX10 microscope from Park Systems, in the dual frequency resonance tracking (DFRT) mode (external UHFLI lock-in amplifier from Zurich Instruments). Platinum/iridium-coated tips (PPP-EFM, Nanosensors) with a force constant of ∼2.8 N/m were used. For the writing/reading experiments, the DC voltage was applied to the tip, and the AC modulation (0.6 V, ∼350 kHz) was applied to the chuck.

### Focused ion beam preparation of the lamellae

The FIB preparations of the STEM lamellae were performed at three different locations. Cross-sectional and planar TEM lamellae were prepared using Ga⁺-source FIB-SEM dual-beam systems (Thermo Fisher Helios NanoLab G3 and Helios 600i). Specimens were oriented along the [110] or [100] zone axis of the Si substrate. The superlattice surface was protected by Electron Beam Induced Deposition (EBID) SiO_x_ or Pt followed by milling at 30 keV. Lamellae were extracted using standard lift-out procedures and mounted on Cu grids or half-grids.

To minimize bending and curtaining effects, some cross-sectional lamellae were prepared by the non-standard upside-down preparation technique, i.e. with an ion beam milling direction from the Si substrate to the surface^88^. The preparation was finalized with low energy milling steps at 5 and 2 keV (and down to 1 keV in some cases), minimizing the damage layer on the lamella sidewalls and achieving an estimated lamella thickness in the range of 20 − 40 nm.

For planar TEM specimens, samples were mechanically thinned from the substrate side to a thickness of approximately 40 µm and affixed to a copper half-ring. Electron-transparent windows, each approximately 30 × 30 µm^2^ in size, were milled into the sample by FIB using stepwise reduction of the current (0.44 nA down to 10 pA) and voltage (30 kV down to 2 kV) and progressive increase of the milling angle (1° up to 6°).

### Scanning transmission electron microscopy

The superlattices were characterized using a monochromated and probe-aberration-corrected STEM Nion HERMES transmission electron microscope. High-angle annular dark field scanning transmission electron microscopy (HAADF-STEM) images were recorded at 200 kV with a convergence angle of 18 mrad. For every image, 5 frames were recorded with short dwell time per pixel (0.5 µs/px) to reduce the influence of sample drift. The frames were subsequently aligned and integrated. For the analysis of STEM images, a Wiener noise filter was applied.

For calculating the Ti-displacement maps, the positions of Ba, Sr, and Ti ions were determined using the python-based software package Atomap^89^. The shift of the Ti ion was defined as the distance between the Ti position and the center of the neighboring Ba-positions (Sr-positions).

Geometric phase analysis (GPA)^90^ was performed using the ImageEval software^91^. The strain was determined from the 001 and $\bar{1}$01 reflections of BaTiO_3_/SrTiO_3_ in the fast Fourier-transform. The Si substrate was used as the unstrained reference. The Gaussian mask was set to be 1/3 of the *g*-vector distance.

### Phase field simulations

The phase-field model for ferroelectrics is used to simulate the temporal evolution of the polarization in BaTiO_3_/SrTiO_3_-based superlattices. In the phase-field model the time evolution of the polarization vector field is described by the time-dependent Ginzburg-Landau equation:

1 $${\gamma }_{{ij}}\frac{\partial {P}_{j}({r}_{i},t)}{\partial t}=-\frac{\delta F}{\delta {P}_{i}\left({r}_{i},t\right)},$$

where ${P}_{i}$ represents the spontaneous polarization (measured in C/m^2^), ${r}_{i}$ represents the position in space,t is time, and ${\gamma }_{{ij}}$ is polarization damping coefficient relating to the rate of polarization relaxation.

The total free energy functional (F) contains the following energy-density terms.

2 $$F={\int }_{\!\!\!\!V}\left({f}_{{Landau}}+{f}_{{Gradient}}+{f}_{{Elastic}}+{f}_{{Electric}}\right)\,{dV}.$$

Following the Landau-Ginzburg-Devonshire theory of ferroelectrics^92^, the Landau free-energy density $({f}_{{Landau}})$ describes the intrinsic stability of the ferroelectric phase with respect to the paraelectric phase and is written as a Taylor expansion of the polarization about the paraelectric reference phase $\left({P}_{i}=0\right)$:

3 $${f}_{{Landau}}={a}_{{ij}}\left(T\right){P}_{i}^{L}{P}_{j}^{L}+{a}_{{ijkl}}{P}_{i}^{L}{P}_{j}^{L}{P}_{k}^{L}{P}_{l}^{L}+{a}_{{ijklmn}}{P}_{i}^{L}{P}_{j}^{L}{P}_{k}^{L}{P}_{l}^{L}{P}_{m}^{L}{P}_{n}^{L}+{a}_{{ijklmnop}}{P}_{i}^{L}{P}_{j}^{L}{P}_{k}^{L}{P}_{l}^{L}{P}_{m}^{L}{P}_{n}^{L}{P}_{o}^{L}{P}_{p}^{L},$$

where ${a}_{{ij}}$, ${a}_{{ijkl}}$, ${a}_{{ijklmn}}$, and ${a}_{{ijklmnop}}$ are the dielectric stiffness coefficients measured under constant stress conditions. For BaTiO_3_, an 8^th^ order expansion is used^93^ and for SrTiO_3_ a 4^th^ order expansion is used^72^. All material property coefficients are given in Table S2 of the Supporting Information.

The gradient energy density is

4 $${f}_{{gradient}}=\frac{1}{2}{G}_{{ijkl}}\frac{\partial {P}_{i}}{\partial {x}_{j}}\frac{\partial {P}_{k}}{\partial {x}_{l}}$$

where ${G}_{{ijkl}}$ is the gradient energy tensor where the non-zero coefficients are chosen to be ${G}_{11}=0.8,\,{G}_{22}=-0.8$, and ${G}_{44}=0.8$, and the units are normalized by ${\alpha }_{1}{l}_{o}^{2}$ where ${\alpha }_{1}$ is the first Landau expansion coefficient and ${l}_{o}$ is the grid spacing (0.4 nm).

The elastic energy density is

5 $${f}_{{elas}}=\frac{1}{2}{c}_{{ijkl}}\left({\varepsilon }_{{ij}}-{\varepsilon }_{{ij}}^{0}\right)\left({\varepsilon }_{{kl}}-{\varepsilon }_{{kl}}^{0}\right)$$

where ${c}_{{ijkl}}$ is the elastic stiffness tensor, ${\varepsilon }_{{ij}}$ is the total strain (using the stress-free paraelectric phase as a reference), and ${\varepsilon }_{{ij}}^{0}$ is the eigenstrain which is related to the polarization by

6 $${\varepsilon }_{{ij}}^{0}={Q}_{{ijkl}}{P}_{k}{P}_{l}$$

where ${Q}_{{ijkl}}$ is the electrostrictive tensor. The elastic stiffness and electrostrictive coefficients are given in Table S1 of the Supporting Information. For simplicity, we neglect the inhomogeneity in the elastic stiffness tensor between the BaTiO_3_ and SrTiO_3_ layers. The total strain ${\varepsilon }_{{ij}}$ is found by solving the mechanical equilibrium equation $\nabla \cdot {\sigma }_{{ij}}=0$, where ${\sigma }_{{ij}}={c}_{{ijkl}}\left({\varepsilon }_{{ij}}-{\varepsilon }_{{ij}}^{0}\right).$

The electrostatic energy density is

7 $${f}_{elec}=-{E}_{i}{P}_{i}-\frac{1}{2}{\epsilon }_{0}{\kappa }_{{ij}}^{b}{E}_{i}{E}_{j}$$

where ${\epsilon }_{0}$ is the vacuum permittivity and ${\kappa }_{{ij}}^{b}$ is the background dielectric constant which is chosen as 10 and is isotropic. The electric field is found by solving the electrostatic equilibrium condition $\nabla \cdot \left({\epsilon }_{0}{\kappa }_{{ij}}^{b}{E}_{j}+{P}_{i}\right)=0$. Further details on solving the elastic and electro-static equilibrium equations may be found in Ref.^94^.

In the simulation, we define the BaTiO_3_/SrTiO_3_ superlattice by spatially varying the material properties using a sharp interface description. We treat the interface between the BaTiO_3_ and SrTiO_3_ layers as elastically coherent, and we approximate the effect of the Si substrate and SrTiO_3_ buffer layer on the strain conditions by introducing an effective substrate lattice parameter and assume that the BaTiO_3_ and SrTiO_3_ layers are strained such that the average strain for each layer matches the effective substrate lattice parameter. This allows us to calculate the strain using the equivalent cubic lattice constant for BaTiO_3_ ${a}_{{BaTiO}3}^{{eq},\,{cubic}}=4.015{{\mathrm{\AA}}}$ and the cubic lattice constant for SrTiO_3_ ${a}_{{SrTiO}3}^{{eq},\,{cubic}}=3.905{{\mathrm{\AA}}}$. We assume that there is no in-plane shear strain such that:

8 $${\varepsilon }_{11}^{{BaTi}{O}_{3}}={\varepsilon }_{22}^{{BaTi}{O}_{3}}=\frac{{{a}^{{eff}}-a}_{{BaTiO}3}^{{eq},{cubic}}}{{a}_{{BaTiO}3}^{{eq},{cubic}}},{\varepsilon }_{11}^{{SrTi}{O}_{3}}={\varepsilon }_{22}^{{SrTi}{O}_{3}}=\frac{{{a}^{{eff}}-a}_{{SrTiO}3}^{{eq},{cubic}}}{{a}_{{SrTiO}3}^{{eq},{cubic}}}.$$

A system size of $128\Delta {x}_{1}\times 128\Delta {x}_{2}\times 100\Delta {x}_{3}$ is used. The thicknesses of the BaTiO_3_ and SrTiO_3_ layers are set to $12\Delta {x}_{3}$ and $5\,\Delta {x}_{3}$ respectively. The substrate thickness is set to $12\Delta {x}_{3}$ and a $4\Delta {x}_{3}$ layer of air is placed near the top of the simulation. Periodic boundary conditions were applied in the in-plane directions. In all simulations $\Delta {x}_{1}=\Delta {x}_{2}=\Delta {x}_{3}=0.4{nm}$.

To initiate the simulation, random noise was applied to the polarization at each vector point ($|{P}^{{noise}}|=0.001\,C/{m}^{2}$), then the simulation evolved until a steady state condition was reached. All phase-field simulations were performed using the μPro software.

## Supplementary information

Supplementary Information

Transparent Peer Review file

## Data Availability

The raw experimental data and modeling data generated in this study have been deposited in a Zenodo repository available at https://doi.org/10.5281/zenodo.21885835 (Ref.^95^). Data presented in the Supporting Information is available upon request.

## Code availability

The MuPRO code package used in this work to carry out the phase-field simulations can be obtained after a user license is authorized by the MuPRO company (https://muprosoftware.com).
