# Supplementary material for: Periodic polar vortices in BaTiO3/SrTiO3 superlattices monolithically grown on silicon
Source: Nat Commun. 2026 Sep 8;17:9648. doi: 10.1038/s41467-026-77270-0 (PMC13558690; doi:10.1038/s41467-026-77270-0)
Supplement: Supplementary file 1 [file 41467_2026_77270_MOESM1_ESM.pdf]

Supporting Information

Periodic polar vortices in $BaTiO_3/SrTiO_3$ superlattices monolithically grown on silicon

Valentin Väinö Hevelke[1,2], Ines Häusler[1,3], Aiden Ross[4], Israel Ibukun Olaniyan[1,2], Sven Wiesner[1], Minh Anh Luong[1], Leifeng Zhang[5], Sylvie Schamm-Chardon[5], Kristiane Elsner[3], Benedikt Haas[3], Adnan Hammud[6], Florian Bertram[7], Christoph T. Koch[3], Long-Qing Chen[4], Dong-Jik Kim[1], Catherine Dubourdieu[1,2]

1. Helmholtz-Zentrum Berlin für Materialien und Energie GmbH, Hahn-Meitner-Platz 1, 14109 Berlin, Germany
2. Freie Universität Berlin, Physical Chemistry, Physical and Theoretical Chemistry, Arnimallee 22, 14195 Berlin, Germany
3. Humboldt-Universität zu Berlin, Department of Physics, Newtonstraße 15, 12489 Berlin, Germany
4. Department of Materials Science and Engineering and Materials Research Institute, The Pennsylvania State University, University Park, PA 16802, USA
5. CEMES-CNRS and Université de Toulouse, 29 rue Jeanne Marvig, 31055 Toulouse, France
6. Department of Inorganic Chemistry, Fritz Haber Institute of the Max Planck Society, 14195 Berlin, Germany
7. Deutsches Elektronen-Synchrotron (DESY), 22607 Hamburg, Germany

Energy-dispersive X-ray spectroscopy (EDX) data were acquired using a Thermo Fisher Scientific TFS Spectra 300 microscope operated at an accelerating voltage of 300 kV. A probe convergence angle of 21.4 mrad and a probe current of 150 pA were used throughout the measurements. The generated X-ray signals were collected with an Ultra-X six-segment detector using a dwell time of 25 µs per pixel. A total of 500 frames were acquired, and drift correction was applied during acquisition. Elemental quantifications ($BaTiO_3$ and $SrTiO_3$) were performed using the O–K, Ti–K, Sr–K, and Ba–K X-ray emission lines.

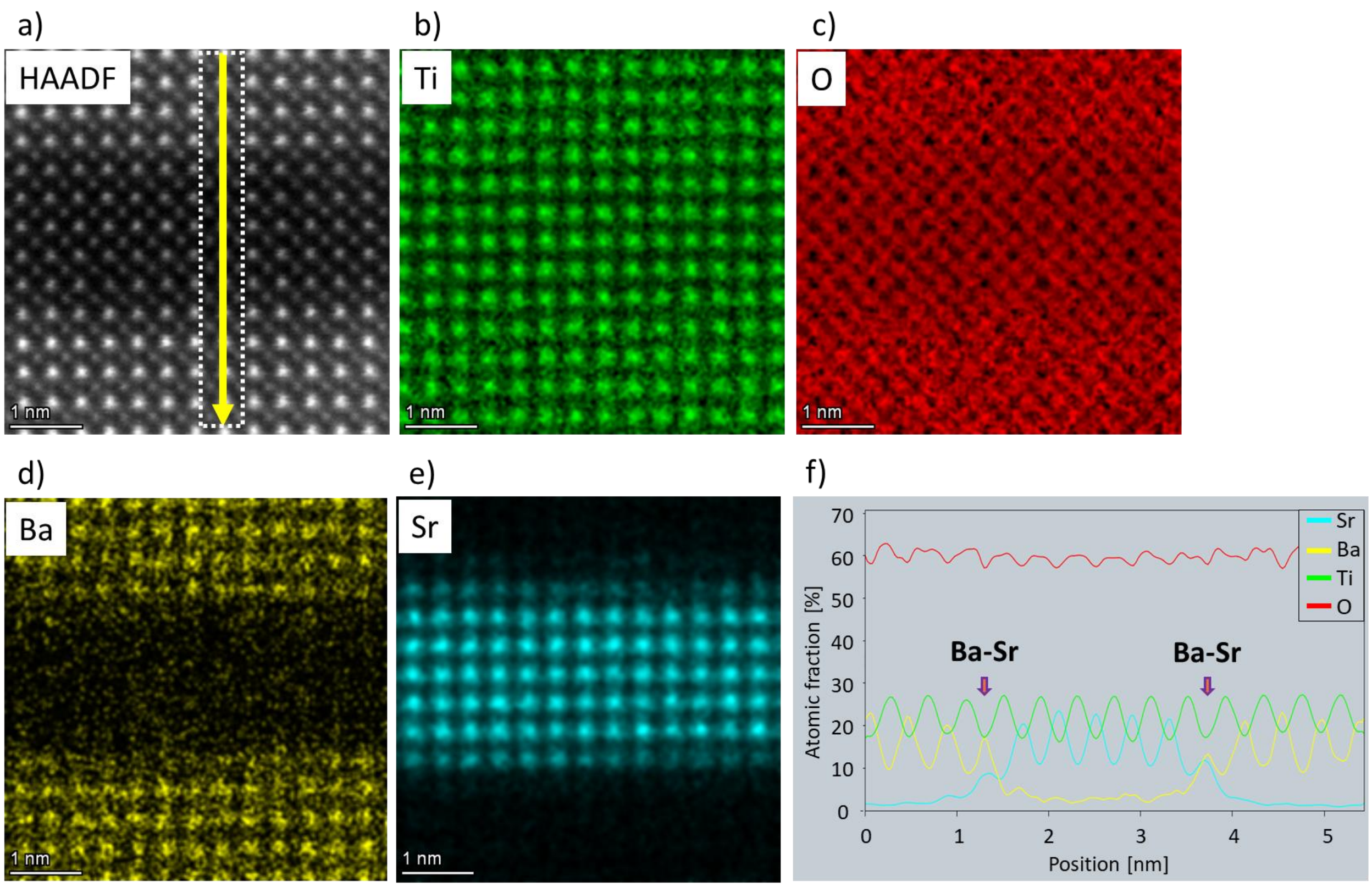

Figure S1: **EDX analysis of a (12 u.c. $BaTiO_3$ / 5 u.c. $SrTiO_3$)$_{10}$ superlattice on Si (001).** a) HAADF-STEM image together with corresponding EDX elemental maps of b) Ti-L, c) O-K, d) Ba-K, and e) Sr-K. f) EDX line profiles extracted along the line indicated in panel a), showing the spatial distribution of the constituent elements across the $BaTiO_3$/$SrTiO_3$ interfaces.

The HAADF STEM image of the analyzed region is shown in Figure S1.a. The elemental maps and corresponding intensity line-profile indicate a homogeneous distribution of Ti and O

throughout the superlattice (Figure S1.b,c). The Ba and Sr maps are shown in Figure S1.d,e. At both types of interfaces - $BaTiO_3$ to $SrTiO_3$ and $SrTiO_3$ to $BaTiO_3$ - the first unit cell exhibits detectable signals from both Ba and Sr, indicating a mixed A-site composition. Such interfacial mixing is expected during our MBE growth, since the growth does not proceed via perfectly terminated two-dimensional monolayers at the atomic scale; the layers were grown by co-deposition of Ba and Ti for $BaTiO_3$ (and Sr and Ti for $SrTiO_3$) while the sample was continuously rotated during deposition to ensure uniform growth across the 2-inch Si wafer. Therefore, the growth was not monitored using RHEED oscillations to precisely terminate each individual monolayer. Consequently, when the deposition of the $SrTiO_3$ layer is stopped, the growth front is not necessarily atomically complete and may contain, moreover, both TiO_2 and SrO terminations. During the subsequent growth of $BaTiO_3$, Ba atoms are incorporated onto this non-ideal surface, naturally resulting in a mixed A-site composition over approximately one unit cell at the $SrTiO_3/BaTiO_3$ interface. The same holds true for the growth of $SrTiO_3$ on top of $BaTiO_3$ resulting in the intermixing at the $BaTiO_3/SrTiO_3$ interface.

The EDX line scan across the interfaces (Figure S1.f) reveals a weak Ba signal within the $SrTiO_3$ layers, suggesting a possible minor content of Ba in $SrTiO_3$. However, the magnitude of this signal remains low and should be interpreted with caution. In thin superlattices with only five unit cells of $SrTiO_3$ sandwiched between $BaTiO_3$ layers, electron beam delocalization, probe broadening, and multiple scattering effects contribute to apparent elemental overlap in atomic-resolution EDX maps. In addition, the substantially high excitation energy of the Ba–K edge results in weak X-ray intensity and low counting statistics, which introduces additional uncertainties in peak fitting and quantitative analysis. We did not use the Ba–L line as there is a strong overlap between the Ba–L and Ti–K emission lines, which introduces too much uncertainty during background subtraction and peak deconvolution.

The weak Ba signal inside the $SrTiO_3$ layers is likely related to multiple scattering effects (note that no Sr is observed in the 12 u.c. of $BaTiO_3$ except at the interfaces).

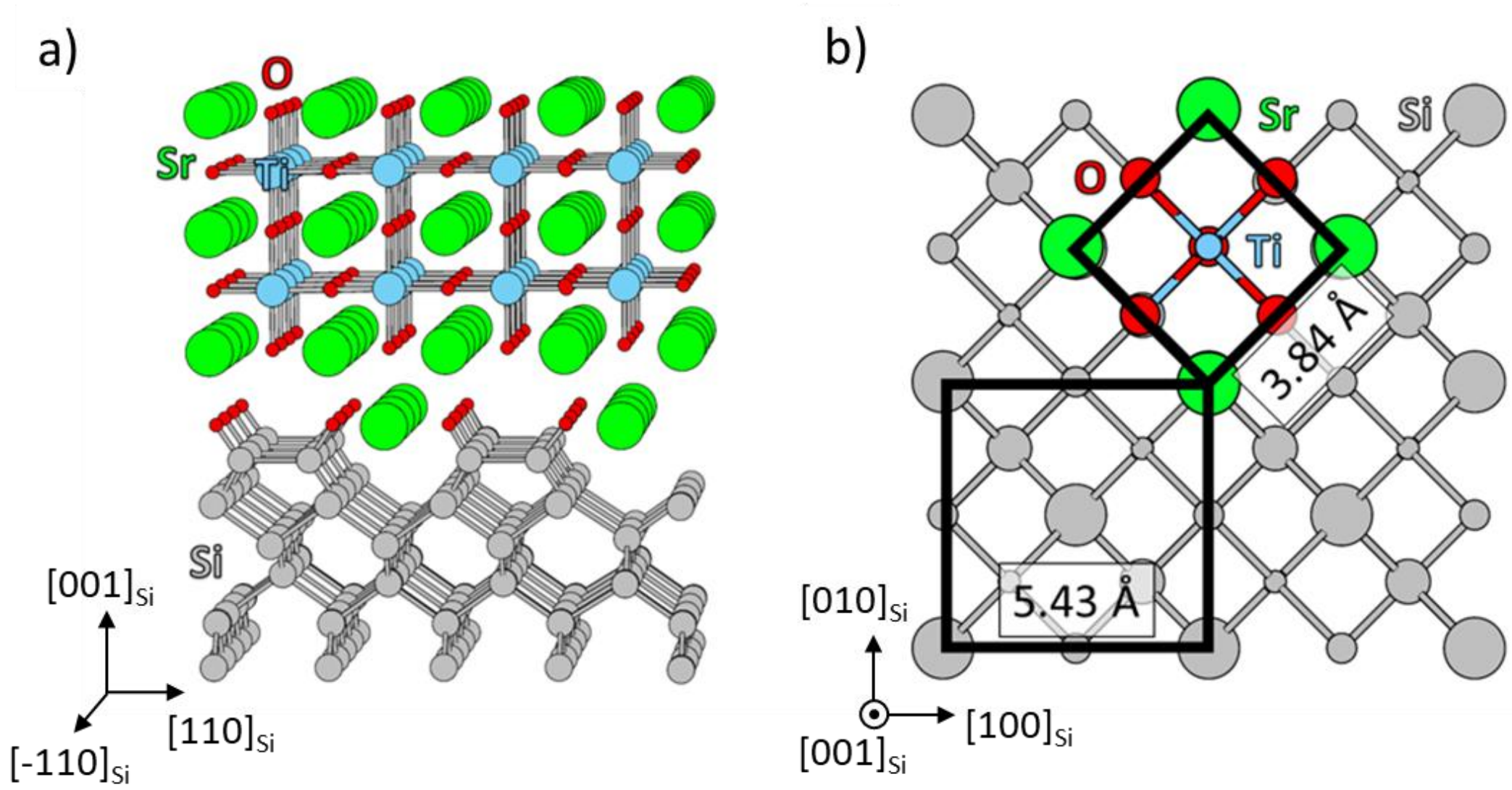

Figure S2: Atomic structure of epitaxial $SrTiO_3$ on Si (001). a) Side-view and b) top-view of the atomic structure of epitaxial $SrTiO_3$ on Si (001). The unit cell of $SrTiO_3$ is rotated 45˚ with respect to the unit cell of Si to match the 1x1 Si surface unit cell. The size circles representing the Si atoms in b) indicates the vertical positions with respect to the surface. Figure a) and b) were adapted from Ref.[1] and Ref.[2], respectively.

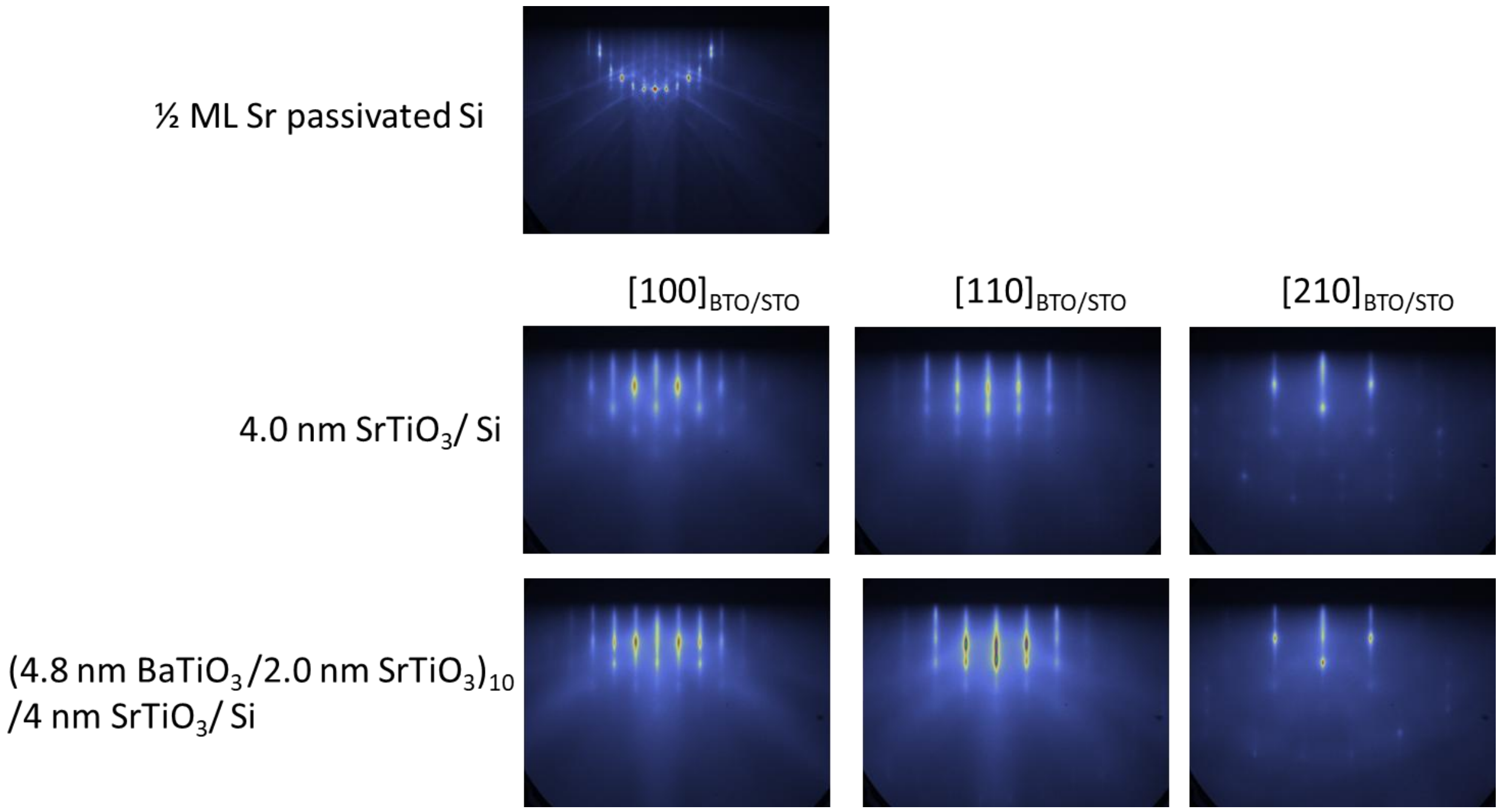

Figures S3: RHEED during superlattice growth by molecular beam epitaxy on silicon. RHEED images taken during different stages of the growth of a (12 unit-cells $BaTiO_3$/ 5 unit-cells $SrTiO_3$)$_{10}$ superlattice on $SrTiO_3$-buffered Si (001): ½ ML Sr-passivated Si (top row), 4.0 nm $SrTiO_3$ buffered Si (middle row), (4.8 nm $BaTiO_3$ / 2.0 nm $SrTiO_3$)$_{10}$ / 4.0 nm $SrTiO_3$ / Si (bottom row).

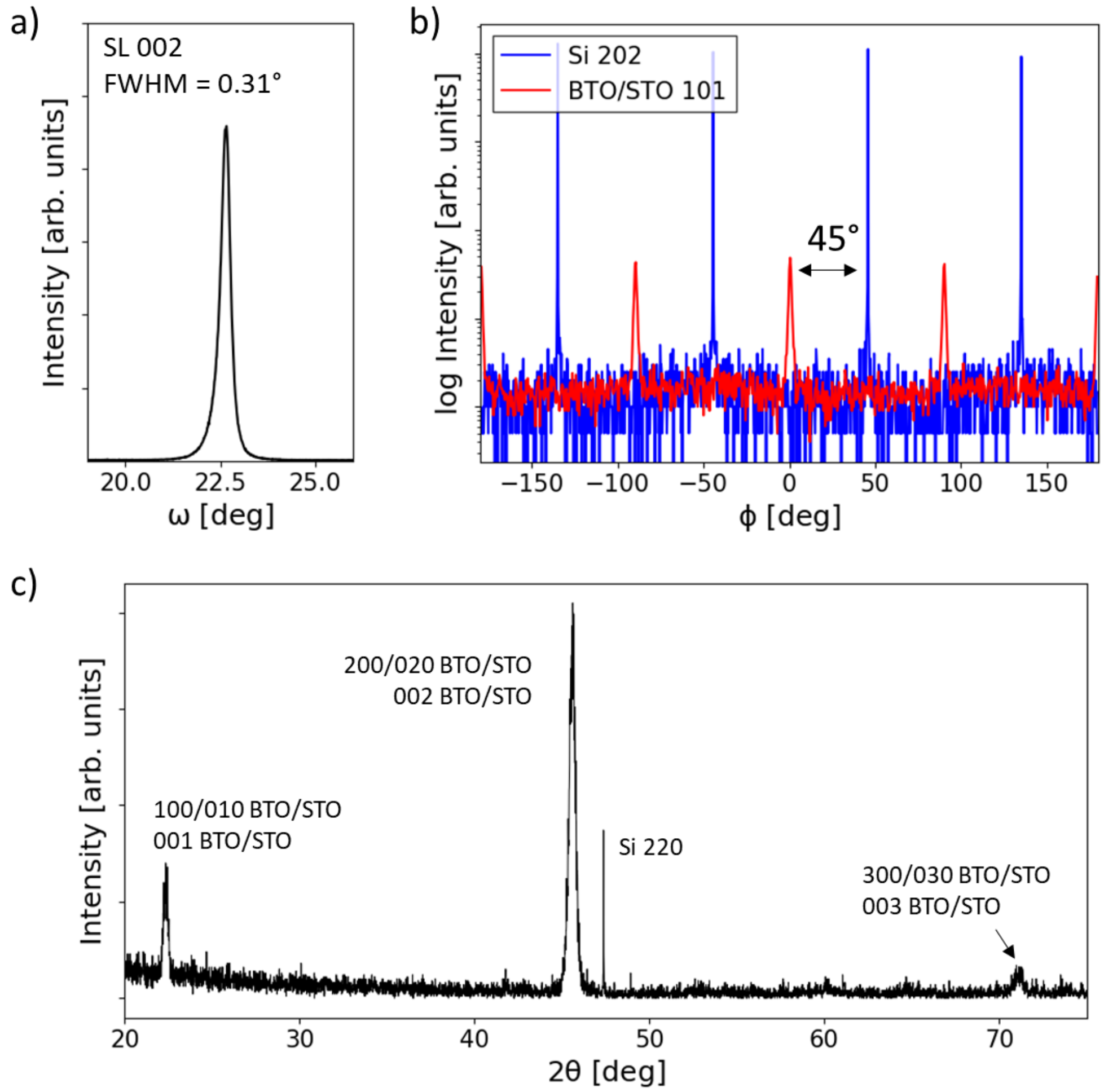

Figure S4: X-Ray diffraction of a (12 u.c. $BaTiO_3$ / 5 u.c. $SrTiO_3$)$_{10}$ superlattice on Si (001). a) Rocking curve of the $SrTiO_3$/$BaTiO_3$ 002 reflections b) ϕ scans of the Si 202 reflection and the $SrTiO_3$/$BaTiO_3$ 101 reflections (χ = 45˚) showing the 45˚ in-plane rotation of the perovskite unit cell with respect to the Si unit cell. c) In-plane X-ray diffraction scan with a χ = 89.7˚ for grazing incidence.

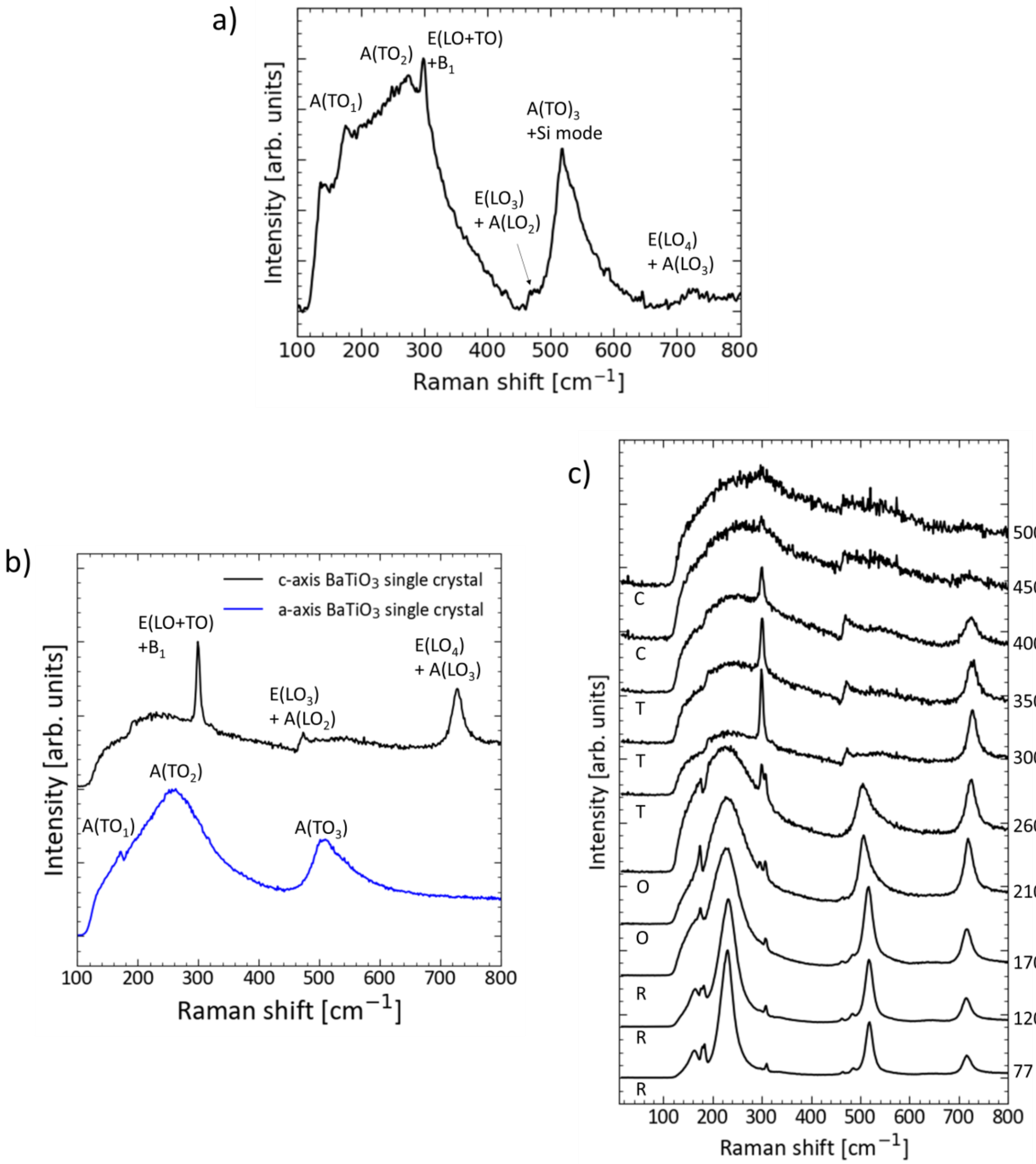

Figure S5: Raman spectroscopy measurements. a) Raman spectrum measured at 300 K on a (12 u.c. $BaTiO_3$ / 5 u.c. $SrTiO_3$)$_{10}$ superlattice on Si(001). b) Reference Raman spectra measured on *c*- and *a*-oriented $BaTiO_3$ single crystals at 300 K. c) Temperature dependent Raman measurements of a *c*-oriented $BaTiO_3$ single crystal showing the rhombohedral phase (R, 77 K to 170 K), orthorhombic phase (O, 210 K to 260 K), tetragonal phase (T, 300 K to 350 K) and cubic phase (C, 400 K to 500 K) of $BaTiO_3$.

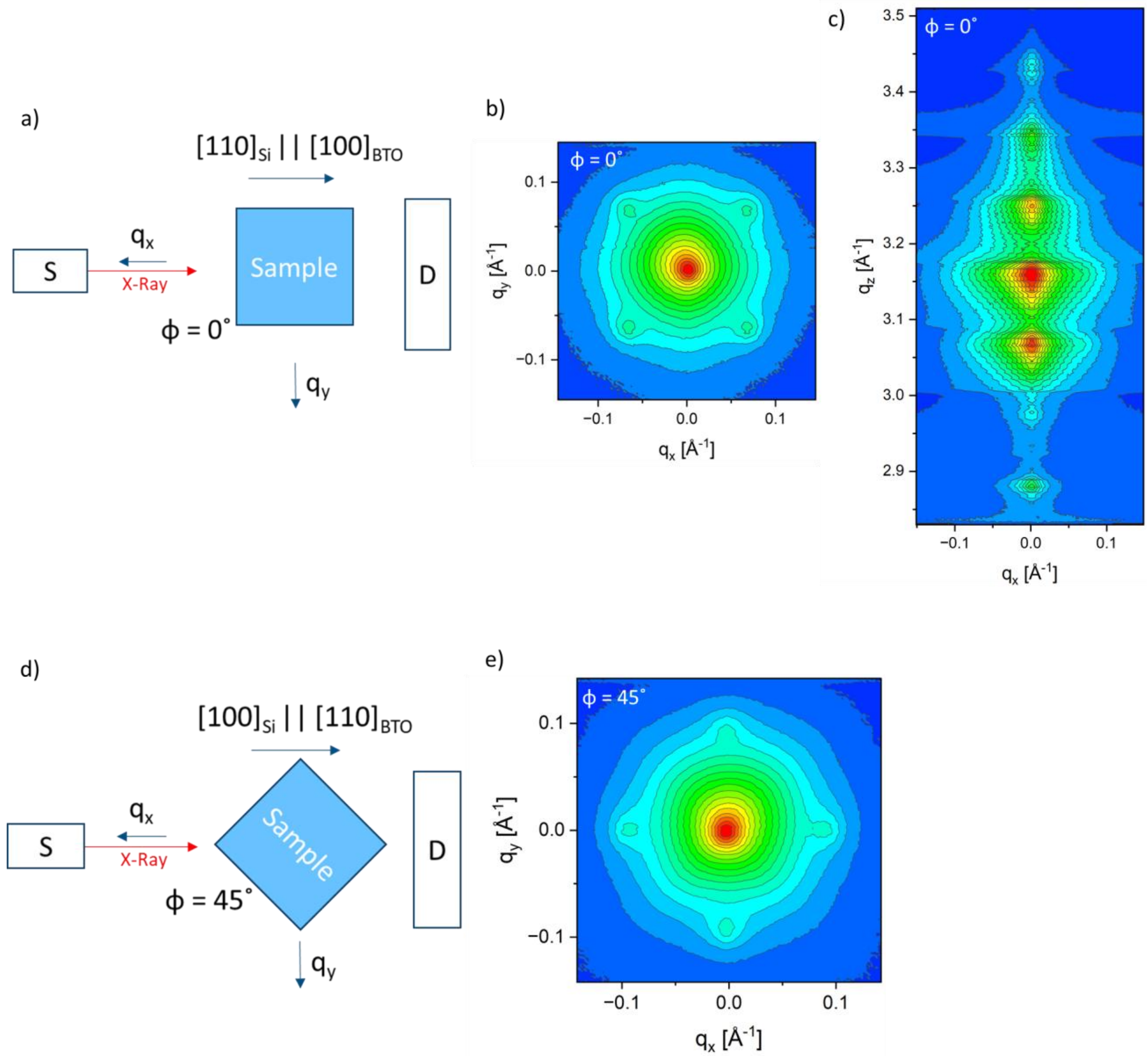
a)
[110]Si | | [100]BTO
qx
S
X-Ray
Sample
D
ϕ = 0°
qy
b)
ϕ = 0°
qy [Å-1]
qx [Å-1]
c)
ϕ = 0°
qz [Å-1]
qx [Å-1]
d)
[100]Si | | [110]BTO
qx
S
X-Ray
Sample
D
ϕ = 45°
qy
e)
ϕ = 45°
qy [Å-1]
qx [Å-1]

f)

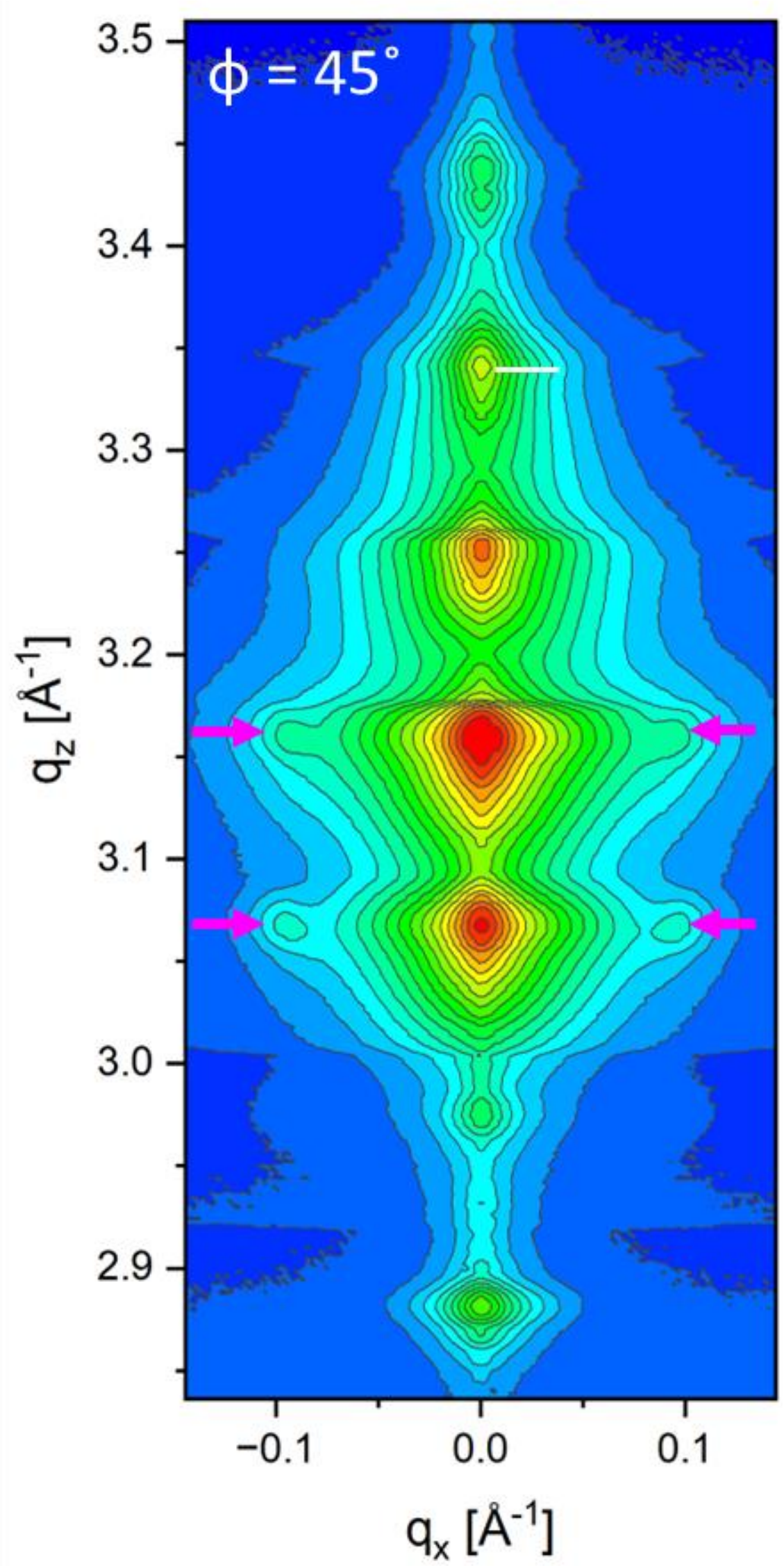

Figure S6: Synchrotron-radiation X-ray diffraction reciprocal space maps on a (12 u.c. $BaTiO_3$ / 5 u.c. $SrTiO_3$)$_{10}$ superlattice on Si (001). a) Schematic of the sample orientation with respect to the X-ray beam at ϕ = 0°. b, c) Reciprocal space maps recorded with ϕ = 0°, in the q_x-q_y plane for b) and q_x-q_z plane for c). d) Schematic of the sample orientation with respect to the X-ray beam at ϕ = 45°. e, f) Reciprocal space maps recorded with ϕ = 45° in the q_x-q_y plane for e) and in the q_x-q_z plane for f). Magenta arrows highlight the satellite peaks arising from the lateral polar order. Reciprocal space maps shown in the main text were recorded with ϕ = 45°.

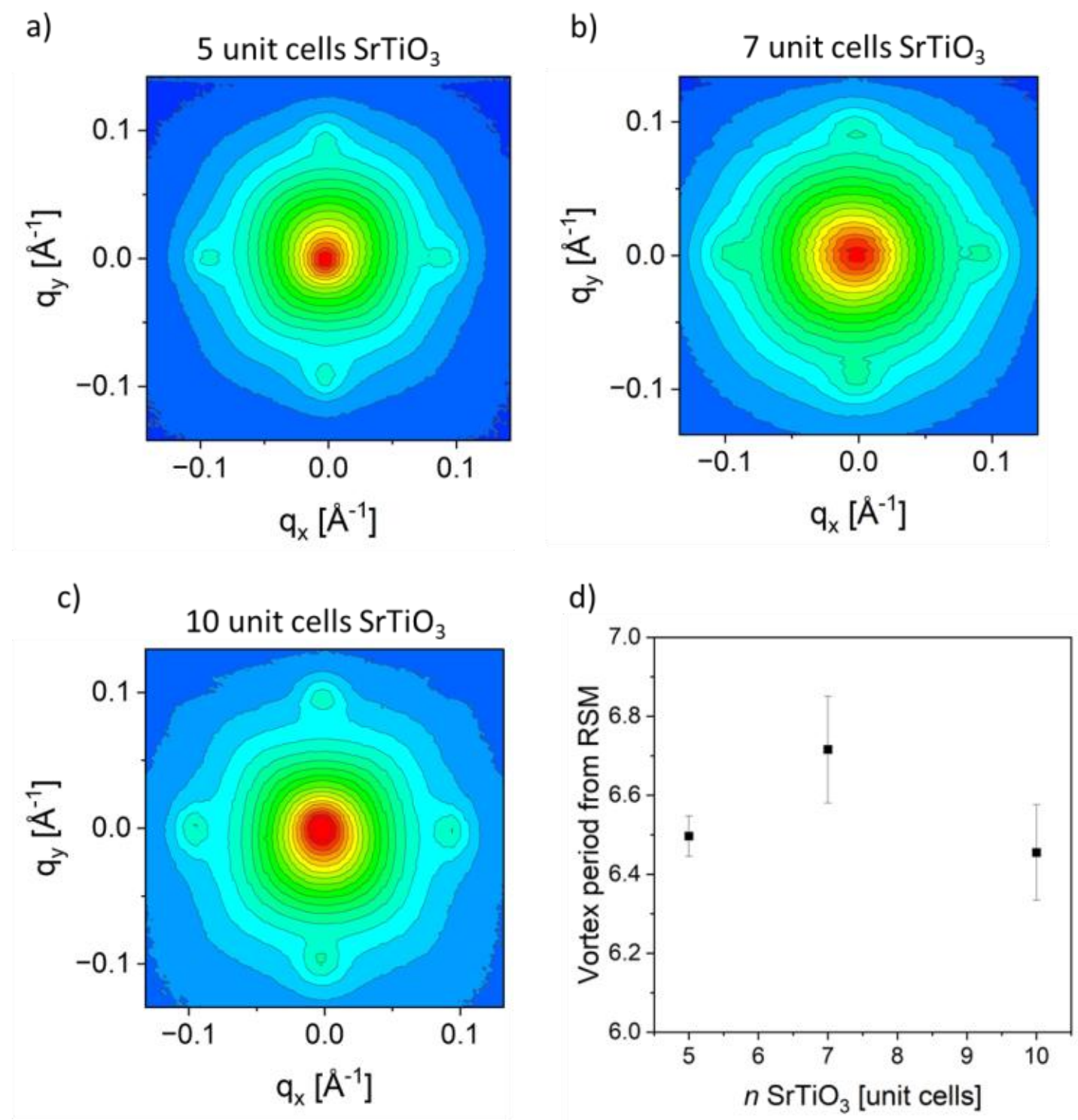

Figure S7: Reciprocal space maps of (12 u.c. $BaTiO_3$/n u.c. $SrTiO_3$)$_{10}$ superlattices on Si (001) around the 002 Bragg peak in q_x-q_y projection with the sample rotated at ϕ = 45° relative to the beam. a) n = 5 u.c., b) n = 7 u.c., and c) n = 12 u.c., d) Average periodicity of the polar domains (vortices) as a function of the number of unit cells in the $SrTiO_3$ interlayers, determined from the position of the satellite peaks.

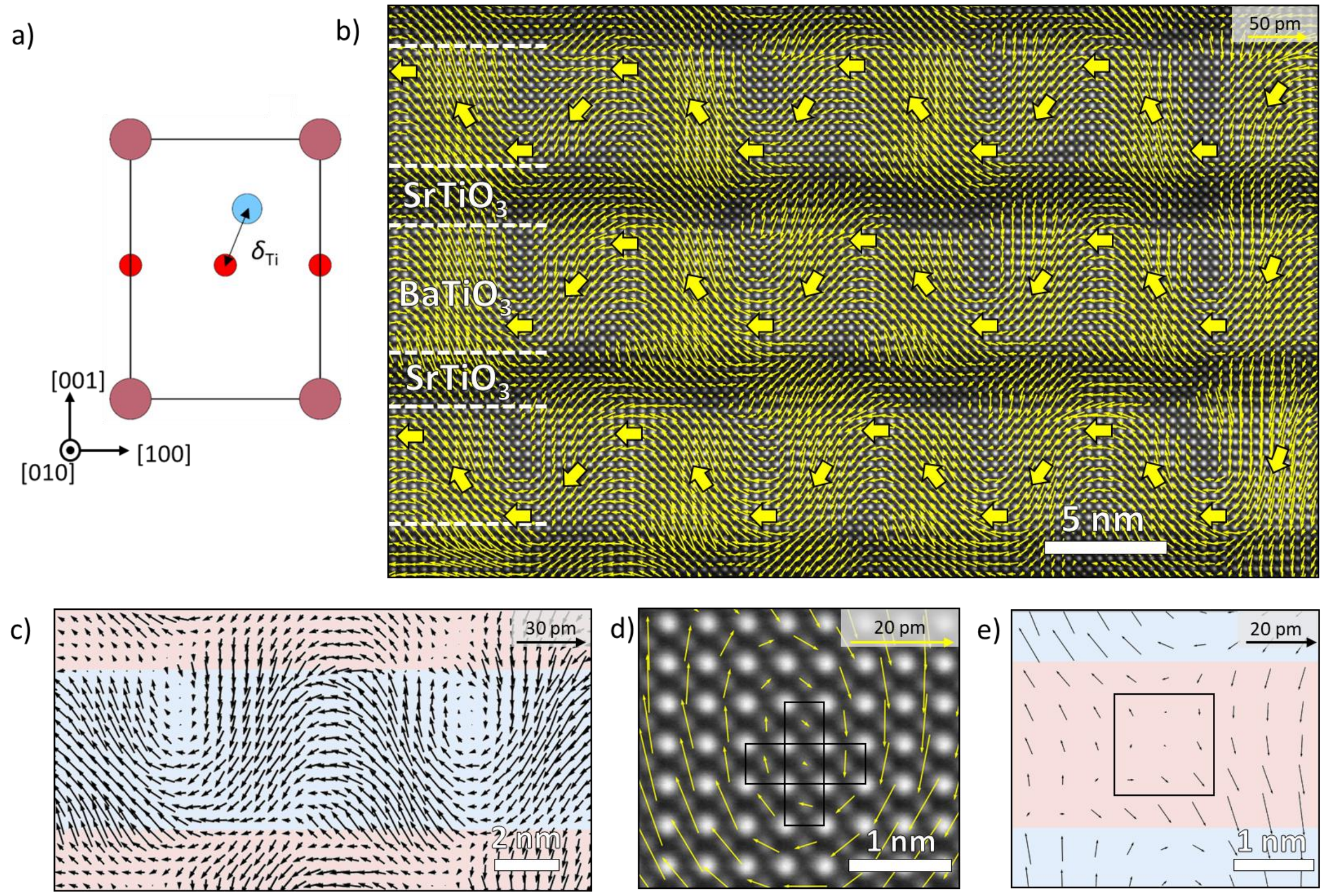

Figure S8: Ti-displacement vector maps from STEM images of a (12 u.c. $BaTiO_3$ / 5 u.c. $SrTiO_3$)$_{10}$ superlattice on Si (001) recorded with [010]$_{BaTiO3}$ zone axis. a) Projection of the $BaTiO_3$ unit cell in the (010) plane. b) Map of the Ti-displacement vectors determined from the superimposed HAADF-STEM image obtained on a [100] cut lamella c) Ti-displacement vector map showing a $BaTiO_3$ layer (blue) with adjacent $SrTiO_3$ layers (pink). A dipole wave is observed as well as vortices. d) Magnified atomic-resolution HAADF-STEM image overlaid with the corresponding Ti-displacement vector map showing a vortex. The vortex core is marked with black boxes e) Ti-displacement vector map showing an antivortex in a $SrTiO_3$ layer. The pink and blue color corresponds to $SrTiO_3$ and $BaTiO_3$, respectively. The black box indicates the core of the antivortex. The anti-vortices in $SrTiO_3$ are observed on top of vortices in $BaTiO_3$.

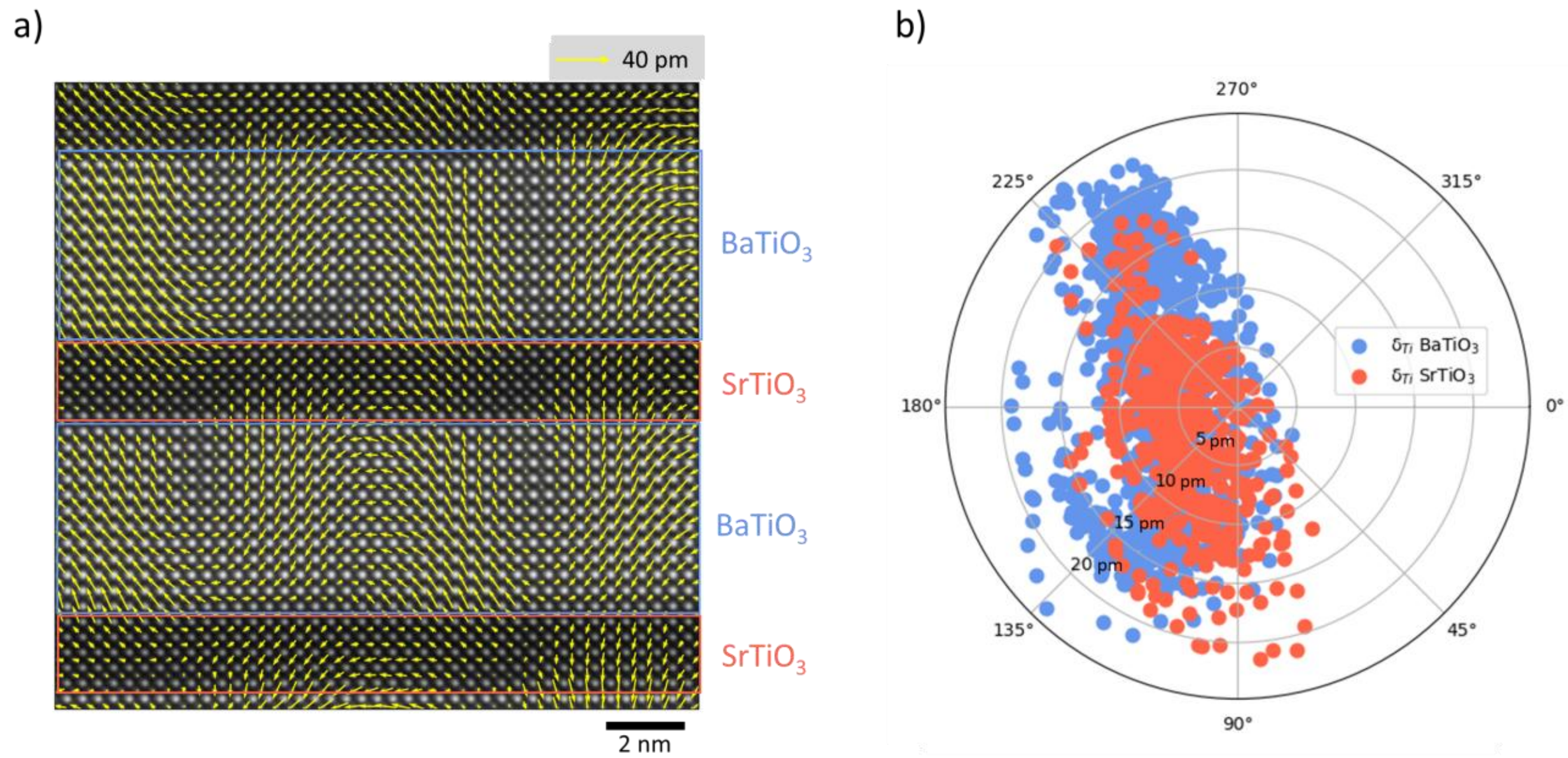

Figure S9: Analysis of the Ti displacement in a (12 u.c. $BaTiO_3$ / 5 u.c. $SrTiO_3$)$_{10}$ superlattice on Si (001). a) HAADF-STEM image in [100] zone axis overlaid with Ti-displacement vectors shown as yellow arrows. Blue and red boxes indicate the $BaTiO_3$ and $SrTiO_3$ layers. b) Polar plot of the direction and magnitude of the Ti-displacement vector extracted from the $BaTiO_3$ (two blue boxes in a)) and $SrTiO_3$ layers (two red boxes in a)). Each dot is representative of the Ti displacement in a unit cell in the STEM image shown in a).

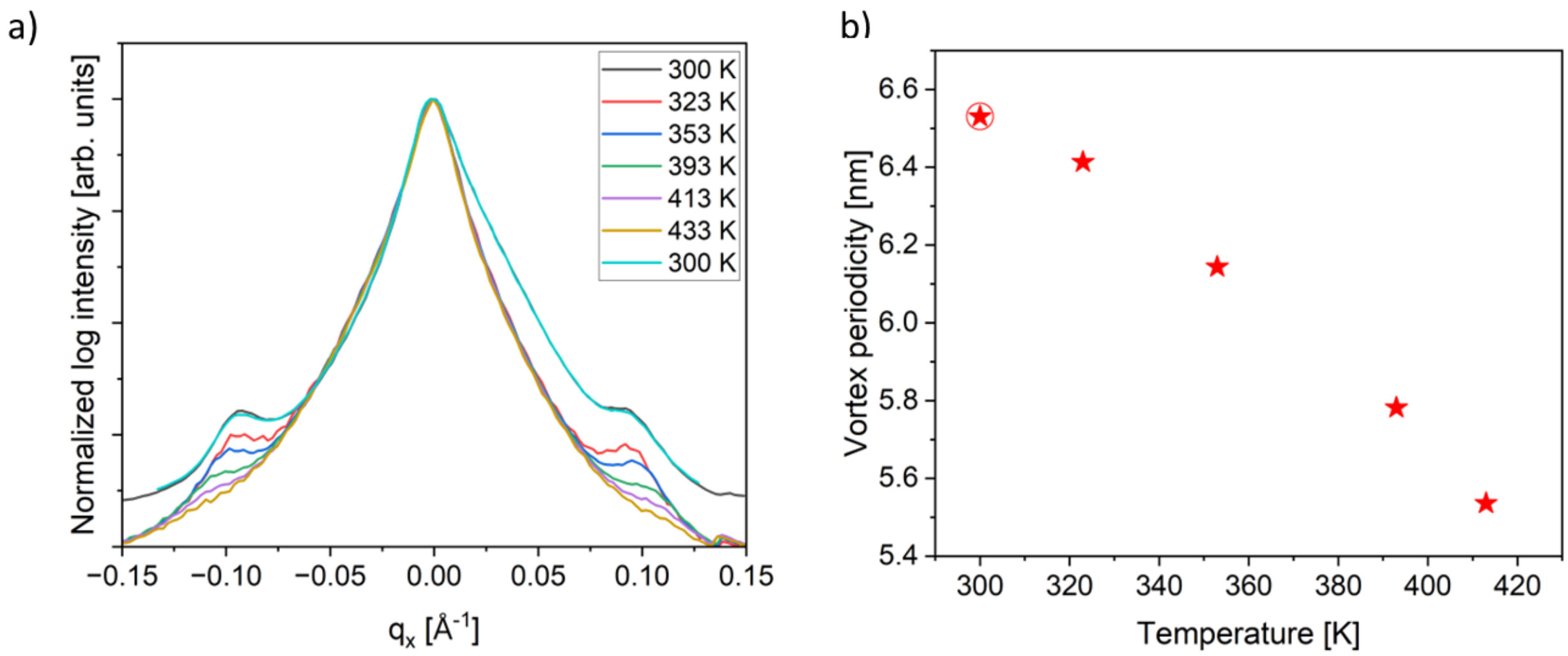

Figure S10: Temperature-dependent periodicity of the polar vortices in a (12 u.c. $BaTiO_3$/ 5 u.c. $SrTiO_3$)$_{10}$ superlattice on Si (001). a) Line profiles extracted from the RSMs recorded at different temperatures, shown in Figure 5 of the manuscript. The black and turquoise curves correspond to RSMs at 300 K recorded before and after heating, respectively. The measurements done upon heating in the range 323-433 K were performed using a dome around the sample (which explains the lower scattering background compared to room temperature). b) Vortex periodicity as a function of temperature.

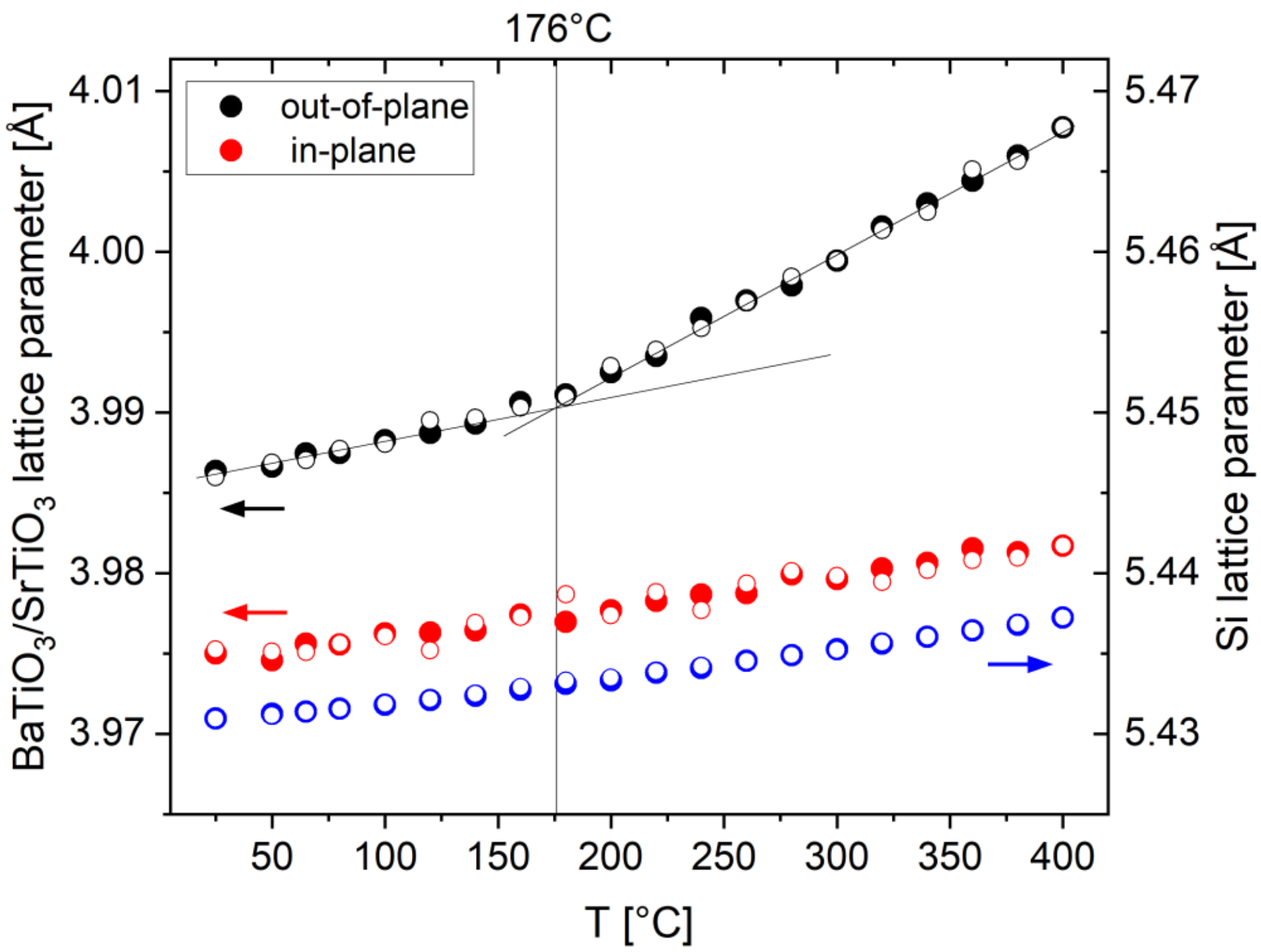

Figure S11: **Temperature-dependent lattice parameters of the (12 unit-cells $BaTiO_3$/ 5 unit-cells $SrTiO_3)_{10}$ superlattice on Si (001) and of the silicon substrate.** Full and open circles correspond to lattice parameters extracted during sample heating and cooling, respectively.

Note: the larger thermal expansion rate above the phase transition (here at ~ 176 °C) is consistent with reports for $BaTiO_3$ ceramics (Y. He, *Thermochim. Acta* **419**, 135 (2004)).

Quantitative comparison between experiment and simulation

Key parameters for the comparison between experiment and phase-field simulation are shown in Table S1.

Table S1: Comparison of key parameters from experiments and phase field simulations for an effective lattice parameter a_{eff} = 3.99 Å. The abbreviation oop stands for out-of-plane and ip for in-plane. The polarization ratios correspond to the Ti displacement ratios.

	**Experiment**	**Simulation**
In-plane lattice parameter [Å]	3.982	3.99
Vortex periodicity [nm]	6.5 (RSM) - 7.0 (plan view STEM)	7.24
$P_{oop}(BaTiO_3)/P_{ip}(BaTiO_3)$	8.65	5.68
$P_{oop}(SrTiO_3)/P_{ip}(SrTiO_3)$	1.78	0.55
$P_{oop}(BaTiO_3)/P_{oop}(SrTiO_3)$	2.61	5.43
$P_{ip}(BaTiO_3)/P_{ip}(SrTiO_3)$	0.54	0.53

Phase-field simulations performed with an effective substrate lattice parameter a_{eff} = 3.99 Å provide a good agreement with the experimental observations (including the periodic alignment along the <110> directions). Simulations with a_{eff} = 3.97 Å result in a more isotropic polarization pattern (Figure 7a). The experimentally determined average in-plane lattice parameter is of 3.982 Å (XRD), a value close to 3.99 Å. The discrepancy between the experimental (3.982 Å) and simulated (3.99 Å) lattice parameters likely arises from the simplified boundary conditions inherent to the phase-field model and from local strain inhomogeneities present in the real heterostructure.

Overall, the quantitative agreement in vortex periodicity and the relative polarization distribution between layers supports the predictive capability of the model. The vortex periodicity obtained from simulations (7.24 Å) shows a good agreement with experimental values extracted from STEM analysis (in the range 6.4 – 7.5 nm for different cross views and ~7.0 nm from planar view analysis).

Regarding the comparison of the relative polarization distributions, the experimental values are based on the relative Ti displacements within each unit cell determined from HAADF, which are proportional to the local polarization. This enables a consistent evaluation of polarization ratios between experiment and simulation. Overall, good agreement is found for the relative in-plane polarization between $BaTiO_3$ and $SrTiO_3$ layers. The ratios involving the out-of-plane components indicate that the simulations underestimate the magnitude of the induced out-of-plane polarization in the $SrTiO_3$ layers compared to experiment, while the polarization in $BaTiO_3$ is reproduced reasonably well.

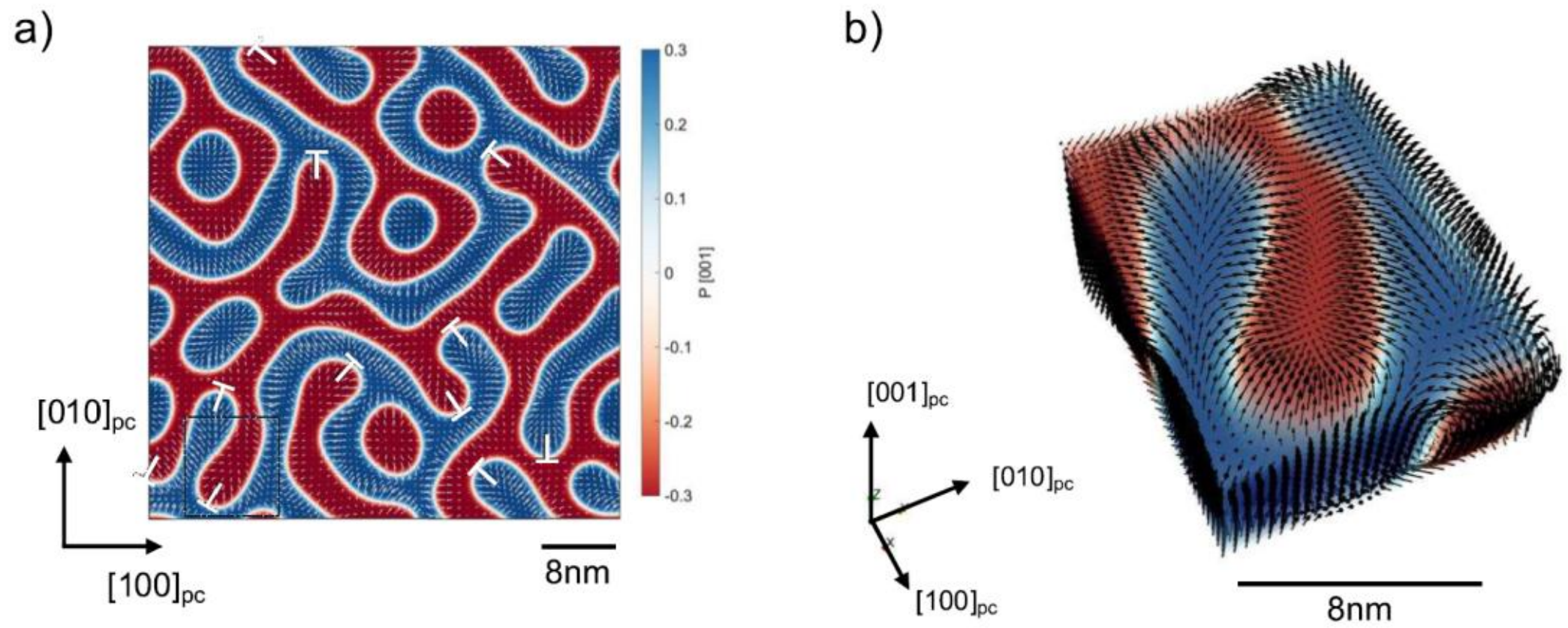

Figure S12: Phase field simulation of a (12 u.c. $BaTiO_3$/ 5 u.c. $SrTiO_3$)$_{10}$ superlattice on silicon Si (001). a) Slice projected onto (001) plane colored by the polarization along the [001] direction. Néel-type polar disclinations are highlighted. b) Magnified view of a representative Néel-type polar disclination.

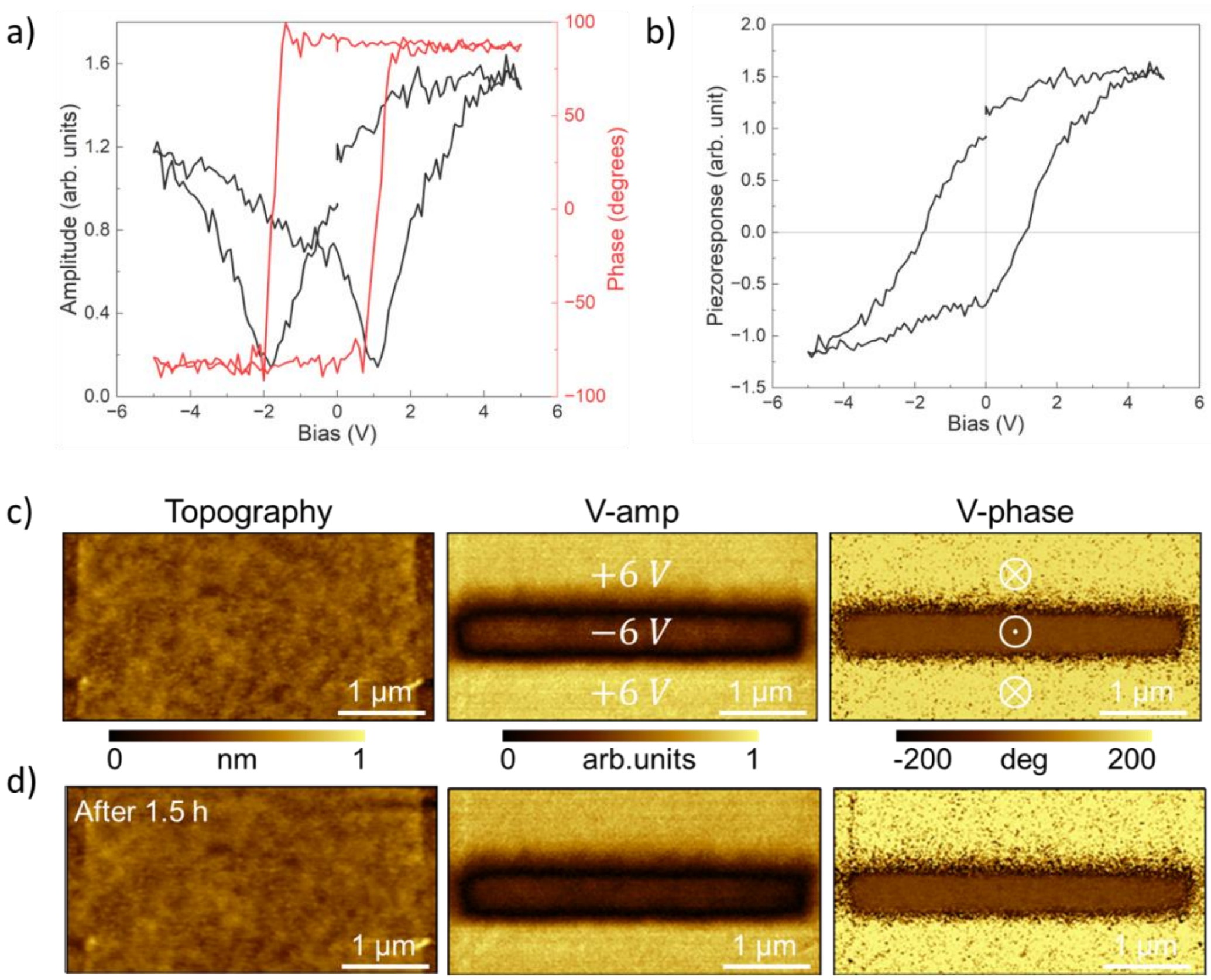

Figure S13: Piezoresponse force microscopy (PFM) on a (12 u.c. $BaTiO_3$/ 5 u.c. $SrTiO_3$)$_{10}$ superlattice on silicon Si (001). a) Switching spectroscopy PFM amplitude and phase signals, b) corresponding piezoloop c) Topography and PFM images (vertical amplitude and phase) of electrically poled regions (poling using ± 6 V DC). d) Same imaging after 1.5 hours. Imaging of the poled domains was done using an ac reading bias of 0.5 V. The black spots in the V-phase images observed in the bright regions (poled down) are due to phase wrapping (as determined from the phase signals on these locations).

We observe a significant electrostatic charging in the amplitude signal, which could be due to the presence of the top $SrTiO_3$ film.

Parameters in the thermodynamic free-energy function

For $BaTiO_3$, we use the cubic phase $(m3m)$ as our high symmetry reference state and employ an 8th-order landau expansion describe the relative stability of the lattice polarization compared to the cubic reference state. The coefficients used for this paper are from Li et. al[3] and adapted from Li et al[4].

Table S2. Coefficients in the thermodynamic free energy function for $BaTiO_3$ and $SrTiO_3$

Coefficient	$BaTiO_3$	$SrTiO_3$
$a_{11}\left(\frac{J}{m^3}\ \frac{m^4}{C^2}\right)$	$a_0(T-T_c)$	$a_0\ 54\left(\coth\left(\frac{54K}{T}\right)-\coth(\frac{54K}{T_c})\right)$
T_c (K)	388	30
$a_0\left(\frac{J}{m^3}\ \frac{m^4}{K\,C^2}\right)$	4.124×10^5	7.5×10^5
$a_{1111}\left(\frac{J}{m^3}\ \frac{m^8}{C^4}\right)$	-2.097×10^8	1.7×10^9
$a_{1122}\left(\frac{J}{m^3}\ \frac{m^8}{C^4}\right)$	7.974×10^8	1.37×10^9
$a_{111111}\left(\frac{J}{m^3}\ \frac{m^{12}}{C^6}\right)$	1.294×10^9	0
$a_{111122}\left(\frac{J}{m^3}\ \frac{m^{12}}{C^6}\right)$	-1.95×10^9	0
$a_{112233}\left(\frac{J}{m^3}\ \frac{m^{12}}{C^6}\right)$	-2.509×10^9	0
$a_{11111111}\left(\frac{J}{m^3}\ \frac{m^{16}}{C^8}\right)$	3.863×10^{10}	0
$a_{11111122}\left(\frac{J}{m^3}\ \frac{m^{16}}{C^8}\right)$	2.529×10^{10}	0
$a_{11112222}\left(\frac{J}{m^3}\ \frac{m^{16}}{C^8}\right)$	1.637×10^{10}	0
$a_{11112233}\left(\frac{J}{m^3}\ \frac{m^{16}}{C^8}\right)$	2.529×10^{10}	0
Q_{11} (m^4/C^2)	0.10	0.0509

Q_{12} (m^4/C^2)	-0.034	-0.0135
Q_{44} (m^4/C^2)	0.029	0.00957
$C_{11}(Pa)$	1.78×10^{11}	$1.78 \times 10^{11}*$
C_{12} (Pa)	0.964×10^{11}	$0.964 \times 10^{11}*$
C_{44} (Pa)	1.22×10^{11}	$1.22 \times 10^{11}*$

*The simulations assume the elastic stiffness tensor is uniform throughout the film, therefore, $SrTiO_3$ possess the same elastic stiffness as $BaTiO_3$ in these simulations

References

1. Demkov, A. A. & Posadas, A. B. *Integration of Functional Oxides with Semiconductors*. vol. 25 (Springer, 2014).
2. Först, C. J., Ashman, C. R., Schwarz, K. & Blöchl, P. E. The interface between silicon and a high-k oxide. *Nature* **427**, 53 (2004).
3. Li, Y. L., Cross, L. E. & Chen, L. Q. A phenomenological thermodynamic potential for $BaTiO_3$ single crystals. *J. Appl. Phys.* **98**, (2005).
4. Li, Y. L. *et al.* Phase transitions and domain structures in strained pseudocubic (100) $SrTiO_3$ thin films. *Phys. Rev. B* **73**, 184112 (2006).
