# Supplementary material for: Periodic polar vortices in BaTiO3/SrTiO3 superlattices monolithically grown on silicon
Source: Nat Commun. 2026 Sep 8;17:9648. doi: 10.1038/s41467-026-77270-0 (PMC13558690; doi:10.1038/s41467-026-77270-0)
Supplement: Supplementary file 2 [file 41467_2026_77270_MOESM2_ESM.pdf]

nature portfolio

Peer Review File

Periodic polar vortices in $BaTiO_3/SrTiO_3$ superlattices monolithically grown on silicon

Corresponding Author: Professor Catherine Dubourdieu

This file contains all reviewer reports in order by version, followed by all author rebuttals in order by version.

Version 0:

Reviewer comments:

Reviewer #1

(Remarks to the Author)
The authors have reported the observation of vortices and polar waves in BTO/STO superlattices grown on Si, a notably valuable finding that advances the feasibility of high-density, lead-free CMOS-compatible devices. Below are some suggestions and questions:
1.Could the authors elaborate on the role of dislocations in the formation of the observed polar patterns? Specifically, might dislocations have an impact on the periodicity of the polar vortices or waves?
2.The STO layer is proposed to act as a mediator for vortex formation in BTO, leading to egg-like (rather than circular) polar patterns. Whether there is a correlation between the superlattice periodicity (i.e., the thicknesses of BTO/STO layers) and the shape/density of the vortices.
3.As shown in Figure S6, some regions exhibit polar waves or a combination of polar waves and vortices, while others form closed vortices. Could the authors shed light on the triggering conditions that lead to the formation of polar waves and closed vortices? Additionally, regarding Figure 6b, it would be helpful to explain why each slice of the BTO/STO superlattice displays distinct polarization states rather than continuous patterns.
4.The authors have conducted macroscopic studies on polar topologies under temperature cycling and predicted the phase transition of BTO at low temperatures. Given that BTO's Curie temperature (Tc) is as high as 420 K, it would be valuable to explore whether there are any microscopic changes (e.g., the emergence, evolution, or diminishment of vortices, polar waves, or skyrmions) during heating or cooling processes above room temperature.
5.If it is possible, could the authors further discuss whether the vortices/polar waves are switchable under external stimuli (e.g., electric fields)? Given the distinct polarization states of individual BTO layers, it would also be interesting to clarify if such switching is controllable and reversible. Additionally, while phase field simulations have been used to illustrate the polarization distribution of the polar patterns, supplementing with nanoscale characterization of polar domains (e.g., piezoresponse force microscopy, PFM) would significantly strengthen the findings.
6.Some minor issues should be improved. i.e., no Figure 2f in the manuscript (page 6, line 157); not all supplementary materials are explicitly referenced in the main text.

Reviewer #2

(Remarks to the Author)
This work provided a comprehensive study of periodic polar vortices in BaTiO3/SrTiO3 superlattices epitaxially integrated onto silicon substrates, employing a synergistic combination of temperature-dependent XRD and atomic-resolution STEM to characterize the crystallographic symmetry and the long-range ordering of these topological textures. The experimental observations are further supported by phase-field simulations, which elucidate the critical role of substrate-induced tensile strain and interlayer coupling in stabilizing the vortex tubes. The manuscript is well-organized with high-quality data. However, there are a few issues need to be clarified before the manuscript can be considered for publication.

1. In Figure 1a, the author attributes the observed dislocations to thermal expansion mismatch. Since topological textures are extremely sensitive to local strain gradients, it is essential to discuss the spatial correlation between these dislocations and vortex. Do these dislocations act as pinning sites that stabilize the periodicity, or serve as sources of disorder that disrupt the long-range coherence of vortices?
2. Noticeable discrepancy between the average periodicity measured by STEM (~7.5 nm) and XRD (~6.5 nm): while local structural variations often differ from macroscopic averages, a 15% difference is quite substantial for a long-range ordered

system. The authors should confirm the periodicity by statistically analyzing larger areas of STEM images, or discuss whether this discrepancy indicates slight inhomogeneities in the superlattice growth.
3. The author demonstrates the stability of the vortex state up to 450 K. Considering the significant thermal expansion mismatch, does the vortex periodicity exhibit hysteretic behavior during heating-cooling cycles?
4. On Page 9, the author mentioned that the TC (~440 K) of superlattices is higher than that of bulk materials (~393 K). For thin layers (< 5 nm), the strong depolarization field typically leads to a sharp reduction in TC due to the finite-size effect. While the authors emphasize the stability of the residual tensile strain from the silicon substrate, it is necessary to clarify how the strain-induced enhancement compensates for and eventually exceeds the reduction in TC?
5. As the authors stated, topological polar textures have been reported frequently. These patterns are scientifically interesting since they differ from typical ferroelectric domain patterns. However, for people new to this area, it is not clearly introduced how these exotic polar patterns can be used in potential applications. I believe some introduction to the potential use will appeal to a broader readership of this manuscript.

Reviewer #3

(Remarks to the Author)
The work being carried out by the author is meaningful and it is hoped that the following issues can be explained.
1. The conclusion part of the paper emphasizes the significance of realizing topological structures on silicon for "topotronic devices", but it is too broad. It is suggested to add a dedicated paragraph that combines the negative capacitance effect of ferroelectric materials, controllable ionic transport, and interface charge modulation, and specifically discusses its potential applications in new resistive memories, solid-state battery interface layers, and electrocatalytic carriers in energy information devices. This will greatly enhance the interdisciplinary influence of the paper.
2. As the active layer that may be used in the device, the $BaTiO_3/SrTiO_3$ superlattice's stability in an electrochemical environment is the key to its practical application. The author has overlooked the preliminary assessment of electrochemical stability. It is suggested to mention this issue in the discussion section, or to indicate that this is a direction that must be studied before integrating it into energy devices such as solid-state batteries.
3. The paper repeatedly mentions CMOS compatibility, but does not deeply discuss the challenges that this topological structure may face when integrated with existing silicon-based chip processes (such as thermal budget, etching, electrode materials). It is suggested to add relevant discussions.
4. The periodic vortex array and strain coupling observed in the paper may generate enhanced or novel piezoelectric/thermoelectric responses? The piezoelectric/thermoelectric effect of ferroelectric materials is the key to energy harvesting. The author is requested to explain this.
5. In electrochemical devices, ion migration will greatly affect performance. For this topological structure with a strong polarization gradient and complex built-in electric field, will it affect or regulate the distribution and migration of oxygen vacancies and other defects?
6. The author indirectly proved the polarization vortex through displacement mapping, but lacks measurement of the overall macroscopic ferroelectric properties of the superlattice (such as the P-E hysteresis loop, leakage current characteristics). This is not conducive to evaluating its quality as a ferroelectric functional layer, and lacks direct measurement of key ferroelectric properties, so the author is requested to make a supplement.
7. Figures 3 and the text mention the correlation between strain and vortices, but the spatial distribution statistics of the strain quantitative values (such as histograms) are missing. In addition, thermal mismatch strain is the core mechanism, but if the strain can be tracked more directly with temperature changes through variable-temperature XRD or Raman, it will be more persuasive.
8. The paper observed that the vortices are in the ground state and thermally stable, but did not explore their dynamic behavior (such as writing, erasing, flipping) under an applied electric field. The author is requested to explain this.
9. The paper states that the $SrTiO_3$ layer is polarized at the interface, which is the key to coupling. It is suggested to conduct a more quantitative analysis of the size and depth distribution of the polarization within the $SrTiO_3$ layer.
10. It is recommended to consider using EELS or EDS line scans to verify the sharpness of Ba and Sr elements at the interface, excluding inter-diffusion, which can further support the "sharp interface" conclusion and relate it to the uniformity of performance.
11. The simulation in Figure 6 qualitatively agrees with the experiment, but lacks quantitative comparison of key parameters, such as vortex period, absolute value of polarization strength, and matching degree of strain size. Enhancing quantitative comparison can greatly enhance the credibility and predictive power of the model. The author is requested to make a supplement.
12. The paper assumes that the vortex tube is infinitely extended. In actual finite-sized devices or regions with dislocations (such as Figure 1a), how does the vortex tube terminate? Will it form topological defects (such as Bloch points)? This is important for understanding the non-uniformity of device micro-region performance, and it is recommended to make a brief discussion.
13. Some pictures in the paper are not easy to understand, for example: Figure 1e: the intensity of the satellite peak is weak. It is suggested to add arrows or clear labels in the figure, or provide an enhanced contrast version in the supplementary figure.
14. Figure 5a: multiple RSM diagrams are densely arranged, and the satellite peaks (marked *) in the line spectrum are not obvious. It is recommended to consider re-arranging or displaying the key temperature RSMs in a larger size separately. 15. Figure 7: The dimensions of subgraphs (a) and (b) are too small, making it difficult to clearly observe the details of the structural evolution. It is recommended to enlarge them or provide enlarged images of the key areas.
16. The "Methods" section can be more concise and focus on the key points: The description of the FIB preparation process is overly detailed (occupying nearly two pages), and it repeats similar steps for different devices. It is recommended to significantly simplify it, merge the common steps, and use the saved space for in-depth discussion sections.

Reviewer #4

(Remarks to the Author)
I co-reviewed this manuscript with one of the reviewers who provided the listed reports. This is part of the Nature Communications initiative to facilitate training in peer review and to provide appropriate recognition for Early Career Researchers who co-review manuscripts.

Version 1:

Reviewer comments:

Reviewer #1

(Remarks to the Author)
All my concerns have been addressed in the revisions.

Reviewer #2

(Remarks to the Author)
The authors have carefully addressed the comments and resolved several issues. However, a few statements remain insufficiently supported or internally inconsistent and should be revised.

1. The authors describe the vortex periodicity as " reversible and non-hysteretic" with temperature. The return of the satellite peak position to its original position after cooling supports this reversibility, but the authors claim they cannot provide complete satellite peak measurements for the entire cooling process. A system may exhibit substantial hysteresis during heating and cooling while still recovering its initial room-temperature configuration. The author should revise the relevant descriptions.

2. The PFM results do not provide sufficient evidence that the vortices are unwound under an applied bias. PFM resolution (~20 nm) is much larger than the vortex diameter (~3.5 nm), and the authors also report significant electrostatic charging and strong leakage. Therefore, the butterfly shape in the amplitude signal, 180° phase change, and stable written contrast may include electrostatic, charge-injection, ionic, electrochemical, and piezoelectric contributions. The ±6 V written regions only demonstrate a persistent bias-induced contrast, not vortex unwinding. The statement “it is possible to unwind vortices” should therefore be revised.

3. Several inconsistencies between the manuscript and the response letter should be verified. The patterned top electrode is described as Pt in one place and W in another. In addition, the corrected in-plane lattice parameter is reported as 3.980 Å in the response letter but as 3.982 Å in the revised manuscript.

Reviewer #4

(Remarks to the Author)
I co-reviewed this manuscript with one of the reviewers who provided the listed reports. This is part of the Nature Communications initiative to facilitate training in peer review and to provide appropriate recognition for Early Career Researchers who co-review manuscripts.

Version 2:

Reviewer comments:

Reviewer #2

(Remarks to the Author)
The authors have carefully addressed the comments, and all my concerns have been addressed in the revisions.

Reviewer #4

(Remarks to the Author)
I co-reviewed this manuscript with one of the reviewers who provided the listed reports. This is part of the Nature Communications initiative to facilitate training in peer review and to provide appropriate recognition for Early Career Researchers who co-review manuscripts.

Open Access This Peer Review File is licensed under a Creative Commons Attribution 4.0 International License, which permits use, sharing, adaptation, distribution and reproduction in any medium or format, as long as you give appropriate credit to the original author(s) and the source, provide a link to the Creative Commons license, and indicate if changes were made.
In cases where reviewers are anonymous, credit should be given to 'Anonymous Referee' and the source.
The images or other third party material in this Peer Review File are included in the article's Creative Commons license, unless indicated otherwise in a credit line to the material. If material is not included in the article's Creative Commons license and your intended use is not permitted by statutory regulation or exceeds the permitted use, you will need to obtain permission directly from the copyright holder.
To view a copy of this license, visit https://creativecommons.org/licenses/by/4.0/

REVIEWER COMMENTS

We thank the reviewers for their thorough reviews, questions and comments. We appreciate their constructive criticism and suggestions. We have addressed the comments raised and performed additional experiments.
We have treated numerous STEM images on the original sample for statistics. We have performed additional measurements on this system: new lamellae preparation for STEM, electron energy loss spectroscopy in a STEM, energy dispersive X-ray spectrometry in a STEM, piezoresponse force microscopy, and temperature-dependent X-ray diffraction for the determination of the change in lattice parameters with temperature (both in-plane and out-of-plane). We also prepared new superlattice samples and characterize them (synchrotron and laboratory XRD, STEM). Finally, complementary phase field modeling was performed. The significant number of additional experiments took several months.

The additional EELS, EDX, STEM experiments involved two collaborators - Dr. Minh Anh LUONG at HZB and Dr. Benedikt HAAS at the Humboldt University Berlin where the Nion microscope is located - who have been added to the author's list (Dr. Ines Häusler, our former microscopist expert, is no more working at HZB).

Reviewer #1

Remarks to the Author: The authors have reported the observation of vortices and polar waves in BTO/STO superlattices grown on Si, a notably valuable finding that advances the feasibility of high-density, lead-free CMOS-compatible devices. Below are some suggestions and questions:

Comment 1: Could the authors elaborate on the role of dislocations in the formation of the observed polar patterns? Specifically, might dislocations have an impact on the periodicity of the polar vortices or waves?

Response: The periodic vortex tubes are locally disrupted by dislocation defects. In the plan-view HAADF-STEM data (Figure R1 below which is a zoom on Figure 4.a in the manuscript), the dislocation defects appear as regions of dark contrast. The periodic stripe pattern terminates or locally loses coherence in the regions where dislocations are present, indicating that these defects disturb the long-range lateral periodicity of the polar vortices.

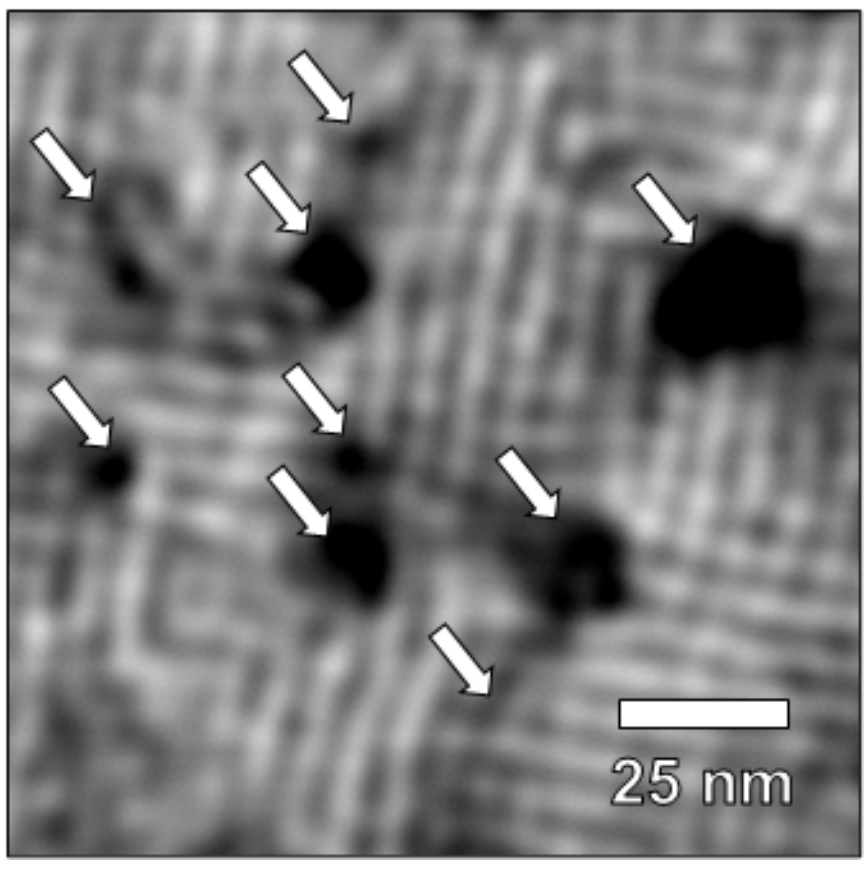

Figure R1: Plan-view STEM image of the $BaTiO_3$-$SrTiO_3$ superlattice containing dislocations. Regions of dark contrast marked by arrows are dislocations defects.

Strong and highly inhomogeneous strain fields around dislocations perturb the atomic columns, preventing an unambiguous determination of atomic positions and hindering the extraction of Ti-displacement vectors. Also, these dislocations alter the coupled electrostatic and elastic interactions that lead to the intrinsic polar ordering in the $BaTiO_3$/$SrTiO_3$ superlattices. As a result, the polar textures cannot be resolved in cross-sectional STEM near dislocations.

Modification to the manuscript:

- p.7, line 179: Due to the strong and highly inhomogeneous strain fields around dislocations, the atomic positions and therefore the Ti-displacement vectors can not be precisely determined in their vicinity. Hence, we can not determine whether the dislocations have an impact on the periodicity of the polar patterns.

- p.10, line 239: The periodic vortex tubes are disrupted by dislocation defects which appear as regions of dark contrast in Figure 4.a. The periodic stripe pattern terminates or locally loses coherence in the regions where dislocations are present, indicating that these defects disturb the long-range lateral ordering of the polar vortices.

Comment 2: The STO layer is proposed to act as a mediator for vortex formation in BTO, leading to egg-like (rather than circular) polar patterns. Whether there is a correlation between the superlattice periodicity (i.e., the thicknesses of BTO/STO layers) and the shape/density of the vortices.

Response: To investigate the influence of superlattice periodicity on the polar vortices, we have studied two additional (12u.c. $BaTiO_3$/n u.c. $SrTiO_3$)$_{10}$ superlattices, with n = 7 u.c. and 10 u.c.. The periodicity of the vortices was determined from the position of the in-plane satellite peaks in the reciprocal space maps performed at DESY synchrotron (shown in Figure R2.b,c below together with the RSM from the 12 u.c. / 5 u.c. system (Fig. R2.a) already shown in the manuscript). For each system, the RSMs were performed on three different pieces per sample (each sample is 2" in size) and calculated the average vortex periodicity. The vortex periodicities are 6.50 ±0.05 nm, 6.72 ±0.14 nm and 6.46 ±0.12 nm for superlattices with 5 u.c., 7 u.c. and 10 u.c. of $SrTiO_3$, respectively. These results show that there is no significant change in the periodicity or density of the polar vortices in the investigated range of $SrTiO_3$ thicknesses (2.0 – 4.0 nm).

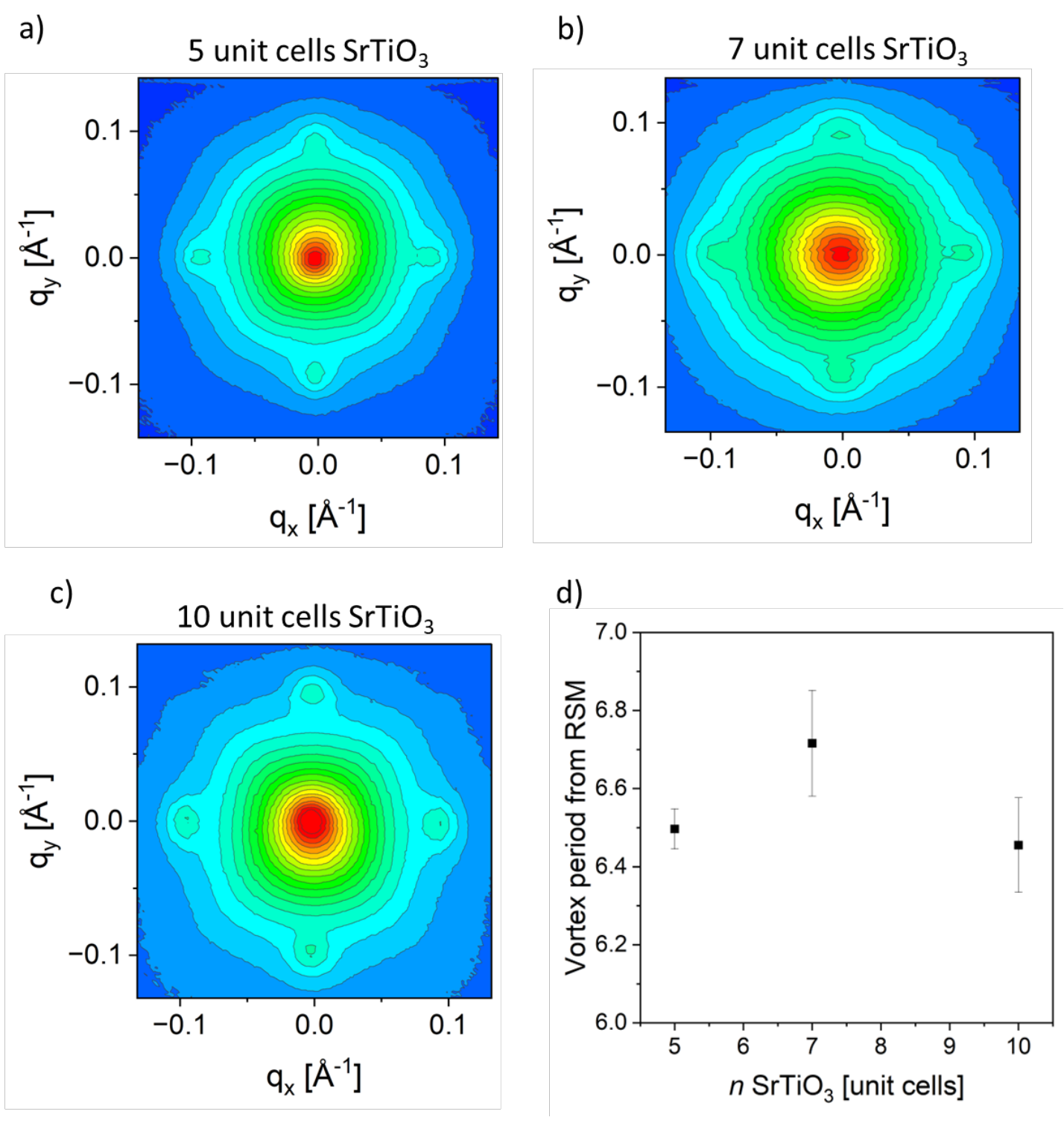

Figure R2: Reciprocal space maps of (12 u.c. $BaTiO_3$/n u.c. $SrTiO_3$)$_{10}$ superlattices around the 002 Bragg peak in q_x-q_y projection with the sample rotated at ϕ = 45° relative to the beam. a) n = 5 u.c., b) n = 7 u.c.,

and c) n = 12 u.c., d) Average periodicity of the polar domains (vortices) as a function of the number of unit cells in the $SrTiO_3$ interlayers, determined from the position of the satellite peaks.

We cannot comment on the shape of the polar vortices as a function of the $SrTiO_3$ interlayer thickness, as a systematic STEM study is highly time-consuming and beyond the scope of this work. Note that the polar displacements in $BaTiO_3$ are much smaller than in $PbTiO_3$, which is why there are only very rare studies on $BaTiO_3$ polarization mapping. To determine the polar pattern in the system under study in the manuscript, both in [100] and [110] lamellae were investigated; to have enough statistics for reliable conclusions, we had more than ten sessions (of one week each) on the Nion microscope (with a session every two months).

Modification to the manuscript:

- p.7, line 165: "The study of two other superlattices, (12 u.c. $BaTiO_3$ / n u.c. $SrTiO_3$)$_{10}$ with n = 7 u.c. (~ 2.8 nm) and 10 u.c. (~ 4.0 nm), shows the presence of similar lateral satellites corresponding to periodicities of 6.7 ±0.1 nm and 6.5 ±0.1 nm, respectively (Figure S7 of the Supporting Information). There is no significant change in the periodicity or density of the polar vortices in the investigated range of $SrTiO_3$ thicknesses (2.0 – 4.0 nm)."

Modification to the Supporting Information file:

- p. 10: We have added Figure S7.

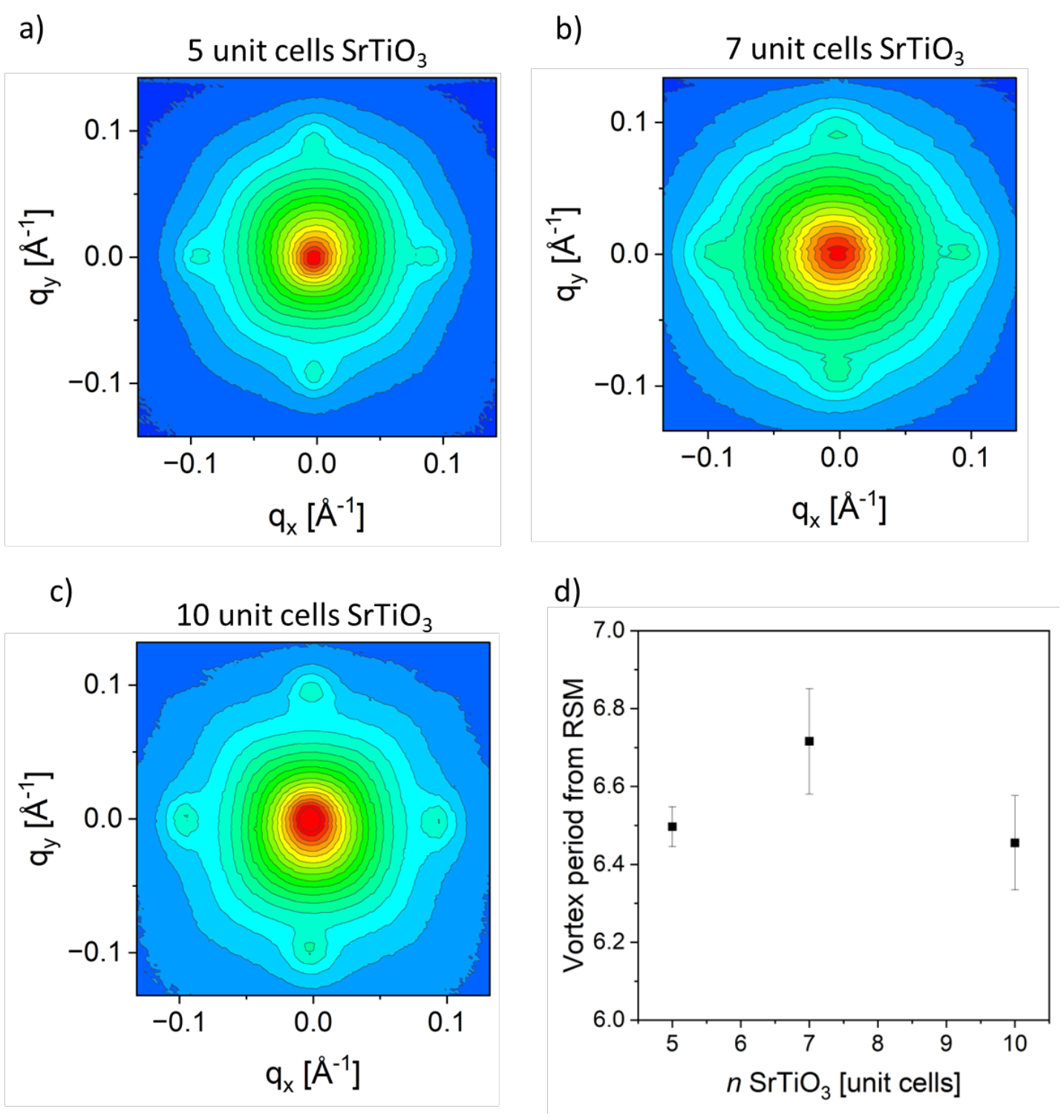

Figure S7: Reciprocal space maps of (12 u.c. $BaTiO_3$/n u.c. $SrTiO_3$)$_{10}$ superlattices around the 002 Bragg peak in q_x-q_y projection with the sample rotated at ϕ = 45° relative to the beam. a) n = 5 u.c., b) n = 7 u.c., and c) n = 12 u.c., d) Average periodicity of the polar domains (vortices) as a function of the number of unit cells in the $SrTiO_3$ interlayers, determined from the position of the satellite peaks.

Comment 3: As shown in Figure S6, some regions exhibit polar waves or a combination of polar waves and vortices, while others form closed vortices. Could the authors shed light on the triggering conditions that lead to the formation of polar waves and closed vortices?

Response: We thank the reviewer for raising this question. The stability of polar waves versus polar vortices is largely governed by the competition between electrostatic and gradient energy (Gong *et al.*, *Sci. Adv.* **7**, eabg5503 (2021); Tong *et al.*, *Nat. Phys.* **21**, 464-470 (2025)). The electrostatic energy promotes the stability of vortices over the polar waves as they minimize the polar discontinuity at the $BaTiO_3/SrTiO_3$ interface, while the gradient energy penalty destabilizes the polar vortices and promotes the dipolar wave since the gradient of the polarization is more gradual in the dipolar wave structure. As the magnitude of the spontaneous polarization decreases, the electrostatic energy penalty will decrease, thus stabilizing the polar wave, which is supported by the observation that the polar wave is stabilized at higher temperatures (Tong *et al.*, *Nat. Phys.* **21**, 464-470 (2025)). In conjunction with electrostatic considerations, local strain and local strain inhomogeneities play an important role for the appearance of polar vortices or polar waves. Phase diagrams obtained from phase-field simulations for the $PbTiO_3/SrTiO_3$ superlattices show that polar waves are stabilized under higher tensile strain and that under certain strain conditions both polar waves and vortices appear (Gong *et al.*, *Sci. Adv.* 7, eabg5503 (2021)). In our superlattice, dislocation defects introduce strongly inhomogeneous strain fields that locally modify the elastic energy landscape. Such strain gradients could locally favor specific polarization orientations.

Additionally, regarding Figure 6b, it would be helpful to explain why each slice of the BTO/STO superlattice displays distinct polarization states rather than continuous patterns.

Response: The appearance of discrete polarization states, rather than a continuous vortex texture, arises from the fact that the polar vortices are ordered along two orthogonal directions. In each 2D slice, the cutting plane intersects some vortex cores perpendicular to their axis while remaining parallel to others. As a result, vortices whose cores lie parallel to the slicing plane do not appear as circulating polarization patterns in that projection, leading to regions that exhibit apparently uniform polarization rather than vortex-like features.

Modifications to the manuscript:

- p. 7, line 185: "The stability of polar waves versus polar vortices is largely governed by the competition between electrostatic and gradient energy[22,41]. The electrostatic energy promotes the stability of vortices over the polar waves as they minimize the polar discontinuity at the $BaTiO_3/SrTiO_3$ interface, while the gradient energy penalty destabilizes the polar vortices and promotes the dipolar wave since the gradient of the polarization is more gradual in the dipolar wave structure. As the magnitude of the spontaneous polarization decreases, the electrostatic energy penalty will decrease, thus stabilizing the polar wave, which is supported by the observation that the polar wave is stabilized at higher temperatures[41]. In addition to electrostatic considerations, local strain inhomogeneities may play an important role for the appearance of polar vortices or polar waves. Phase diagrams obtained from phase-field simulations for the $PbTiO_3/SrTiO_3$ superlattices show that polar waves are stabilized under higher tensile strain and that under certain strain conditions both polar waves and vortices appear[22]. In our superlattice, dislocation defects introduce strongly inhomogeneous strain fields that locally modify the elastic energy landscape. Such strain gradients could locally favor specific polarization orientations."

- p.12, line 311:" Indeed, the appearance of discrete polarization states, rather than a continuous vortex texture, arises from the fact that the polar vortices are ordered along two orthogonal directions. In each 2D slice, the cutting plane intersects some vortex cores perpendicular to their axis while remaining parallel to others. As a result, vortices whose cores lie parallel to the slicing plane do not appear as circulating polarization patterns in that projection, leading to regions that exhibit apparently uniform polarization rather than vortex-like features."

Comment 4: The authors have conducted macroscopic studies on polar topologies under temperature cycling and predicted the phase transition of BTO at low temperatures. Given that BTO's Curie temperature (Tc) is as high as 420 K, it would be valuable to explore whether there are any microscopic changes (e.g., the emergence, evolution, or diminishment of vortices, polar waves, or skyrmions) during heating or cooling processes above room temperature.

Response: A systematic temperature-dependent STEM investigation, which would be required to directly track microscopic changes in the polar texture during heating or cooling, represents a substantial study and is beyond the scope of the present work. Note that the microscopic evolution of polar patterns with temperature has only recently been reported for $PbTiO_3/SrTiO_3$ superlattices (Tong *et al.*, *Nat. Phys.* **21**, 464–470 (2025)).

We have reconsidered our temperature-dependent diffraction data by fitting the positions of the satellite peaks: we find that the in-plane periodicity of the polar vortices gradually decreases from 6.5 nm at 300 K to 5.5 nm at 433 K during heating (Figure R3 below). We have added this observation in the manuscript.
Upon cooling back to room temperature, the vortex periodicity recovers its original value, indicating that the change in periodicity with temperature is fully reversible.

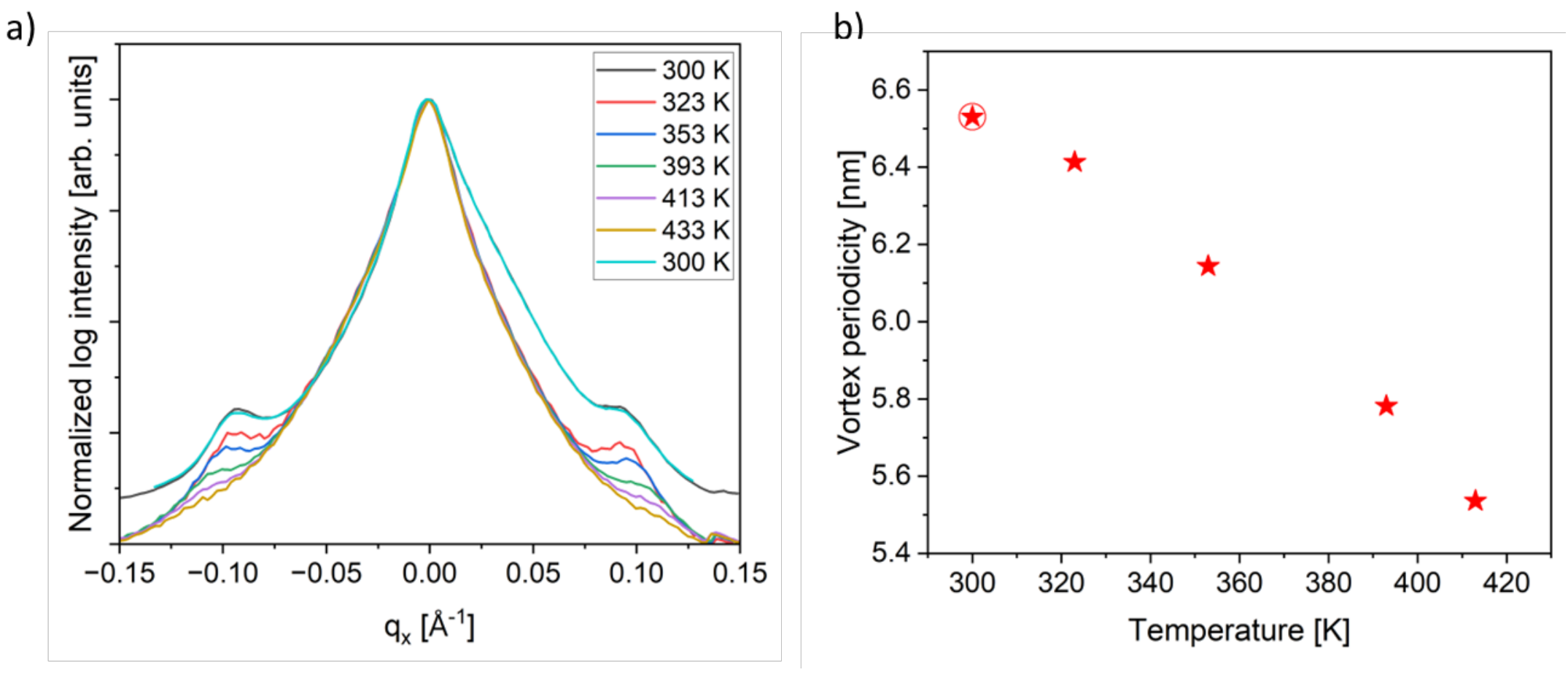

Figure R3: a) Line profiles extracted from the RSMs shown in Figure 5 of the manuscript, recorded at different temperatures. The black and turquoise curves correspond to RSMs at 300 K recorded before and after heating, respectively. The measurements done upon heating in the range 323-433 K were performed using a dome around the sample (which explains the lower scattering background compared to room temperature). b) Vortex periodicity as a function of temperature.

Modification to the manuscript:

- p.10, line 257: "The periodicity of the polar vortices gradually decreases upon heating, from 6.5 nm at 300 K down to 5.5 nm at 433 K (Figure S7 of the Supporting Information). Upon cooling the superlattice back to room temperature, the satellite peaks reappear at their original

positions and intensity. Hence, the change in periodicity with temperature is reversible and non-hysteretic."

- p.18, line 450: "The temperature-dependent RSM measurements show that the vortex periodicity decreases from 6.5 to 5.5 nm upon heating and disappears at the Curie temperature T_C of ~ 440 K. The same periodicity is recovered when cooling back to room temperature. High temperature STEM studies would be of interest to explore whether there are any microscopic changes during the heating or cooling processes."

Modification to Supporting Information file:

- p.13: We added Figure S10.

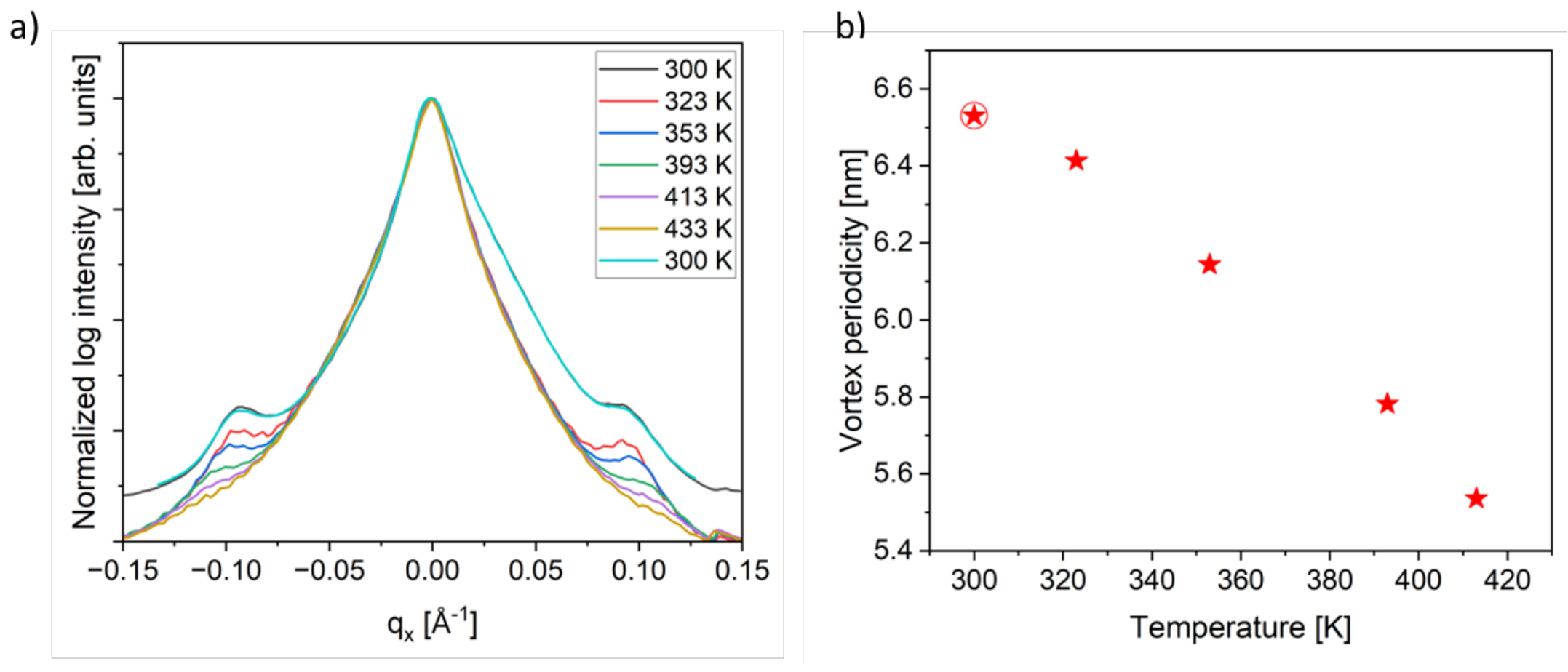

Figure S10: Temperature-dependent periodicity of the polar vortices in a (12 u.c. $BaTiO_3$/ 5 u.c. $SrTiO_3$)$_{10}$ superlattice on silicon Si (001). a) Line profiles extracted from the RSMs recorded at different temperatures, shown in Figure 5 of the manuscript. The black and turquoise curves correspond to RSMs at 300 K recorded before and after heating, respectively. The measurements done upon heating in the range 323-433 K were performed using a dome around the sample (which explains the lower scattering background compared to room temperature) b) Vortex periodicity as a function of temperature.

Comment 5: If it is possible, could the authors further discuss whether the vortices/polar waves are switchable under external stimuli (e.g., electric fields)? Given the distinct polarization states of individual BTO layers, it would also be interesting to clarify if such switching is controllable and reversible.

Response: We have attempted to probe the "switchability" of the polar vortices by macroscopic polarization vs. electric field (P-E) measurements (with patterned top Pt electrodes). However, these measurements are strongly affected by large leakage currents, much larger than the displacement current associated with ferroelectric switching. Therefore, the leakage current masks the ferroelectric response of the superlattices. In addition, the effective electric field is lower than the applied one due to the presence of a ~2.6 nm SiO_x interfacial layer between the Si p++ substrate and the superlattice. Since SiO_x has a much lower dielectric permittivity compared to $BaTiO_3$ and $SrTiO_3$, there is a strong voltage drop across SiO_x (approximately 40% of the applied voltage is dropped across the SiO_x layer), leaving only about 60% of the voltage dropped across the superlattice. Therefore, the applied electric field required to switch the polarization in $BaTiO_3$ needs to be considerably larger than the coercive field, which promotes very large leakage currents. The high leakage currents are most likely related to the presence of oxygen vacancies in the films.

The difficulty of achieving macroscopic P-E loops of MBE-grown $BaTiO_3$ films is also reflected in the scarcity of reports on this topic in the literature. To the best of our knowledge, there is only one report of macroscopic P-E loops in classical MBE-grown $BaTiO_3$ thin films on $Nb:SrTiO_3$ substrates (Yoneda *et al.*, *J. Appl. Phys.* **83**, 2458-2461 (1998)). Recently, using hybrid-MBE, macroscopic P-E loops were reported on $SrRuO_3/BaTiO_3/SrRuO_3$ heterostructures on $Nb:SrTiO_3$ substrates (Manjeshwar *et al.*, *Nano Lett.* **25**, 14246-14255 (2025)).

We are currently working on reducing leakage currents in our superlattices by doping the $BaTiO_3$ layers. The switching of polar vortices in superlattices will be the subject of a separate paper.

We have investigated the electrical switchability of our superlattices using PFM. Switching spectroscopy PFM shows a butterfly shape in the amplitude signal (Figure R4.a,b), and a hysteretic phase with a change of 180 degrees, which is the typical response of a ferroelectric material.
We were also able to pole stable domains of opposite polarization by scanning the sample surface with $\pm$ 6 V DC tip bias (the images of the domains are shown in Figure R4.c,d below). In the vertical phase signal, the brown spots in the yellow regions (poled down) are due to phase wrapping effects (as determined from the phase signals on these locations). We observe a significant electrostatic charging in the amplitude signal, which could be due to the presence of the top $SrTiO_3$ film.
These PFM experiments suggest that it is possible to unwind vortices under a large enough applied bias and that a remanent polarization can be stabilized.

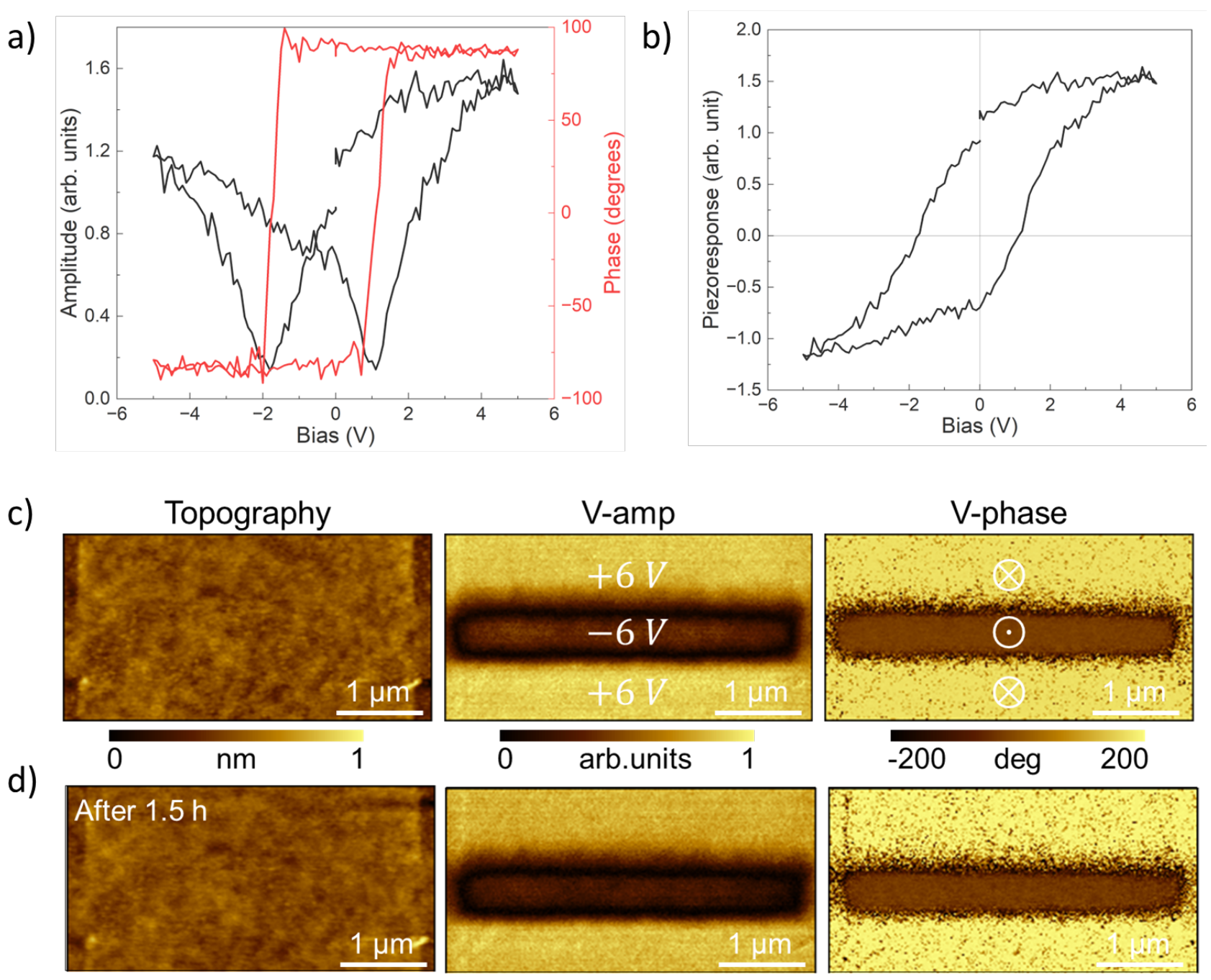

Figure R4: Piezoresponse force microscopy (PFM) on a (12 u.c. $BaTiO_3$/ 5 u.c. $SrTiO_3)_{10}$ superlattice on silicon Si(001). a) Switching spectroscopy PFM amplitude and phase signals, b) corresponding piezoloop c) Topography and PFM images (vertical amplitude and phase) of electrically poled regions (poling using ± 6 V DC). d) Same imaging after 1.5 hours. Imaging of the poled domains was done using an ac reading bias of 0.5 V. The black spots in the V-phase images observed in the bright regions (poled down) are due to phase wrapping (as determined from the phase signals on these locations).

Additionally, while phase field simulations have been used to illustrate the polarization distribution of the polar patterns, supplementing with nanoscale characterization of polar domains (e.g., piezoresponse force microscopy, PFM) would significantly strengthen the findings.

Response: We attempted to image the polar textures by PFM. However, the size of each individual vortex is of ~ 3.25 nm (the periodicity is of ~ 6.5 nm), much smaller than the resolution of standard PFM, which is limited by the size of the tip (~ 20 nm). Hence, we cannot resolve the polar vortices as their size is below the resolution limit.

Modification to the manuscript:

- p.10, line 245: "Note that we have attempted to image the polar vortices using piezo-response force microscopy (PFM) but could not resolve the polar pattern (the size of individual vortices is of ~ 3.5 nm) due to the tip-limited resolution (the diameter of the tip if of ~ 20 nm)"
- p. 19, line 464: "The electrical behavior of the superlattices could not be investigated by macroscopic measurements due to large leakage currents in $BaTiO_3$. Switching spectroscopy PFM shows a butterfly shape in the amplitude signal (Figure S13.a,b), and a hysteretic phase with a change of 180 degrees, which is the typical response of a ferroelectric material. We were also able to pole stable domains of opposite polarization by scanning the sample surface with ± 6 V DC tip bias (Figure S13.c,d). These experiments suggests that it is possible to unwind vortices under an applied bias and that a remanent polarization can be stabilized locally. Macroscopic measurements are needed to further explore the switchability of the vortices."
- p.19, line 493: "Ongoing and future work will focus on reducing the leakage currents in $BaTiO_3$, which is a critical step toward assessing their ferroelectric properties in micro- and nano-scale capacitors. The behavior and potential control of the vortices under electric field is of particular interest. There are several questions to be tackled: can the vortices be "switched" (from clockwise to anticlockwise and vice versa), can they be unwound and what happens when the electric field is switch off, can they be moved."

Modification to the Supporting Information file:

- p. 18: we added Figure S13.

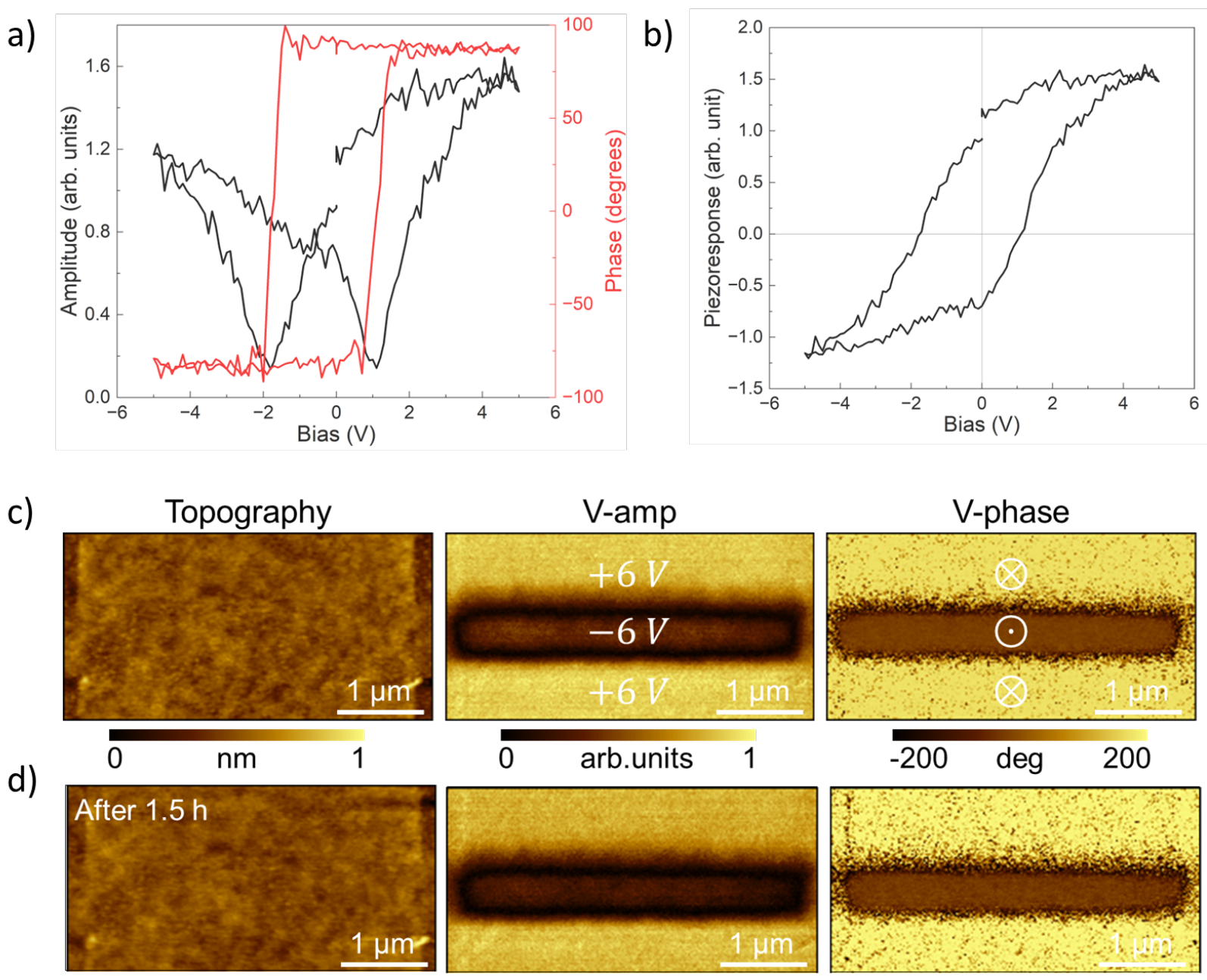

Figure S13: Piezoresponse force microscopy (PFM) on a (12 u.c. $BaTiO_3$/ 5 u.c. $SrTiO_3$)$_{10}$ superlattice on silicon Si (001). a) Switching spectroscopy PFM amplitude and phase signals, b) corresponding piezoloop c) Topography and PFM images (vertical amplitude and phase) of electrically poled regions (poling using ± 6 V DC). d) Same imaging after 1.5 hours. Imaging of the poled domains was done using an ac reading bias of 0.5 V. The black spots in the V-phase images observed in the bright regions (poled down) are due to phase wrapping (as determined from the phase signals on these locations).

We observe a significant electrostatic charging in the amplitude signal, which could be due to the presence of the top $SrTiO_3$ film.

Comment 6: Some minor issues should be improved. i.e., no Figure 2f in the manuscript (page 6, line 157); not all supplementary materials are explicitly referenced in the main text.

Response: Thank you for drawing our attention to these points. We have revised the references to figures throughout the manuscript. All supplementary materials are explicitly mentioned in the main text.

Modifications to the manuscript:

- p.7, line 173: "The displacement of the Ti ions relative to the barycenter of the Ba (Sr) atoms in each unit cell (Figure 2.a) was determined from…"
- p.10, line 238: "…ordered along the <110> directions of $BaTiO_3$ (Figures 4.a-c)."
- p.10, line 243: "…determined by Fast Fourier transform (Figure 4.b)…"

Reviewer #2

Remarks to the Author:

This work provided a comprehensive study of periodic polar vortices in BaTiO3/SrTiO3 superlattices epitaxially integrated onto silicon substrates, employing a synergistic combination of temperature-dependent XRD and atomic-resolution STEM to characterize the crystallographic symmetry and the long-range ordering of these topological textures. The experimental observations are further supported by phase-field simulations, which elucidate the critical role of substrate-induced tensile strain and interlayer coupling in stabilizing the vortex tubes. The manuscript is well-organized with high-quality data. However, there are a few issues need to be clarified before the manuscript can be considered for publication.

Comment 1: In Figure 1a, the author attributes the observed dislocations to thermal expansion mismatch. Since topological textures are extremely sensitive to local strain gradients, it is essential to discuss the spatial correlation between these dislocations and vortex. Do these dislocations act as pinning sites that stabilize the periodicity, or serve as sources of disorder that disrupt the long-range coherence of vortices?

Response: We thank the reviewer for raising this important question. This comment is very similar to Comment 1 from Reviewer 1 regarding the role of dislocations in the formation and periodicity of polar vortices.
The periodic vortex tubes are locally disrupted by dislocation defects. In the plan-view HAADF-STEM data (Figure R1 below which is a zoom on Figure 4.a in the manuscript), the dislocation defects appear as regions of dark contrast. The periodic stripe pattern terminates or locally loses

coherence in the regions where dislocations are present, indicating that these defects disturb the long-range lateral periodicity of the polar vortices.

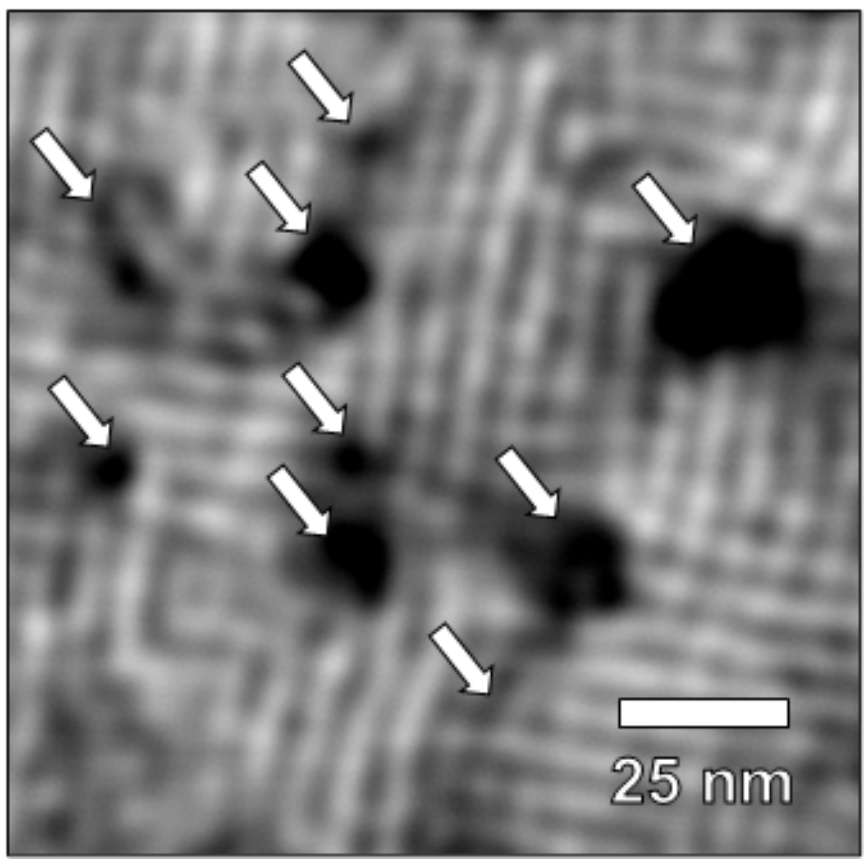

Figure R1: Plan-view STEM image of the $BaTiO_3$-$SrTiO_3$ superlattice containing dislocations. Regions of dark contrast marked by arrows are dislocations defects.

Strong and highly inhomogeneous strain fields around dislocations perturb the atomic columns, preventing an unambiguous determination of atomic positions and hindering the extraction of Ti-displacement vectors. Also, these dislocations alter the coupled electrostatic and elastic interactions that lead to the intrinsic polar ordering in the $BaTiO_3$/$SrTiO_3$ superlattices. As a result, the polar textures cannot be resolved in cross-sectional STEM near dislocations.

Modification to manuscript:

- p.7, line 179: Due to the strong and highly inhomogeneous strain fields around dislocations, the atomic positions and therefore the Ti-displacement vectors can not be precisely determined in their vicinity. Hence, we can not determine whether the dislocations have an impact on the periodicity of the polar patterns.
- p.10, line 239: The periodic vortex tubes are disrupted by dislocation defects which appear as regions of dark contrast in Figure 4.a. The periodic stripe pattern terminates or locally loses coherence in the regions where dislocations are present, indicating that these defects disturb the long-range lateral ordering of the polar vortices.

Comment 2: Noticeable discrepancy between the average periodicity measured by STEM (~7.5 nm) and XRD (~6.5 nm): while local structural variations often differ from macroscopic averages, a 15% difference is quite substantial for a long-range ordered system. The authors should confirm the periodicity by statistically analyzing larger areas of STEM images, or discuss whether this discrepancy indicates slight inhomogeneities in the superlattice growth.

Response: We thank the reviewer for this comment. To make the analysis more statistical, we have repeated the analysis of the periodicity on 4 different HAADF-STEM images that were taken from completely different locations on the sample. We made sure that the periodicity was measured over a lateral distance of at least 40 nm to capture several periods of the polar pattern.

On the different images, we measured periodicities of 6.6 nm, 7.5 nm, 6.4 nm and 7.0 nm. From these measurements, we obtain an average periodicity of 6.9 nm, which is lower than the value previously reported in the manuscript and closer to the periodicity determined from RSM (6.5 nm). These measurements also indicate that there are inhomogeneities in the superlattice.

Modification to manuscript:

- p.7, 175: "We determined an average vortex periodicity of ~ 6.9 nm from several STEM images recorded on different locations of the sample, which is close to the ~ 6.5 nm periodicity determined by RSM (there are slight inhomogeneities in the superlattice as indicated by the dispersion of the observed periodicity ranging from 6.4 nm to 7.5 nm depending on the location on the sample)."

- p.10, line 242: "The average periodicity along <110> is of ~7.0 nm as determined by Fast Fourier transform (Figure 4.b), quite close to the value determined from cross-view lamellae (~6.9 nm)."

Comment 3: The author demonstrates the stability of the vortex state up to 450 K. Considering the significant thermal expansion mismatch, does the vortex periodicity exhibit hysteretic behavior during heating-cooling cycles?

Response: We thank the reviewer for this comment. As described in our response to Comment 4 of Reviewer 1, we observe that the periodicity of the polar pattern decreases from 6.5 nm at 300 K to 5.5 nm at 433 K, disappears fully at 453 K, and recovers to 6.5 nm when cooled back to room temperature. Unfortunately, we do not have diffraction data acquired at the different temperatures when cooling from high temperature to room temperature. But the fact that the periodicity returns to its exact same value at room temperature indicates that there is no hysteretic effect.

In our $BaTiO_3/SrTiO_3$ superlattice, the ferroelectric-paraelectric phase transition is not abrupt as can be seen from temperature-dependent Raman data presented in Figure 5 (consistent with other reports on $BaTiO_3$ thin films, e.g. El Marssi *et al.*, *J. Appl. Phys.* **94**, 3307-3312 (2003)).

In response to Comment 7 by Reviewer 3, we have investigated the temperature-dependent evolution of the out-of-plane and in-plane lattice parameters by temperature-dependent XRD and these measurements bring also a partial response to the present comment. We do not observe hysteresis of the lattice parameters between heating and cooling (Figure S12 shown below). This result is in good agreement with the fact that the vortex periodicity is not hysteretic at room temperature. From the absence of hysteresis for the lattice parameters, we do not expect hysteretic behavior of the vortex periodicity upon heating/cooling phase. However, small path-dependent effects cannot be entirely excluded due to non-linear strain-polarization coupling, or ferroelastic domain rearrangements in relation to defects. It is also possible that the rate of cooling (and how much higher than the Tc the sample was heated – in the present case Tc was not much exceeded) may have an impact on the formation of the vortices during cooling. This would need to be investigated with a dedicated beamtime. In our experimental conditions, we can conclude that no hysteresis was found.

Modification to manuscript:

- p.10, line 257: "The periodicity of the polar vortices gradually decreases upon heating, from 6.5 nm at 300 K down to 5.5 nm at 433 K (Figure S7 of the Supporting Information). Upon cooling the superlattice back to room temperature, the satellite peaks reappear at their original positions and intensity. Hence, the change in periodicity with temperature is reversible and non-hysteretic."

- p.11, line 269: "In XRD, a change in slope for the *c*-axis lattice parameter as a function of temperature (Figure S9) appears at 449 K (176 °C), in very good agreement with the Raman spectroscopy results, considering the different set-up/temperature measurements. The T_c of the

superlattice is therefore in the range 440 – 450 K. Note that there is no hysteretic behavior of the lattice parameters upon heating/cooling."

- p.18, line 450: "The temperature-dependent RSM measurements show that the vortex periodicity decreases from 6.5 to 5.5 nm upon heating and disappears at the Curie temperature T_c of ~ 440 K. The same periodicity is recovered when cooling back to room temperature. "

Modification to Supporting Information file:

- p.13: We added Figure S10.

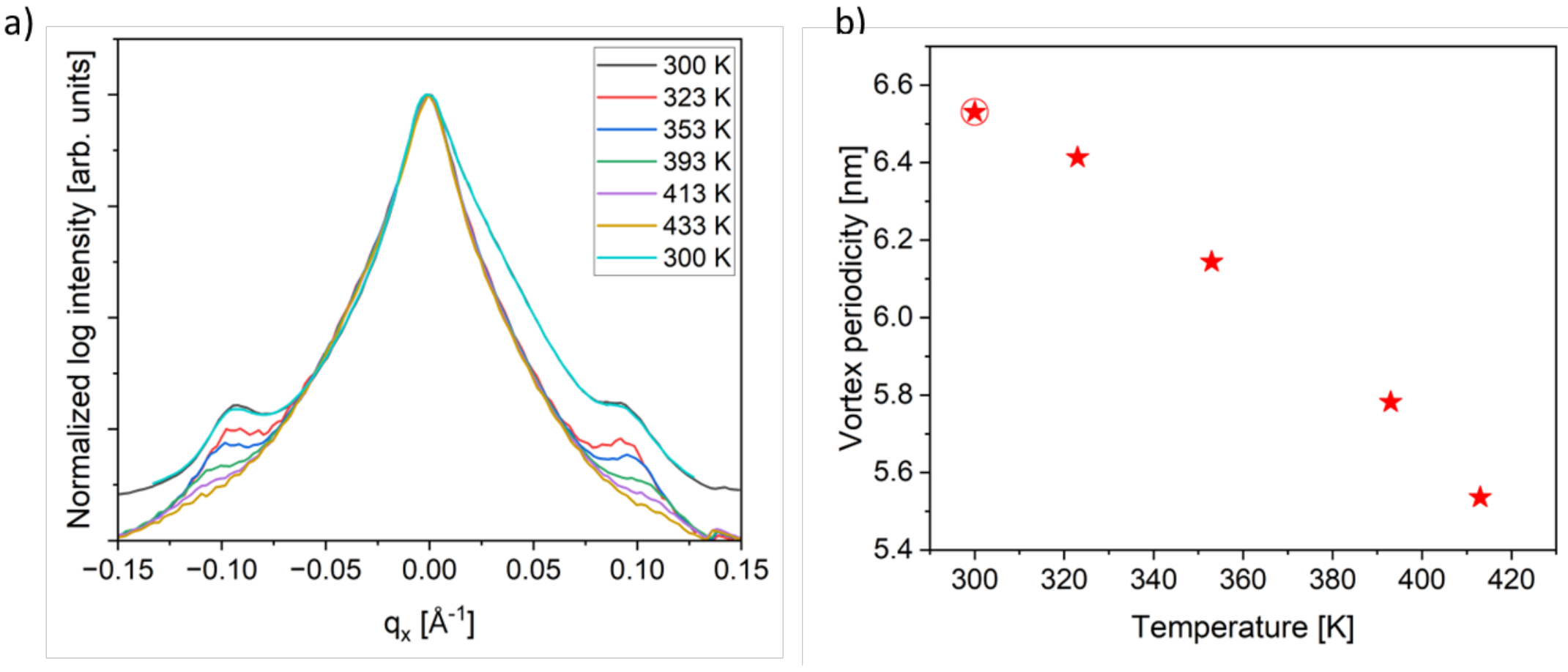

Figure S10: Temperature-dependent periodicity of the polar vortices in a (12 u.c. $BaTiO_3$/ 5 u.c. $SrTiO_3)_{10}$ superlattice on silicon Si (001). a) Line profiles extracted from the RSMs recorded at different temperatures, shown in Figure 5 of the manuscript. The black and turquoise curves correspond to RSMs at 300 K recorded before and after heating, respectively. The measurements done upon heating in the range 323-433 K were performed using a dome around the sample (which explains the lower scattering background compared to room temperature) b) Vortex periodicity as a function of temperature.

- p. 14: We added Figure S11.

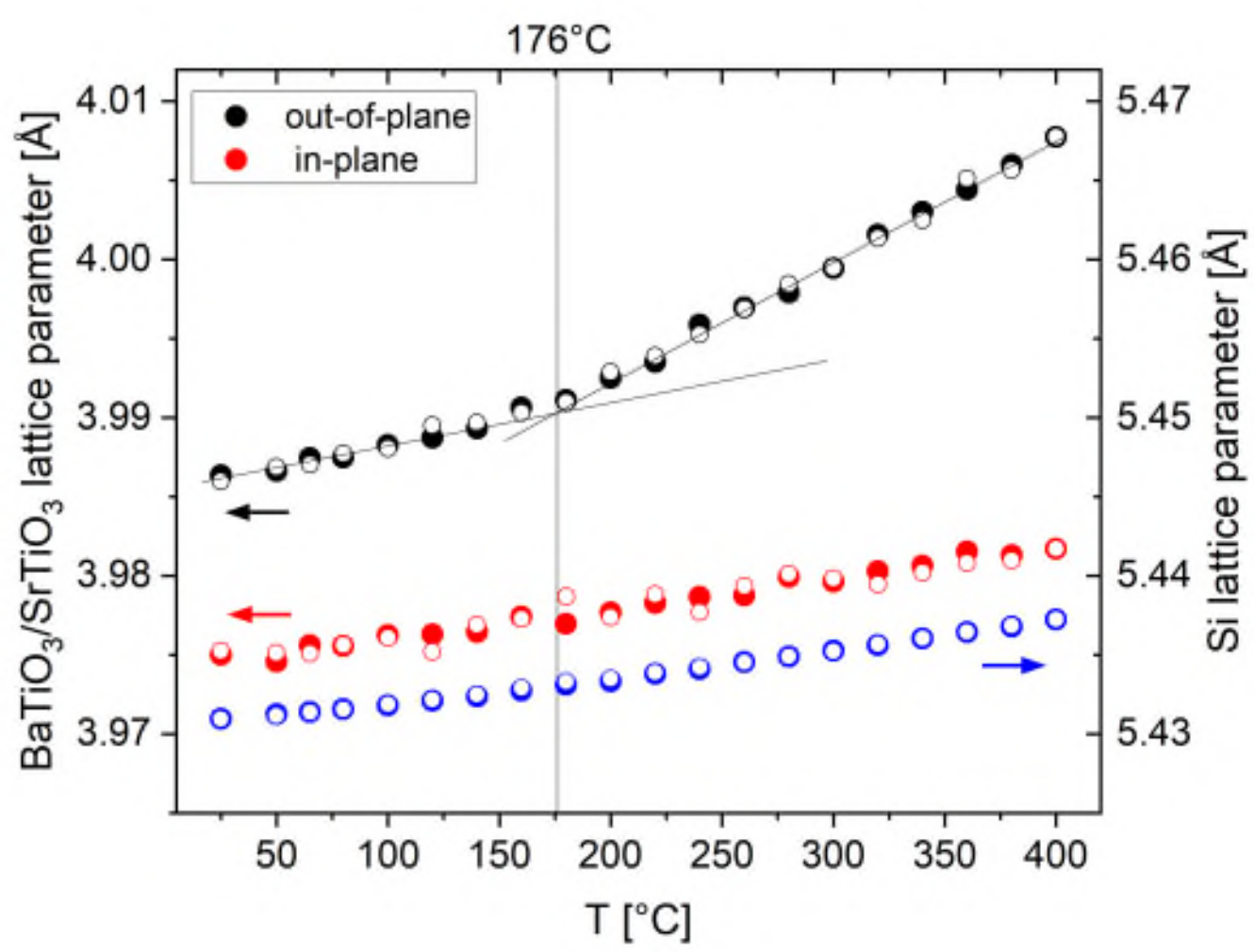

Figure S11: **Temperature-dependent lattice parameters of the (12 u.c. $BaTiO_3$/ 5 u.c. $SrTiO_3)_{10}$ superlattice on Si (001) and of the silicon substrate.** Full and open circles correspond to lattice parameters extracted during sample heating and cooling, respectively.

Note: the larger thermal expansion rate above the phase transition (here at ~ 176 °C) is consistent with reports for $BaTiO_3$ ceramics (Y. He, *Thermochim. Acta* **419**, 135 (2004)).

Comment 4: On Page 9, the author mentioned that the TC (~440 K) of superlattices is higher than that of bulk materials (~393 K). For thin layers (< 5 nm), the strong depolarization field typically leads to a sharp reduction in TC due to the finite-size effect. While the authors emphasize the stability of the residual tensile strain from the silicon substrate, it is necessary to clarify how the strain-induced enhancement compensates for and eventually exceeds the reduction in TC?

Response: We thank the reviewer for the question. We should indeed add a short discussion in the manuscript.
The T_c of ferroelectric thin films is indeed reduced when the thickness is decreased due to the depolarization field. However, the strain state has also a determining role in T_c. Ultrathin (below 5 nm) epitaxial $BaTiO_3$ films have been shown to have T_c exceeding the bulk's T_c (120 – 130°C, 393 – 403 K). For a 4.0 nm $BaTiO_3$ film fully strained to $SrTiO_3$, Tenne *et al.* reported a T_c (experimental) of ~525 K if the film was uncapped and of ~560 K if the film was capped with a 10 nm $SrTiO_3$ film (Tenne *et al.*, *Phys. Rev. Lett.* **103**, 177601 (2009)). If we extrapolate their results, the T_c for **a 4.8 nm $BaTiO_3$ film** fully strained to $SrTiO_3$ is of ~ **740 K**. We have shown in Olaniyan *et al.* (Advanced Functional Materials 35, e07905 (2025)), that $BaTiO_3$ is not fully strained on $SrTiO_3$-templated-Si but partially relaxed, which lead to a decrease of T_c compared to the fully strained-to-$SrTiO_3$ case.
If we consider the case of $BaTiO_3/SrTiO_3$ superlattices, there are also measurements available in literature for superlattices grown on $SrTiO_3$ (Tenne *et al.*, *Science* **313**, 1614 (2006)). For **($BaTiO_3$ 8 u.c./$SrTiO_3$ 4 u.c.)$_{10}$**, they report a T_c value of ~ **640 K** (note that this is the same number of bilayers as in our study), demonstrating that the strain imposed by the substrate can outweigh the reduction in T_c caused by the finite-size effect. The extrapolation of their curve representing T_c as a function of n in ($BaTiO_3$ n u.c./$SrTiO_3$ 4 u.c.)$_{10}$ for n = 12 u.c. (Fig. 4 of their paper) would give a T_c much larger than 700 K. Increasing the $SrTiO_3$ thickness to 5 u.c. (like in our case) will not impact significantly T_c as can be seen with their study for 13 u.c. $SrTiO_3$. Hence, as seen in literature, despite the depolarization field, the Curie temperature in ultrathin $BaTiO_3$ films (< 4.8 nm = 12 u.c.) in superlattices is significantly larger than that in bulk $BaTiO_3$ due to strain. In our case, since $BaTiO_3$ is not fully strained to $SrTiO_3$, T_c is smaller but still significantly larger than the bulk value.
Note also that the presence of rotational polarization in $BaTiO_3$ layers in our superlattice and the finite polarization in the $SrTiO_3$ layers create a complex electrostatic environment in which the depolarization field is partially screened and redistributed, instead of suppressing ferroelectricity.

Modification to manuscript:

- p.11, line 276: "It is well known that The T_c of ferroelectric thin films is reduced when the thickness is decreased due to the depolarization field. However, the strain state has also a determining role in T_c. Ultrathin (below 5 nm) epitaxial $BaTiO_3$ films have been shown to have T_c exceeding the bulk's T_c. For a 4.0 nm $BaTiO_3$ film fully strained to $SrTiO_3$, Tenne *et al.* reported a T_c (experimental) of ~525 K if the film was uncapped and of ~560 K if the film was capped with a 10 nm $SrTiO_3$ film (Tenne *et al.*, *Phys. Rev. Lett.* **103**, 177601 (2009)). If we extrapolate their results, the T_c for a 4.8 nm $BaTiO_3$ film fully strained to $SrTiO_3$ is of ~ 740 K. For superlattices like ($BaTiO_3$ 8 u.c./$SrTiO_3$ 4 u.c.)$_{10}$ grown on $SrTiO_3$, a T_c value of ~ 640 K was reported and the extrapolation to ($BaTiO_3$ 12 u.c./$SrTiO_3$ 4 u.c.)$_{10}$ give a T_c much larger than 700 K [Tenne *et al.*, *Science* **313**, 1614 (2006)]. Hence, despite the depolarization field, the Curie temperature in ultrathin $BaTiO_3$ films (< 5 nm) fully strained to $SrTiO_3$ in superlattices is significantly larger than that in bulk $BaTiO_3$. In our case, since $BaTiO_3$ is not fully strained to $SrTiO_3$ but partially relaxed (Olaniyan *et al.*, Adv. Funct. Mater. 35, e07905 (2025)), T_c is smaller but still significantly larger than the bulk value."

Comment 5: As the authors stated, topological polar textures have been reported frequently. These patterns are scientifically interesting since they differ from typical ferroelectric domain patterns. However, for people new to this area, it is not clearly introduced how these exotic polar patterns can be used in potential applications. I believe some introduction to the potential use will appeal to a broader readership of this manuscript.

Response: Thank you for this suggestion. We have added a paragraph which introduces the potential applications of polar textures in electronic devices (topotronics) to the Introduction of the manuscript.

Modifications to manuscript:

p.3, line 55: "Hence, polar topologies offer opportunities for ultra high-density memory devices and for novel functionalities in low-power electronics, or photonics, broadly captured under the term topotronics[32]. Well-known examples in this context are magnetic racetrack memories based on magnetic skyrmions[33,34]. Analogue to this, recent theoretical predictions of high electric-field-driven mobilities (25 m/s) of polar skyrmion bubbles[35] suggest the possibility of realizing electric racetrack memory devices. Electrically driven architectures could offer improved energy efficiency compared to their magnetic counterparts. In addition, the highly tunable dielectric response and negative capacitance behavior of polar textures under applied electric fields could be exploited in low-power and reconfigurable electronic components, such as adaptive capacitors or steep-slope transistors[29,36]. Recent progress in the manipulation of polar textures using external stimuli, such as electric fields and light, reveals exciting opportunities for creating novel device functionalities. For instance, the erasure of the toroidal order of polar vortices[21,37] and the switching of vortex tube chirality[31] demonstrate the possible manipulation of these polar structures. Furthermore, the recent discovery of ultra-fast electric field responses from polar vortices in the sub-THz regime highlights their potential for high-speed data processing and storage applications[38]. Also, the interconversion of different polar topologies through light and temperature show exciting pathways to engineering polar architectures[17,24,39–42]".

Reviewer #3

We appreciate the thorough review and questions raised. However, we would like to point out that the goal of the review is not to increase the scope of the study with some requests of several additional aspects to address (potential effects of vortices on piezoelectricity and thermoelectricity effects, the dynamic behavior of the vortices) and several discussions to add about fields that are out of our topic (electrochemical stability, solid-state batteries, solid-state battery interface layers, electrocatalysis, ion migration…).

We have addressed most of the comments and performed new experiments (EELS, EDX, PFM, temperature-dependent XRD). Five new figures have been added to the Supporting Information file.

Remarks to the Author: The work being carried out by the author is meaningful and it is hoped that the following issues can be explained.

Comment 1: The conclusion part of the paper emphasizes the significance of realizing topological structures on silicon for "topotronic devices", but it is too broad. It is suggested to add a dedicated paragraph that combines the negative capacitance effect of ferroelectric materials, controllable ionic transport, and interface charge modulation, and specifically discusses its potential applications in new resistive memories, solid-state battery interface layers, and electrocatalytic

carriers in energy information devices. This will greatly enhance the interdisciplinary influence of the paper.

Response: Thank you for your suggestion. We have added a paragraph introducing topotronics and highlighting the potential applications of polar textures in electronic devices.

We respectfully disagree that we should discuss, in addition, controllable ionic transport, solid-state battery interfaces, or electrocatalytic carriers in energy information devices. The literature on polar textures do not address such topics. We do not think that it will enhance the interdisciplinary influence of the paper but that **it would, on the contrary, oversell the topic of polar textures.**

Modifications to manuscript:

- p.3, line 55: "Hence, polar topologies offer opportunities for ultra high-density memory devices and for novel functionalities in low-power electronics, or photonics, broadly captured under the term topotronics[32]. Well-known examples in this context are magnetic racetrack memories based on magnetic skyrmions[33,34]. Analogue to this, recent theoretical predictions of high electric-field-driven mobilities (25 m/s) of polar skyrmion bubbles[35] suggest the possibility of realizing electric racetrack memory devices. Electrically driven architectures could offer improved energy efficiency compared to their magnetic counterparts. In addition, the highly tunable dielectric response and negative capacitance behavior of polar textures under applied electric fields could be exploited in low-power and reconfigurable electronic components, such as adaptive capacitors or steep-slope transistors[29,36]. Recent progress in the manipulation of polar textures using external stimuli, such as electric fields and light, reveals exciting opportunities for creating novel device functionalities. For instance, the erasure of the toroidal order of polar vortices[21,37] and the switching of vortex tube chirality[31] demonstrate the possible manipulation of these polar structures. Furthermore, the recent discovery of ultra-fast electric field responses from polar vortices in the sub-THz regime highlights their potential for high-speed data processing and storage applications[38]. Also, the interconversion of different polar topologies through light and temperature show exciting pathways to engineering polar architectures[17,24,39–42]".

Comment 2: As the active layer that may be used in the device, the $BaTiO_3/SrTiO_3$ superlattice's stability in an electrochemical environment is the key to its practical application. The author has overlooked the preliminary assessment of electrochemical stability. It is suggested to mention this issue in the discussion section, or to indicate that this is a direction that must be studied before integrating it into energy devices such as solid-state batteries.

Response: Polar topological textures are generally not discussed in the context of energy storage applications such as solid-state batteries or electrochemical stability. Here, we demonstrate the realization of polar topological textures monolithically integrated on silicon in $BaTiO_3/SrTiO_3$ superlattices, a system that does not contain lead, with relevance to fundamental physics of ferroelectrics and CMOS-compatibility for nanoelectronic devices. As such, the materials are not operated in an electrochemical environment, and an assessment or discussion of electrochemical stability in view of their integration in solid state batteries is outside the scope of the present work.

Comment 3: The paper repeatedly mentions CMOS compatibility, but does not deeply discuss the challenges that this topological structure may face when integrated with existing silicon-based chip processes (such as thermal budget, etching, electrode materials). It is suggested to add relevant discussions.

Response: We agree that several challenges remain before such topological ferroelectric structures could be integrated into practical silicon-based devices. We have not discussed deeply the

challenges for integration of topological textures into CMOS because it is not the topic of this paper, and the introduction is already detailed enough on several aspects. We would have done it in the context of a perspective paper or review paper.

The epitaxial growth of $BaTiO_3/SrTiO_3$ superlattices requires a temperature (650 °C in this study) that is totally fine for FEOL processes (dopant activations are done at a temperature exceeding 1000°C) but exceeds the thermal limit of back-end-of-line processing (400-450°C). However, solutions exists to integrate perovskite oxides in the BEOL of CMOS chips, such as wafer bonding, which has been shown effectively by IBM Zürich (J. Fompeyrine, S. Abel – now at LUMIPHASE) for $BaTiO_3$ for photonic applications (we have added a reference to the manuscript).

Etching itself is not an issue for CMOS compatibility but requires optimization. $BaTiO_3$ can be etched by either Ar milling (used for PCRAM) or reactive ion etching. When $SrTiO_3$ was considered as a potential high-K dielectric to replace SiO_2 in MOSFETs (early 2000's) by companies like Motorola, etching was not the issue for integrating a perovskite on Si. The last author of this paper (Prof, C. Dubourdieu) has fabricated transistors at IBM in Yorktown Heights, using the CMOS clean room and adapting all processes to $BaTiO_3$ (results not published).

Modifications to manuscript:

We have added a short discussion in the conclusion.

- p.19, line 488: "This work marks a first step for bringing functionality of topological polar textures into the world of nanoelectronics, but several challenges remain before such materials could be integrated into practical CMOS nanoscale devices. Further studies are required to evaluate the impact of patterning and etching processes on the integrity of the topological textures. If thermal budget constraints restrict high-temperature oxide growth to front-end-of-line architectures, approaches such as wafer bonding can be used for back-end-of-line integration[86]. Ongoing and future work will focus on reducing the leakage currents in $BaTiO_3$, which is a critical step toward assessing their ferroelectric properties in micro- and nano-scale capacitors"

Comment 4: The periodic vortex array and strain coupling observed in the paper may generate enhanced or novel piezoelectric/thermoelectric responses? The piezoelectric/thermoelectric effect of ferroelectric materials is the key to energy harvesting. The author is requested to explain this.

Response: Thank you for raising this interesting point. However, we would like to emphasize that these aspects are also beyond the scope of our paper. We can not raise all the topics related to ferroelectric materials in this research paper (which is not a review paper).

Concerning the piezoelectric response of our $BaTiO_3/SrTiO_3$, we have performed PFM measurements on our sample and observe a conventional ferroelectric response, as discussed in our response to Comment 5 of Reviewer 2.

Enhanced piezoelectric behavior has been reported in vortex-containing ferroelectric superlattices composed of two switchable ferroelectric layers, such as $PbTiO_3/(Pb_xSr_{1-x})TiO_3$ grown on $DyScO_3$ with $SrRuO_3$ electrodes (Kavle *et al.*, *Adv. Mater.* **26**, 2410146 (2024)). In that case, the enhancement was attributed to the coupled switching of both ferroelectric layers. A quantitative assessment of a potential electromechanical enhancement in our ($BaTiO_3/SrTiO_3$) would require direct measurements of piezoelectric coefficients (e.g., d_{33}) using interferometric techniques, which are beyond the scope of the present work.
Similarly, while the interplay between strain and polarization could in principle influence thermoelectric responses, a meaningful evaluation would require dedicated electro-caloric measurements. This is not the scope of our study.

Comment 5: In electrochemical devices, ion migration will greatly affect performance. For this topological structure with a strong polarization gradient and complex built-in electric field, will it affect or regulate the distribution and migration of oxygen vacancies and other defects?

Response: While ion migration is indeed a critical issue in electrochemical devices, the polar topological textures investigated here are not intended for electrochemical or ionic-conduction applications.

The presence of oxygen vacancies induce large leakage currents that prevent us from doing macroscopic P-E loop measurements on capacitors, as we explain in our response to Comment 6 below.

It has been reported that in $PbTiO_3/SrTiO_3$ superlattices grown on $DyScO_3$ substrates with $SrRuO_3$ bottom electrodes, oxygen vacancies can be stabilized in the cores of polar vortices due to the local electrostatic potential landscape (Du *et al.*, *Nat. Commun.* **10**, 4864 (2019)).

In our systems, in case of an inhomogeneous distribution of oxygen vacancies in the $BaTiO_3$ layers, one signature would be a change in the Ti L response in EELS. We have performed EELS measurements on the Ti L edges at different lateral locations of a $BaTiO_3$ layer (to probe both edges and cores of vortices). The results are shown in Figure R5 below. We do not see any significant changes in the Ti signature. Hence, our experimental data do not indicate vacancy accumulation – at least in a detectable amount – in some locations of the polar textures.

We do not include these results in the manuscript or Supporting Information file as we think that additional measurements would be necessary to conclude (improve energy resolution, compare scans performed horizontally and vertically).

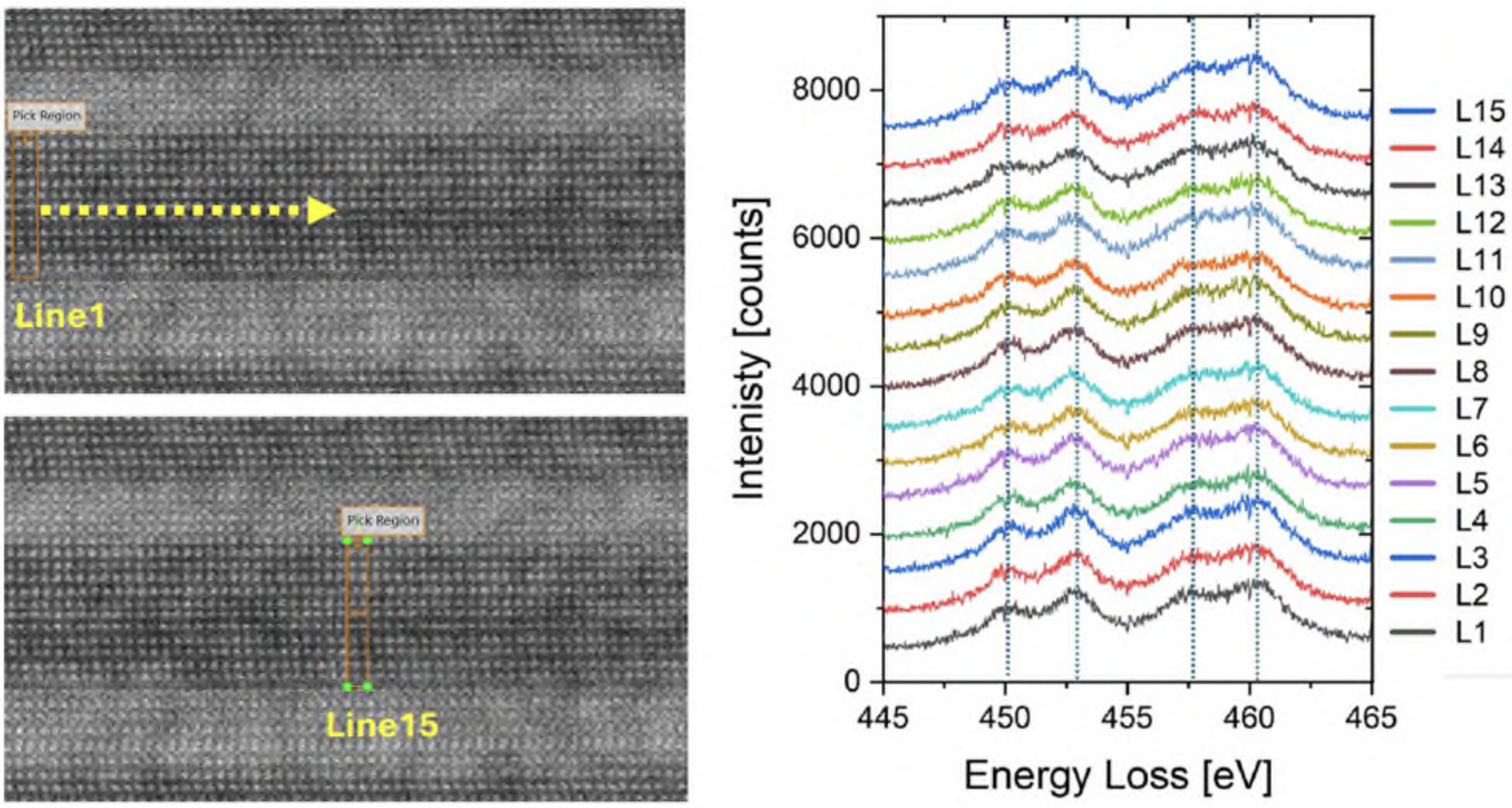

Figure R5: STEM-EELS analysis of Ti $L_{2,3}$ core-loss edges across the BTO layer. The Ti EELS spectra were extracted by integrating the signal within vertical rectangular regions spanning approximately 10 unit cells, as illustrated on the left. The integration boxes were sequentially shifted from left to right to obtain spectra from 15 distinct positions (L1–L15). The corresponding stacked spectra are shown on the right, with dashed lines indicating the characteristic Ti L_3 and L_2 peak positions.

Comment 6: The author indirectly proved the polarization vortex through displacement mapping, but lacks measurement of the overall macroscopic ferroelectric properties of the superlattice (such as the P-E hysteresis loop, leakage current characteristics). This is not conducive to evaluating its quality as a ferroelectric functional layer, and lacks direct measurement of key ferroelectric properties, so the author is requested to make a supplement.

Response: Thank you for this comment. We have attempted to perform (P-E) measurements on capacitors with Si p++ as the bottom electrode and patterned W top electrodes.

However, these measurements are strongly affected by large leakage currents, much larger than the displacement current associated with ferroelectric switching. Therefore, the leakage current masks the ferroelectric response of the superlattices.
In addition, the effective electric field is lower than the applied one due to the presence of a ~2.6 nm SiO_x interfacial layer between the Si p++ substrate and the superlattice. Since SiO_x has a much lower dielectric permittivity compared to $BaTiO_3$ and $SrTiO_3$, there is a strong voltage drop across SiO_x (approximately 40% of the applied voltage is dropped across the SiO_x layer), leaving only about 60% of the voltage dropped across the superlattice. Therefore, the applied electric field required to switch the polarization in $BaTiO_3$ needs to be considerably larger than the coercive field, which promotes very large leakage currents. The high leakage currents are most likely related to the presence of oxygen vacancies in the films.

The difficulty of achieving macroscopic P-E loops of MBE-grown $BaTiO_3$ films is also reflected in the scarcity of reports on this topic in the literature. To the best of our knowledge, there is only one report of macroscopic P-E loops in classical MBE-grown $BaTiO_3$ thin films on Nb:$SrTiO_3$ substrates (Yoneda *et al.*, *J. Appl. Phys.* **83**, 2458-2461 (1998)). Recently, using hybrid-MBE, macroscopic P-E loops were reported on $SrRuO_3$/$BaTiO_3$/$SrRuO_3$ heterostructures on Nb:$SrTiO_3$ substrates (Manjeshwar *et al.*, *Nano Lett.* **25**, 14246-14255 (2025)).

We are currently working on reducing leakage currents in our superlattices by doping the $BaTiO_3$ layers. The switching of polar vortices in superlattices will be the subject of a separate paper.

We have investigated the ferroelectricity of our superlattices using piezoresponse force microscopy (PFM). Switching spectroscopy PFM shows a butterfly shape in the amplitude signal (Figure R4.a,b), and a hysteretic phase with a change of 180 degrees, which is the typical response of a ferroelectric material.
We were also able to pole stable domains of opposite polarization by scanning the sample surface with $\pm$ 6 V DC tip bias (the images of the domains are shown in Figure R4.c,d below). In the vertical phase signal, the brown spots in the yellow regions (poled down) are due to phase wrapping effects (as determined from the phase signals on these locations). We observe a significant electrostatic charging in the amplitude signal, which could be due to the presence of the top $SrTiO_3$ film.
These PFM experiments suggest that it is possible to unwind vortices under a large enough applied bias and that a remanent polarization can be stabilized.

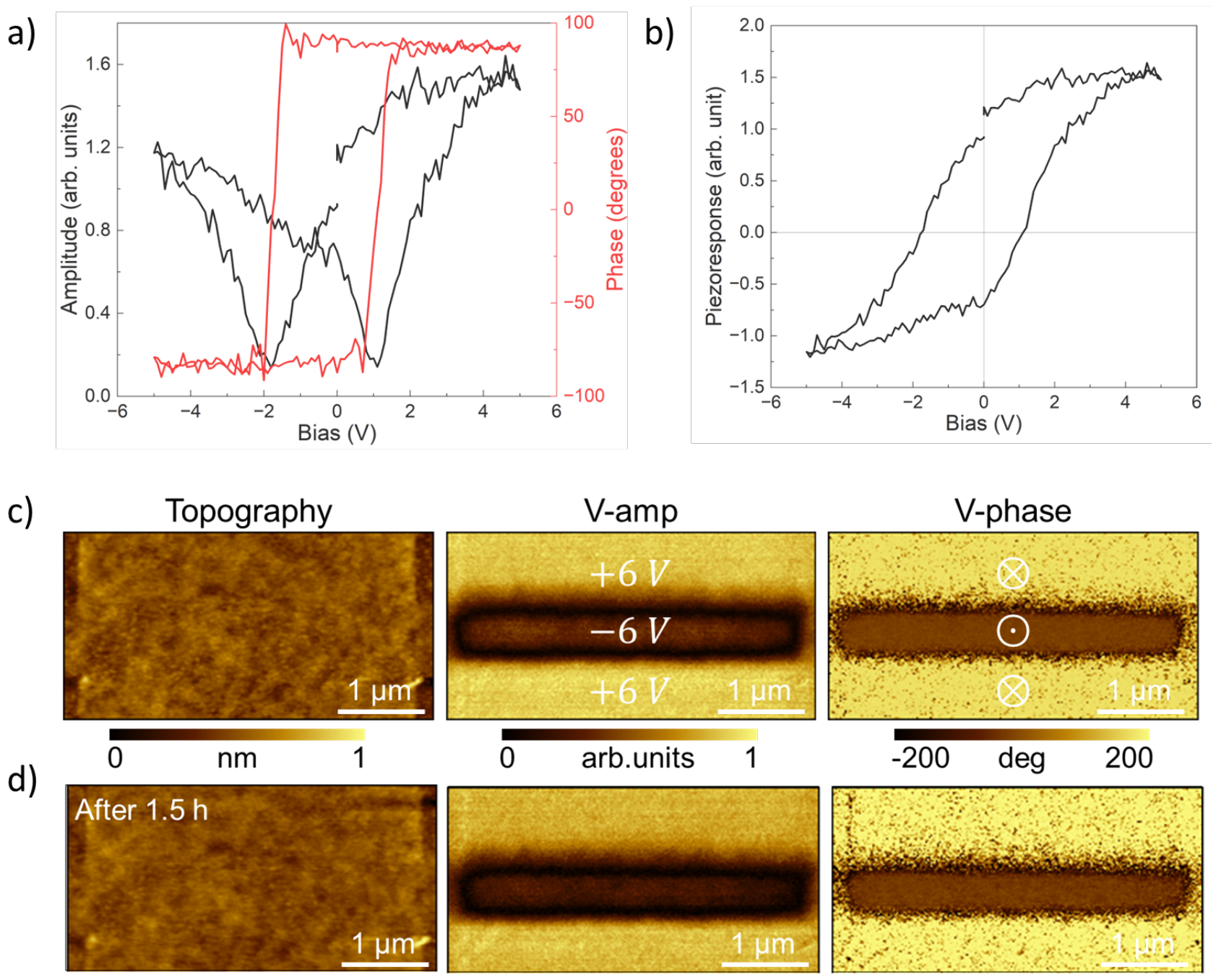

Figure R4: Piezoresponse force microscopy (PFM) on a (12 u.c. $BaTiO_3$/ 5 u.c. $SrTiO_3$)$_{10}$ superlattice on silicon Si (001). a) Switching spectroscopy PFM amplitude and phase signals, b) corresponding piezoloop c) Topography and PFM images (vertical amplitude and phase) of electrically poled regions (poling using ± 6 V DC). d) Same imaging after 1.5 hours. Imaging of the poled domains was done using an ac reading bias of 0.5 V. The black spots in the V-phase images observed in the bright regions (poled down) are due to phase wrapping (as determined from the phase signals on these locations).

Modification to the manuscript:

- p. 19, line 464: "The electrical behavior of the superlattices could not be investigated by macroscopic measurements due to large leakage currents in $BaTiO_3$. Switching spectroscopy PFM shows a butterfly shape in the amplitude signal (Figure S13.a,b), and a hysteretic phase with a change of 180 degrees, which is the typical response of a ferroelectric material. We were also able to pole stable domains of opposite polarization by scanning the sample surface with ± 6 V DC tip bias (Figure S13.c,d). These experiments suggest that it is possible to unwind vortices under an applied bias and that a remanent polarization can be stabilized locally. Macroscopic measurements are needed to further explore the switchability of the vortices."

- p.19, line 493: "Ongoing and future work will focus on reducing the leakage currents in $BaTiO_3$, which is a critical step toward assessing their ferroelectric properties in micro- and nano-scale capacitors. The behavior and potential control of the vortices under electric field is of particular interest. There are several questions to be tackled: can the vortices be "switched" (from clockwise to anticlockwise and vice versa), can they be unwound and what happens when the electric field is switch off, can they be moved."

Modification to the Supporting Information file:

- p. 18: we added Figure S13.

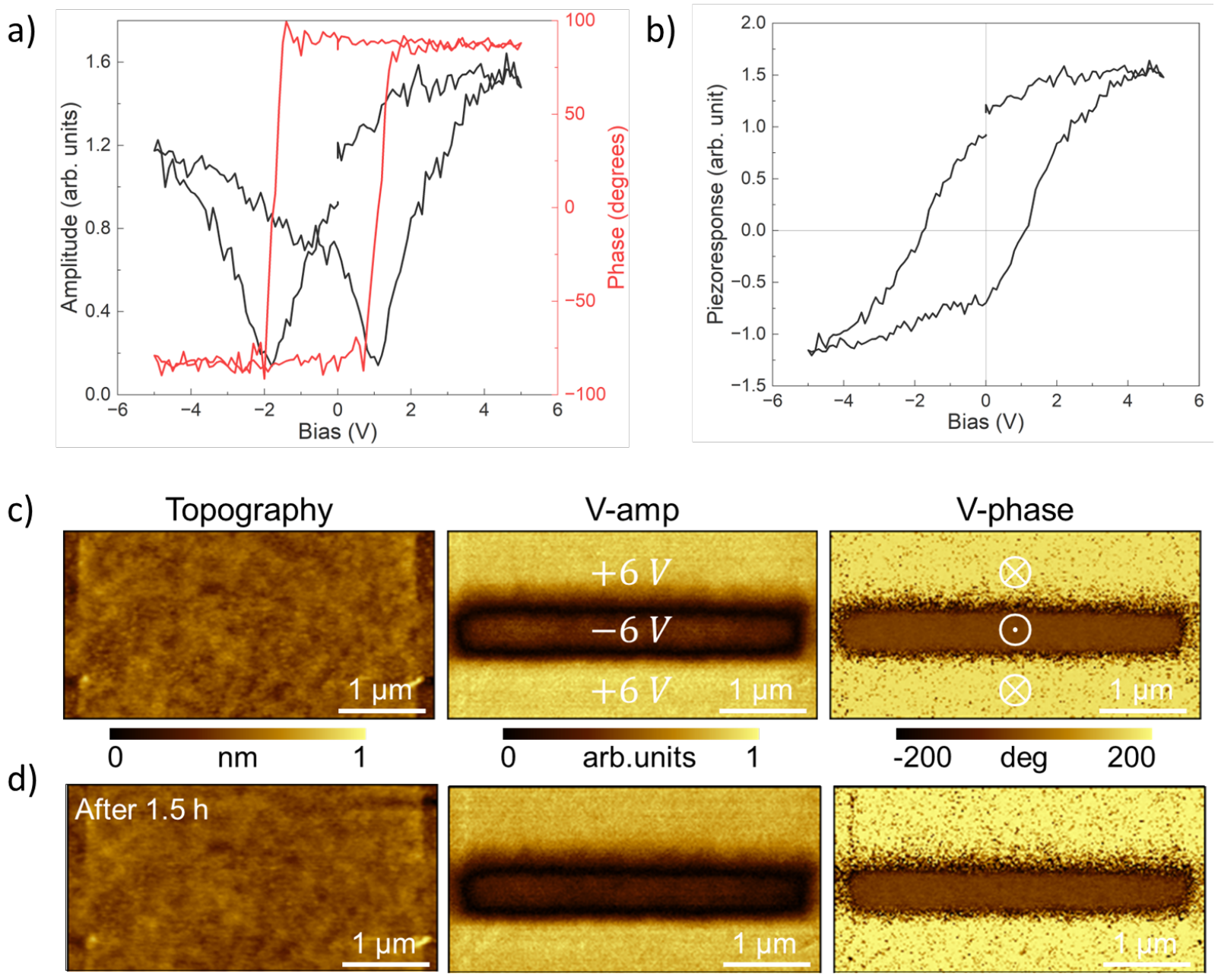

Figure S13: Piezoresponse force microscopy (PFM) on a (12 u.c. $BaTiO_3$/ 5 u.c. $SrTiO_3$)$_{10}$ superlattice on silicon Si (001). a) Switching spectroscopy PFM amplitude and phase signals, b) corresponding piezoloop, c) Topography and PFM images (vertical amplitude and phase) of electrically poled regions (poling using ± 6 V DC). d) Same imaging after 1.5 hours. Imaging of the poled domains was done using an ac reading bias of 0.5 V. The black spots in the V-phase images observed in the bright regions (poled down) are due to phase wrapping (as determined from the phase signals on these locations).

We observe a significant electrostatic charging in the amplitude signal, which could be due to the presence of the top $SrTiO_3$ film.

Comment 7: Figures 3 and the text mention the correlation between strain and vortices, but the spatial distribution statistics of the strain quantitative values (such as histograms) are missing. In addition, thermal mismatch strain is the core mechanism, but if the strain can be tracked more directly with temperature changes through variable-temperature XRD or Raman, it will be more persuasive.

Response: In our work, geometrical phase analysis (GPA) is used to provide spatially resolved quantitative strain maps that directly visualize the spatial distribution of the strain quantitative values (local strain variations and gradients at the nanoscale are clearly visualized on Figure 3 of the manuscript), over fields of views of typically 50 nm. Such maps are the standard way to analyze and quantify strain in high-resolution STEM studies and already contain the full information on the local strain distribution.

Following your suggestion, we have performed temperature-dependent XRD to get deeper insights into the evolution of in-plane and out-of-plane lattice parameters of the superlattice. For θ-2θ scans (determination of the average out-of-plane lattice parameter of the superlattice), the 002 and 004 peaks of the superlattice and Si substrate, respectively, were recorded. For the determination of the average in-plane lattice parameters, the 200 and the 220 Bragg peaks of the superlattice and Si substrate, respectively, were recorded.

The measurements were performed in the temperature range of 25-400°C. The average in-plane and out-of-plane lattice parameters of the superlattice are shown in Figure R6 below.

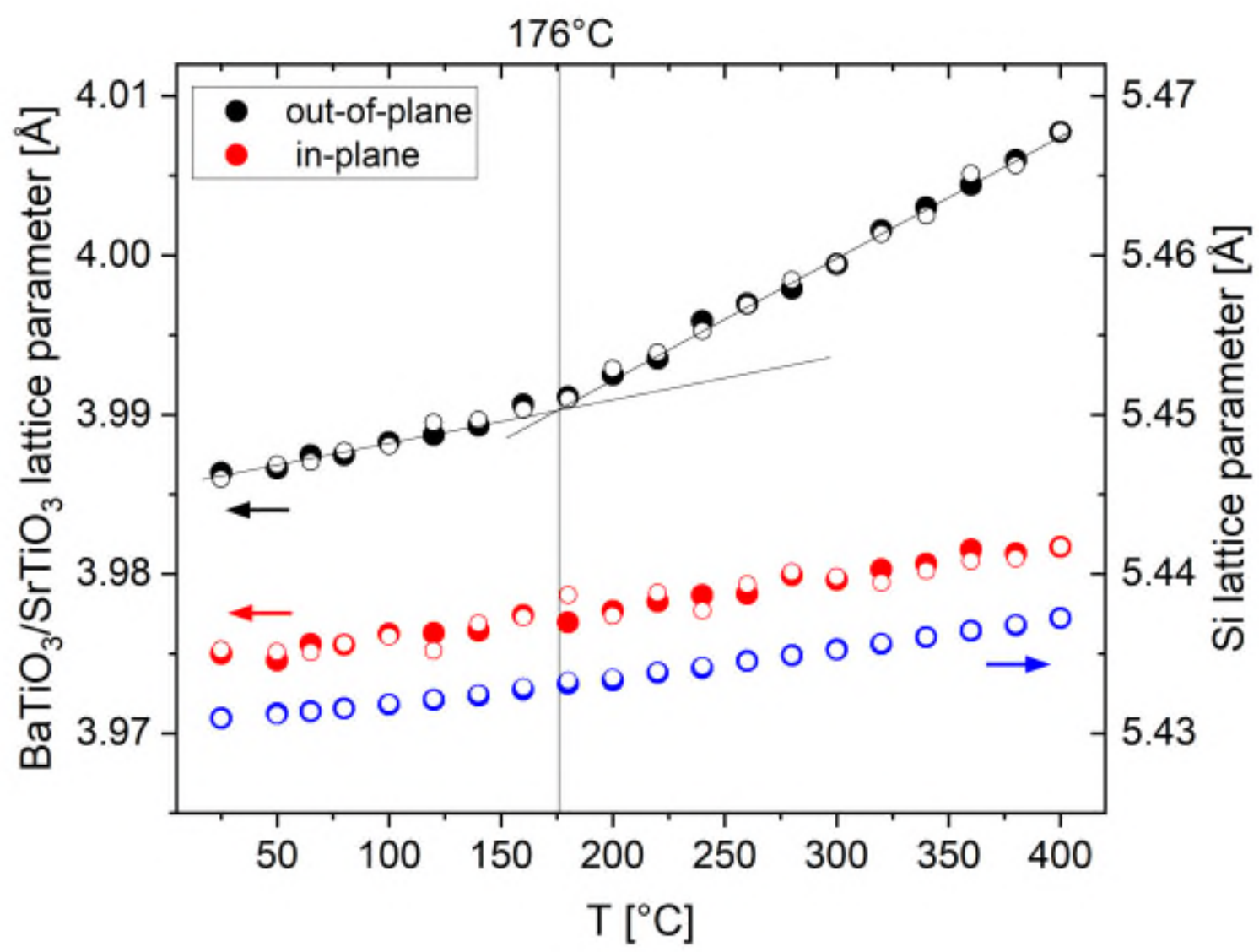

Figure R6**:** Temperature-dependent lattice parameters of the (12 unit-cells $BaTiO_3$/ 5 unit-cells $SrTiO_3)_{10}$ superlattice on silicon Si(001) and of the silicon substrate. Full and open circles correspond to lattice parameters extracted during sample heating and cooling, respectively.

The in-plane lattice parameter of the superlattice increases with temperature at the same rate as that of the silicon substrate, indicating that the film remains fully clamped over the investigated temperature range. The out-of-plane lattice parameter follows a similar thermal expansion behavior up to ~ 176 °C. Above this temperature, a clear change in slope is observed, indicating a phase transition. Notably, this transition temperature coincides with that determined from Raman spectroscopy (Fig. 5b) and with the temperature at which the polar vortices disappear (Fig. 5a), indicating a direct correlation between the structural evolution and the disappearance of the vortices.

Note that the larger thermal expansion rate above the phase transition (here at ~ 176 °C) is consistent with reports for $BaTiO_3$ ceramics (Y. He, *Thermochim. Acta* **419**, 135 (2004)).

Modifications to the manuscript:

- p. 11, line 264: Raman spectroscopy and XRD measurements on the same sample in the temperature range 300 - 500 K (Figure 5.b) and 300 – 673 K (Figure S11), respectively, reveal a structural phase transition in $BaTiO_3$. The intensity of the B_1 + E(LO+TO) at ~ 300 cm^{-1}, indicative of the $BaTiO_3$ tetragonality[69], vanishes at 440 K, which marks the ferroelectric-to-paraelectric phase transition, as shown by temperature-dependent Raman measurements performed on a *c*-oriented $BaTiO_3$ single crystal (Figure S5.c of the Supporting information). In XRD, a change in slope for the *c*-axis lattice parameter as a function of temperature (Figure S11) appears at 449 K (176 °C), in very good agreement with the Raman spectroscopy results, considering the different set-up/temperature measurements. The T_c of the superlattice is therefore in the range 440 – 450 K. Note that there is no hysteretic behavior of the lattice parameters upon heating/cooling.

Modifications to the Supporting Information:

- p. 10: We added Figure S11.

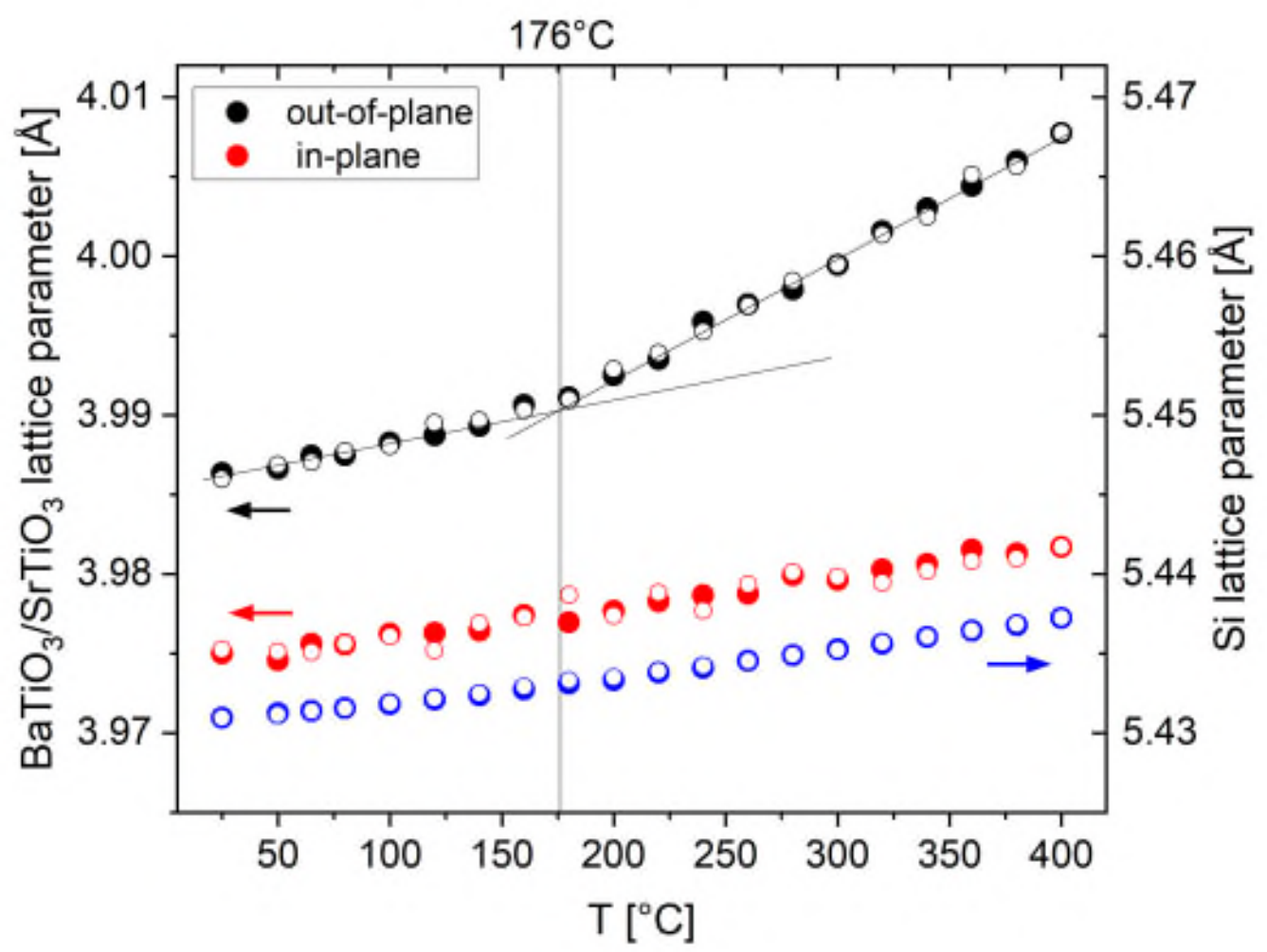

Figure S11: **Temperature-dependent lattice parameters of the (12 u.c. $BaTiO_3$/ 5 u.c. $SrTiO_3)_{10}$ superlattice on silicon Si (001) and of the silicon substrate.** Full and open circles correspond to lattice parameters extracted during sample heating and cooling, respectively.

Note: the larger thermal expansion rate above the phase transition (here at ~ 176 °C) is consistent with reports for $BaTiO_3$ ceramics (Y. He, *Thermochim. Acta* **419**, 135 (2004)).

Comment 8: The paper observed that the vortices are in the ground state and thermally stable, but did not explore their dynamic behavior (such as writing, erasing, flipping) under an applied electric field. The author is requested to explain this.

Response: The present work focuses on the stabilization and thermal robustness of polar vortices in $BaTiO_3$/$SrTiO_3$ superlattices on Si (the first ever report of this kind for this system and on silicon). The study of the dynamics and manipulation under external electric fields of these vortices is out of the scope of this paper.

Microscopic investigations of vortex dynamics under electric fields would require operando methods, such as in-situ biasing STEM, which represent a topic on its own and is not the scope of this paper.

As discussed in our response to Comment 6, macroscopic electrical measurements are currently hindered by large leakage currents, which prevent the reliable application of electric fields across the superlattices. We are working on reducing the leakage currents by doping $BaTiO_3$.

Piezoresponse force microscopy has been performed but due to the size of each vortex (~3.5 nm), we do not have the resolution to study the vortices individually (resolution is limited by the tip size, typically 20 nm at the apex)

Comment 9: The paper states that the $SrTiO_3$ layer is polarized at the interface, which is the key to coupling. It is suggested to conduct a more quantitative analysis of the size and depth distribution of the polarization within the $SrTiO_3$ layer.

Response: Thank you for this suggestion. We have analyzed both the magnitude and direction of the Ti displacements in the $SrTiO_3$ and $BaTiO_3$ layers from HAADF-STEM images (with [100] zone axis). A representative one is shown in Figure R7a below. The corresponding polar plot

(Figure R7b) indicates that the Ti displacements in the analyzed region have a preferential orientation to the left, which coincides with the propagation direction of the wave-like polarization. In the $SrTiO_3$ layers, the Ti displacements follow the same orientation; their magnitude (which can reach up to 20 pm for some unit cells) is smaller than in the $BaTiO_3$ layers and depend on the location laterally.
We have analyzed the average Ti displacement in each monolayer across the thickness of a $SrTiO_3$ layers and parts of the adjacent $BaTiO_3$ layers (Figure R7c). The $SrTiO_3$ layers show an average enhanced polarization at the interfaces to the adjacent $BaTiO_3$ layers as compared to the center regions. Note, however, that this plot shows an average over a lateral width of 16 nm, where strong variations occur laterally depending on the "location" on the polar wave.

Both the polar plot and the average values analysis confirm that $SrTiO_3$ is significantly polarized and coupled to the polarization of $BaTiO_3$ at interfaces (the coupling is clearly seen on the STEM images).

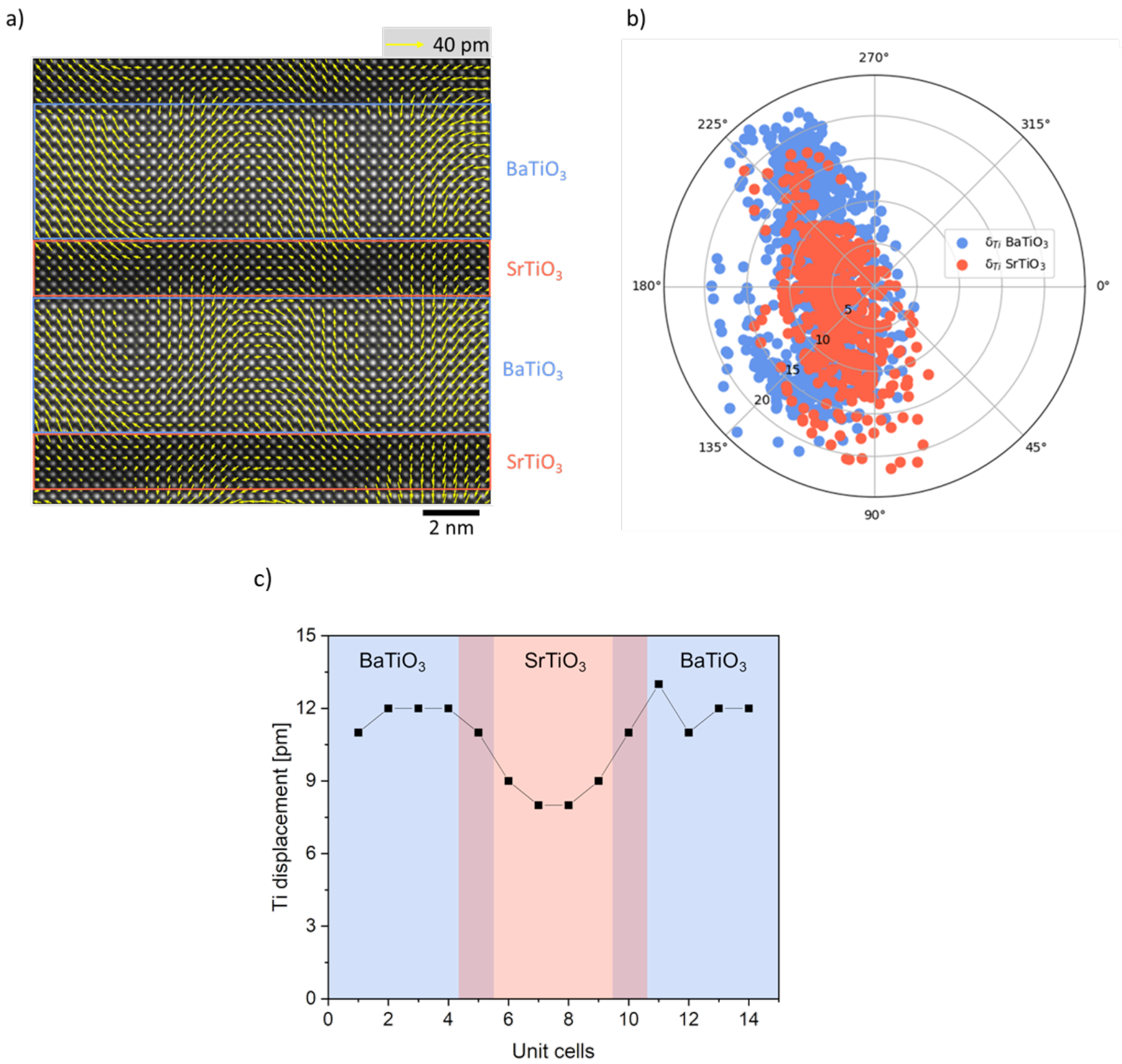

Figure R7: a) HAADF-STEM image in [100] zone axis overlaid with Ti-displacement vectors shown as yellow arrows. Blue and red boxes indicate the $BaTiO_3$ and $SrTiO_3$ layers. b) Polar plot of the direction and magnitude of the Ti-displacement extracted from the $BaTiO_3$ (two blue boxes in a)) and $SrTiO_3$ layers (two red boxes in a)). Each dot is representative of the Ti displacement in a unit cell in the STEM image shown in a). c) Average Ti displacement in each monolayer across the thickness of a $SrTiO_3$ layer (red) and parts of the adjacent $BaTiO_3$ layers (blue). The interface regions are indicated by the mixed color.

Modification to manuscript:

- p.8, line 200: "$SrTiO_3$ in the superlattice is polarized at the interfaces with $BaTiO_3$ layer and show in-plane and out-of-plane components (Figure 2 and Figures S8 and S9 of the Supporting

Information). The direction of Ti-displacement in the $SrTiO_3$ layers follows the one in the adjacent $BaTiO_3$ layers. The $SrTiO_3$ unit cells are more polarized at the interfaces to the adjacent $BaTiO_3$ layers than inside the layers.

Modification to the Supporting Information:

- Page12: We have added Figure S9 showing an analysis of the polarization directions and amplitudes in $BaTiO_3$ and $SrTiO_3$ and the corresponding STEM image.

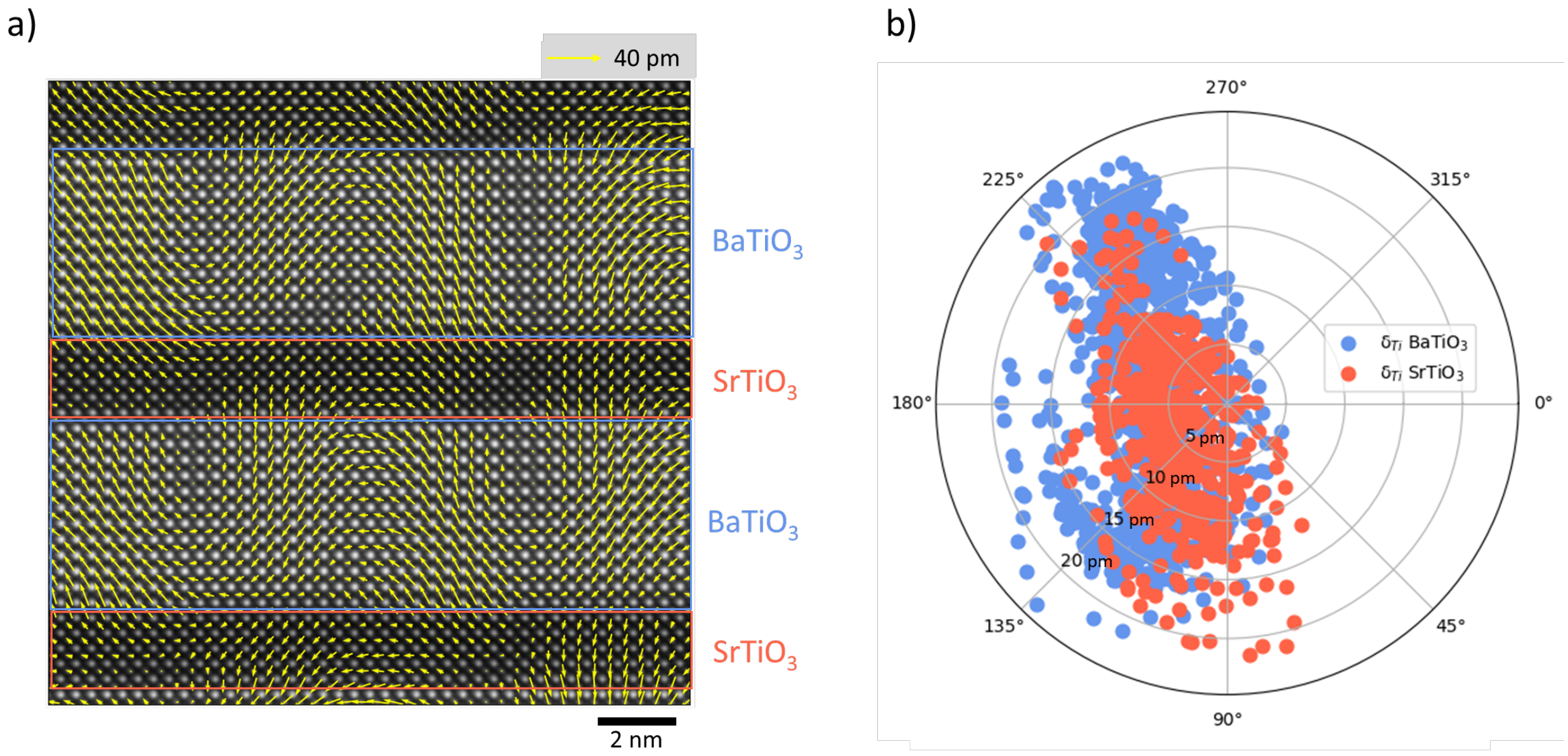

Figure S9: Analysis of the Ti displacement in a (12 u.c. $BaTiO_3$ / 5 u.c. $SrTiO_3$)$_{10}$ superlattice on Si (001). a) HAADF-STEM image in [100] zone axis overlaid with Ti-displacement vectors shown as yellow arrows. Blue and red boxes indicate the $BaTiO_3$ and $SrTiO_3$ layers. b) Polar plot of the direction and magnitude of the Ti-displacement vector extracted from the $BaTiO_3$ (two blue boxes in a)) and $SrTiO_3$ layers (two red boxes in a)). Each dot is representative of the Ti displacement in a unit cell in the STEM image shown in a).

Comment 10: It is recommended to consider using EELS or EDS line scans to verify the sharpness of Ba and Sr elements at the interface, excluding inter-diffusion, which can further support the "sharp interface" conclusion and relate it to the uniformity of performance.

Response: Thank you for raising this point. $Ba_xSr_{1-x}TiO_3$ form a solid solution over the entire compositional range and interdiffusion at interfaces is indeed likely happening since the growth does not proceed in an ideal 2D mode.

We performed atomically resolved energy-dispersive X-ray spectroscopy (EDX) measurements across the $SrTiO_3$/$BaTiO_3$ interfaces. Elemental maps of Ba K, Sr K, Ti L, and O K signals were recorded (Figure R8).

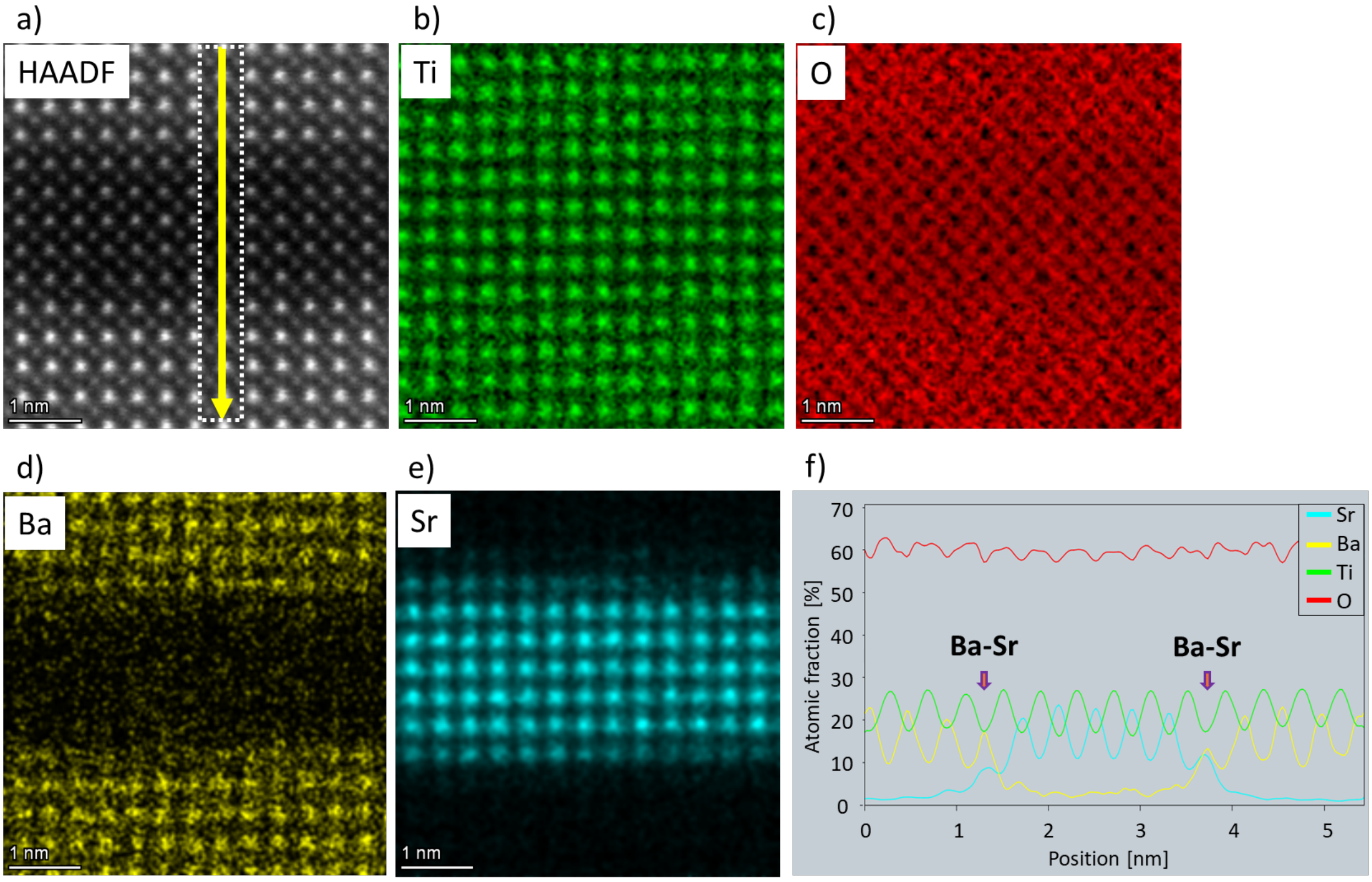

Figure R8: a) HAADF-STEM image of the $BaTiO_3/SrTiO_3$ superlattice together with corresponding EDX elemental maps of b) Ti-L, c) O-K, d) Ba-K, and e) Sr-K. f) EDX line profiles extracted along the line indicated in panel a), showing the spatial distribution of the constituent elements across the $BaTiO_3/SrTiO_3$ interfaces.

The elemental maps and corresponding intensity line-profile show a homogeneous distribution of Ti and O throughout the superlattice (Figure R8.b,c). The Ba and Sr maps are shown in Figure R8.d,e. At both types of interfaces - $BaTiO_3$ to $SrTiO_3$ and $SrTiO_3$ to $BaTiO_3$ - the first unit cell exhibits detectable signals from both Ba and Sr, indicating a mixed A-site composition. Such interfacial mixing is expected during our MBE growth, since the growth does not proceed via perfectly terminated two-dimensional monolayers at the atomic scale; the layers were grown by co-deposition of Ba and Ti for $BaTiO_3$ (and Sr and Ti for $SrTiO_3$) while the sample was continuously rotated during deposition to ensure uniform growth across the 2-inch Si wafer. Therefore, the growth was not monitored using RHEED oscillations to precisely terminate each individual monolayer. Consequently, when the deposition of the $SrTiO_3$ layer is stopped, the growth front is not necessarily atomically complete and may contain, moreover, both TiO_2 and SrO terminations. During the subsequent growth of $BaTiO_3$, Ba atoms are incorporated onto this non-ideal surface, naturally resulting in a mixed A-site composition over approximately one unit cell at the $SrTiO_3/BaTiO_3$ interface. The same holds true for the growth of $SrTiO_3$ on top of $BaTiO_3$ resulting in the intermixing at the $BaTiO_3/SrTiO_3$ interface.

The EDX line scan across the interfaces (Figure R8f) reveals a weak Ba signal within the $SrTiO_3$ layers, suggesting a possible minor content of Ba in $SrTiO_3$. However, the magnitude of this signal remains low and should be interpreted with caution. In thin superlattices with only five unit cells of $SrTiO_3$ sandwiched between $BaTiO_3$ layers, electron beam delocalization, probe broadening, and multiple scattering effects contribute to apparent elemental overlap in atomic-resolution EDX maps. In addition, the substantially high excitation energy of the Ba K edge results in weak X-ray intensity and low counting statistics, which introduces additional uncertainties in peak fitting and quantitative analysis. We did not use the Ba-L line as there is a strong overlap between the Ba–L

and Ti–K emission lines, which introduces too much uncertainty during background subtraction and peak deconvolution.

The weak Ba signal inside the $SrTiO_3$ layers is likely related to multiple scattering effects (note that no Sr is observed in the 12 u.c. of $BaTiO_3$ except at the interfaces).

Based on the EDX analysis, we have revised our discussion in the manuscript to mention the intermixing at interfaces.

Modification in the manuscript

- p. 5, line 129: "However, atomically resolved chemical maps obtained by energy-dispersive X-ray spectroscopy (EDX) reveal A-site cation (Ba, Sr) intermixing at each interface over one unit cell (Figure S1 of the Supporting Information). The intermixing is favored by a non-perfect 2D growth mode and MBE co-deposition growth (Ba and Ti for $BaTiO_3$, Sr and Ti for $SrTiO_3$) and therefore the absence of monolayer-precise growth termination."

- p. 5: Caption of Figure 1.b: we removed "showing sharp interfaces between the perovskite layers"

Modifications to Supporting Information:

p. 2: We have added the EDX analysis as Figure S1.

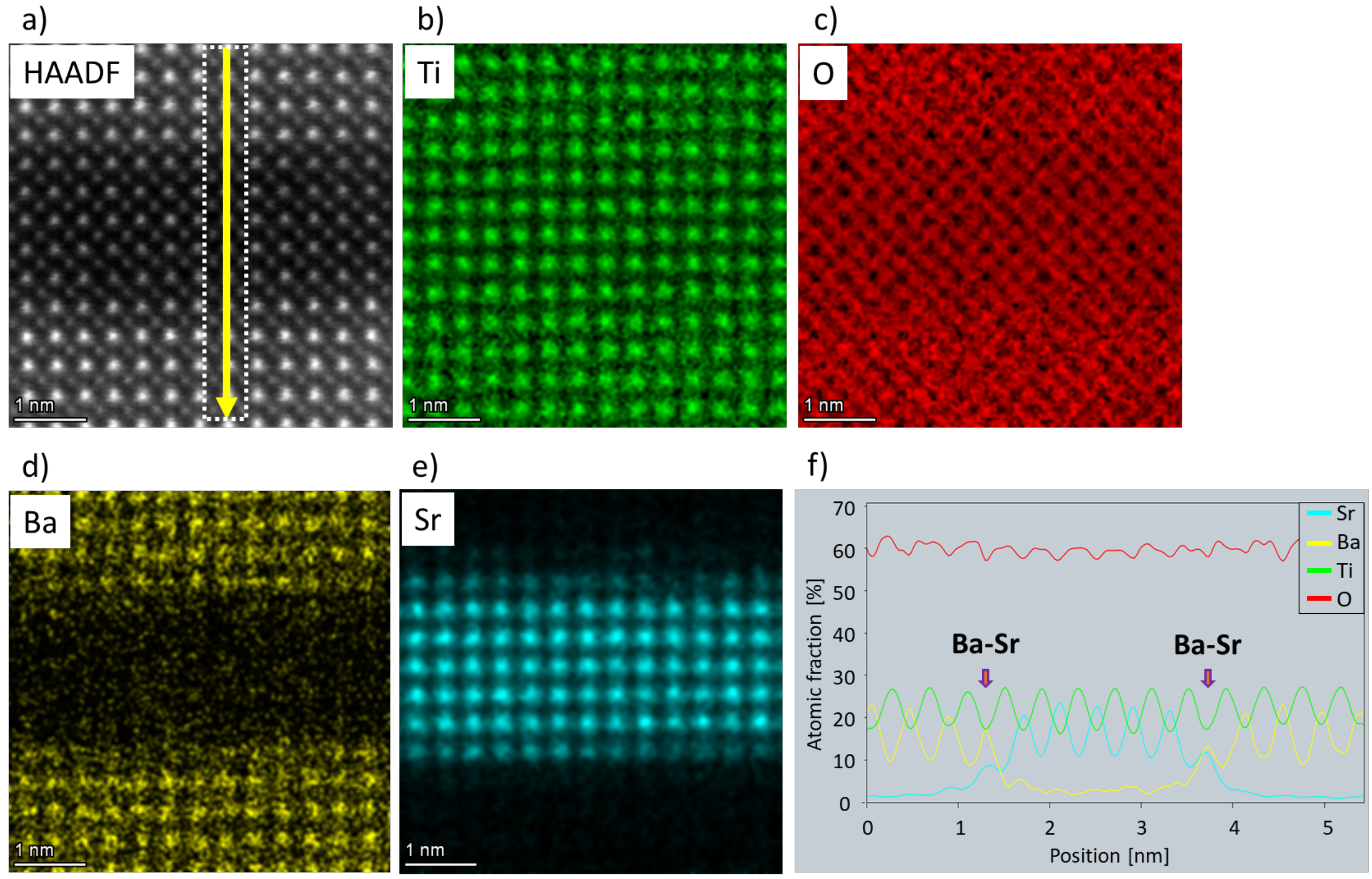

Figure S1: EDX analysis of a (12 u.c. $BaTiO_3$ / 5 u.c. $SrTiO_3$)$_{10}$ superlattice on Si (001). a) HAADF-STEM image together with corresponding EDX elemental maps of b) Ti-L, c) O-K, d) Ba-K, and e) Sr-K. f) EDX line profiles extracted along the line indicated in panel a), showing the spatial distribution of the constituent elements across the $BaTiO_3$/$SrTiO_3$ interfaces.

The HAADF STEM image of the analyzed region is shown in Figure S1.a. The elemental maps and corresponding intensity line-profile indicate a homogeneous distribution of Ti and O throughout the superlattice (Figure S1.b,c). The Ba and Sr maps are shown in Figure S1.d,e. At both types of interfaces - $BaTiO_3$ to $SrTiO_3$ and $SrTiO_3$ to $BaTiO_3$ - the first unit cell exhibits detectable signals from both Ba and Sr, indicating a mixed A-site composition. Such interfacial mixing is expected during our MBE growth, since the growth does not proceed via perfectly terminated two-dimensional monolayers at the atomic scale; the layers were grown by co-deposition of Ba and Ti for $BaTiO_3$ (and Sr and Ti for $SrTiO_3$) while the sample was continuously rotated during deposition to ensure uniform growth across the 2-inch Si wafer. Therefore, the growth was not monitored using RHEED oscillations to precisely terminate each individual monolayer. Consequently, when the deposition of the $SrTiO_3$ layer is stopped, the growth front is not necessarily atomically complete and may contain, moreover, both TiO_2 and SrO terminations. During the subsequent growth of $BaTiO_3$, Ba atoms are incorporated onto this non-ideal surface, naturally resulting in a mixed A-site composition over approximately one unit cell at the $SrTiO_3/BaTiO_3$ interface. The same holds true for the growth of $SrTiO_3$ on top of $BaTiO_3$ resulting in the intermixing at the $BaTiO_3/SrTiO_3$ interface.

The EDX line scan across the interfaces (Figure S1.f) reveals a weak Ba signal within the $SrTiO_3$ layers, suggesting a possible minor content of Ba in $SrTiO_3$. However, the magnitude of this signal remains low and should be interpreted with caution. In thin superlattices with only five unit cells of $SrTiO_3$ sandwiched between $BaTiO_3$ layers, electron beam delocalization, probe broadening, and multiple scattering effects contribute to apparent elemental overlap in atomic-resolution EDX maps. In addition, the substantially high excitation energy of the Ba K edge results in weak X-ray intensity and low counting statistics, which introduces additional uncertainties in peak fitting and quantitative analysis. We did not use the Ba-L line as there is a strong overlap between the Ba–L and Ti–K emission lines, which introduces too much uncertainty during background subtraction and peak deconvolution.

The weak Ba signal inside the $SrTiO_3$ layers is likely related to multiple scattering effects (note that no Sr is observed in the 12 u.c. of $BaTiO_3$ except at the interfaces).

Comment 11: The simulation in Figure 6 qualitatively agrees with the experiment, but lacks quantitative comparison of key parameters, such as vortex period, absolute value of polarization strength, and matching degree of strain size. Enhancing quantitative comparison can greatly enhance the credibility and predictive power of the model. The author is requested to make a supplement.

Response: For the quantitative comparison between experiment and phase-field simulations, we have summarized key parameters such as the vortex periodicity and relative polarization amplitudes in the $BaTiO_3$ and $SrTiO_3$ layers in Table R1 below. Absolute polarization values cannot be directly extracted from HAADF-STEM images, as this would require determination of the displacement of all atomic species, including oxygen, which is not visible in HAADF imaging. Instead, we quantify the Ti displacements within each unit cell, which is proportional to the polarization. This allows a meaningful comparison of polarization ratios between experiment and simulation. The HAADF-STEM image shown in Figure 2.b of the manuscript was analyzed.

Table R1: Comparison of key parameters from experiments and phase field simulations for effective lattice parameter a_{eff} = 3.99 Å. The abbreviation oop stands for out-of-plane and ip for in-plane. The polarization ratios correspond to the Ti displacement ratios.

	**Experiment**	**Simulation**
In-plane lattice parameter [Å]	3.982	3.99
Vortex periodicity [nm]	6.5 (RSM) - 7.0 (plan view STEM)	7.24
$P_{oop}(BaTiO_3)/P_{ip}(BaTiO_3)$	8.65	5.68
$P_{oop}(SrTiO_3)/P_{ip}(SrTiO_3)$	1.78	0.55
$P_{oop}(BaTiO_3)/P_{oop}(SrTiO_3)$	2.61	5.43
$P_{ip}(BaTiO_3)/P_{ip}(SrTiO_3)$	0.54	0.53

Phase-field simulations performed with an effective substrate lattice parameter a_{eff} = 3.99 Å provide the best agreement with the experimental observations (including the periodic alignment along the <110> directions). Simulations with a_{eff} = 3.97 Å result in a more isotropic polarization pattern (Figure 7a). The experimentally determined average in-plane lattice parameter is of 3.980 Å (XRD), a value close to 3.99 Å. The discrepancy between the experimental (3.980 Å) and simulated (3.99 Å) lattice parameters likely arises from the simplified boundary conditions inherent to the phase-field model and from local strain inhomogeneities present in the real heterostructure. In this context, the effective lattice parameter should be regarded as a phenomenological parameter that captures the averaged elastic and electrostatic state of the superlattice rather than the exact measured in-plane lattice constant.

Overall, the quantitative agreement in vortex periodicity and the relative polarization distribution between layers supports the predictive capability of the model. The vortex periodicity obtained from simulations (7.24 Å) shows a good agreement with experimental values extracted from STEM analysis (in the range 6.4 – 7.5 nm for different cross views and ~7.0 nm from planar view analysis).

Regarding the polarization components in the $BaTiO_3$ layers, experiments and simulation agree reasonably. However, the ratios of the out-of-plane components $P_{oop}(SrTiO_3)/P_{ip}(SrTiO_3)$ (experiment >1; simulation <1) and $P_{oop}(BaTiO_3)/P_{oop}(SrTiO_3)$ (experiment 2.61; simulation 5.43) indicate that the simulation underestimates the out-of-plane polarization component in the $SrTiO_3$ layers. This suggests that the simulation does not fully capture the magnitude of the induced polarization in the $SrTiO_3$ layers. In contrast, the relative in-plane polarization between $BaTiO_3$ and $SrTiO_3$ agrees very well between experiment and simulation ($P_{ip}(BaTiO_3)/P_{ip}(SrTiO_3)$ = 0.54 experimentally vs. 0.53 in simulation).

Modifications to manuscript:

- p.13, line 325: We added the sentence "A quantitative comparison of the key parameters between the experiment and phase field simulations is presented in Table S1 of the Supporting Information."
- p.14, Line 354: "The best agreement between experiments and simulations is obtained for an effective parameter of 3.99 Å (Table S1 of the Supporting Information)."

Modifications to the Supporting Information:

- p.15: We added the table discussed above as Table S1, with key parameters for the quantitative comparison between phase-field simulations and experiment as well as the associated discussion.

Quantitative comparison between experiment and simulation

Key parameters for the comparison between experiment and phase-field simulation are shown in Table S1.

Table S1: Comparison of key parameters from experiments and phase field simulations for effective lattice parameter a_{eff} = 3.99 Å. The abbreviation oop stands for out-of-plane and ip for in-plane. The polarization ratios correspond to the Ti displacement ratios.

	**Experiment**	**Simulation**
In-plane lattice parameter [Å]	3.982	3.99
Vortex periodicity [nm]	6.5 (RSM) - 7.0 (plan view STEM)	7.24
$P_{oop}(BaTiO_3)/P_{ip}(BaTiO_3)$	8.65	5.68
$P_{oop}(SrTiO_3)/P_{ip}(SrTiO_3)$	1.78	0.55
$P_{oop}(BaTiO_3)/P_{oop}(SrTiO_3)$	2.61	5.43
$P_{ip}(BaTiO_3)/P_{ip}(SrTiO_3)$	0.54	0.53

Phase-field simulations performed with an effective substrate lattice parameter a_{eff} = 3.99 Å provide a good agreement with the experimental observations (including the periodic alignment along the <110> directions). Simulations with a_{eff} = 3.97 Å result in a more isotropic polarization pattern (see Figure 7a of the manuscript). The experimentally determined average in-plane lattice parameter is of 3.982 Å (XRD), a value close to 3.99 Å. The discrepancy between the experimental (3.982 Å) and simulated (3.99 Å) lattice parameters likely arises from the simplified boundary conditions inherent to the phase-field model and from local strain inhomogeneities present in the real heterostructure.

Overall, the quantitative agreement in vortex periodicity and the relative polarization distribution between layers supports the predictive capability of the model. The vortex periodicity obtained from simulations (7.24 Å) shows a good agreement with experimental values extracted from STEM analysis (in the range 6.4 – 7.5 nm for different cross views and ~7.0 nm from planar view analysis).

Regarding the comparison of the relative polarization distributions, the experimental values are based on the relative Ti displacements within each unit cell determined from HAADF, which are proportional to the local polarization. This enables a consistent evaluation of polarization ratios between experiment and simulation. Overall, good agreement is found for the relative in-plane polarization between $BaTiO_3$ and $SrTiO_3$ layers. The ratios involving the out-of-plane components indicate that the simulations underestimate the magnitude of the induced out-of-plane polarization in the $SrTiO_3$ layers compared to experiment, while the polarization in $BaTiO_3$ is reproduced reasonably well.

Comment 12: The paper assumes that the vortex tube is infinitely extended. In actual finite-sized devices or regions with dislocations (such as Figure 1a), how does the vortex tube terminate? Will it form topological defects (such as Bloch points)? This is important for understanding the non-uniformity of device micro-region performance, and it is recommended to make a brief discussion.

Response: As discussed in our responses to Comments 1 from Reviewers 1 and 2, the polar vortex tubes in our $BaTiO_3/SrTiO_3$ superlattices are not infinitely extended but terminate at structural defects such as dislocations (also shown on the planar view in Figure 4 of the manuscript).

The periodic vortex tubes are locally disrupted by dislocation defects. In the plan-view HAADF-STEM data, the dislocation defects appear as regions of dark contrast. The periodic stripe pattern terminates or locally loses coherence in the regions where dislocations are present, indicating that these defects disturb the long-range lateral periodicity of the polar vortices.

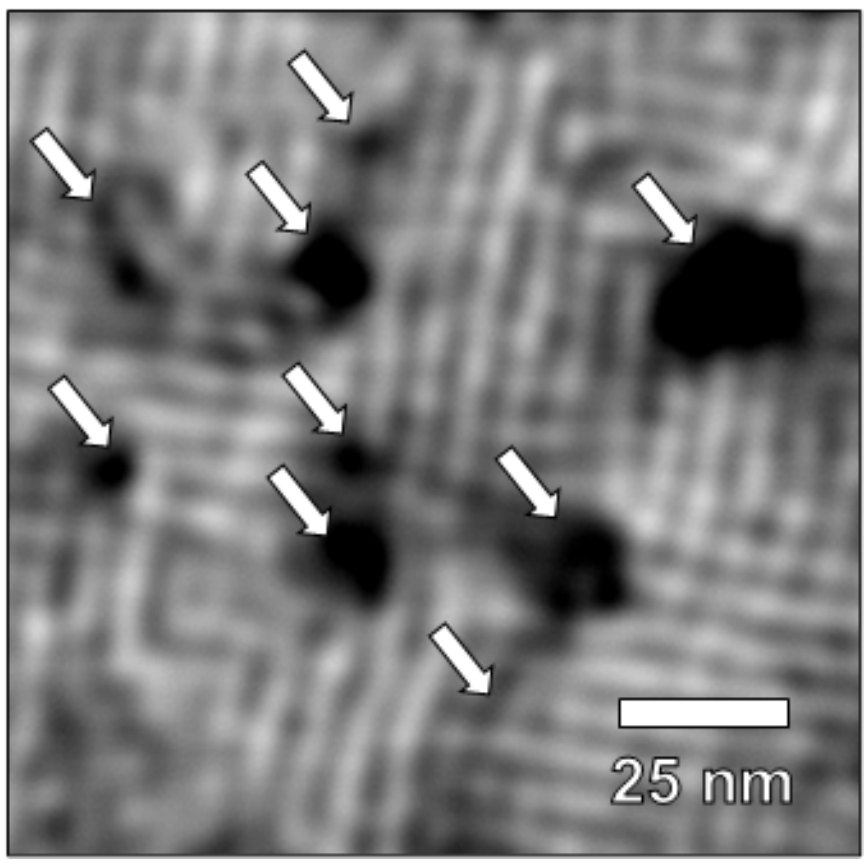

Plan-view STEM image of the $BaTiO_3$-$SrTiO_3$ superlattice containing dislocations. Regions of dark contrast marked by arrows are dislocations defects.

Strong and highly inhomogeneous strain fields around dislocations perturb the atomic columns, preventing an unambiguous determination of atomic positions and hindering the extraction of Ti-displacement vectors. Also, these dislocations alter the coupled electrostatic and elastic interactions that lead to the intrinsic polar ordering in the $BaTiO_3/SrTiO_3$ superlattices. As a result, the polar textures cannot be resolved in cross-sectional STEM near dislocations and therefore we can not conclude on the termination at the crossing of the vortices, whether these are topological defects such as Bloch points.

Phase-field simulations indicate that the termination of polar vortex tubes can occur through the formation of polar Néel-type disclinations (Figure R9).

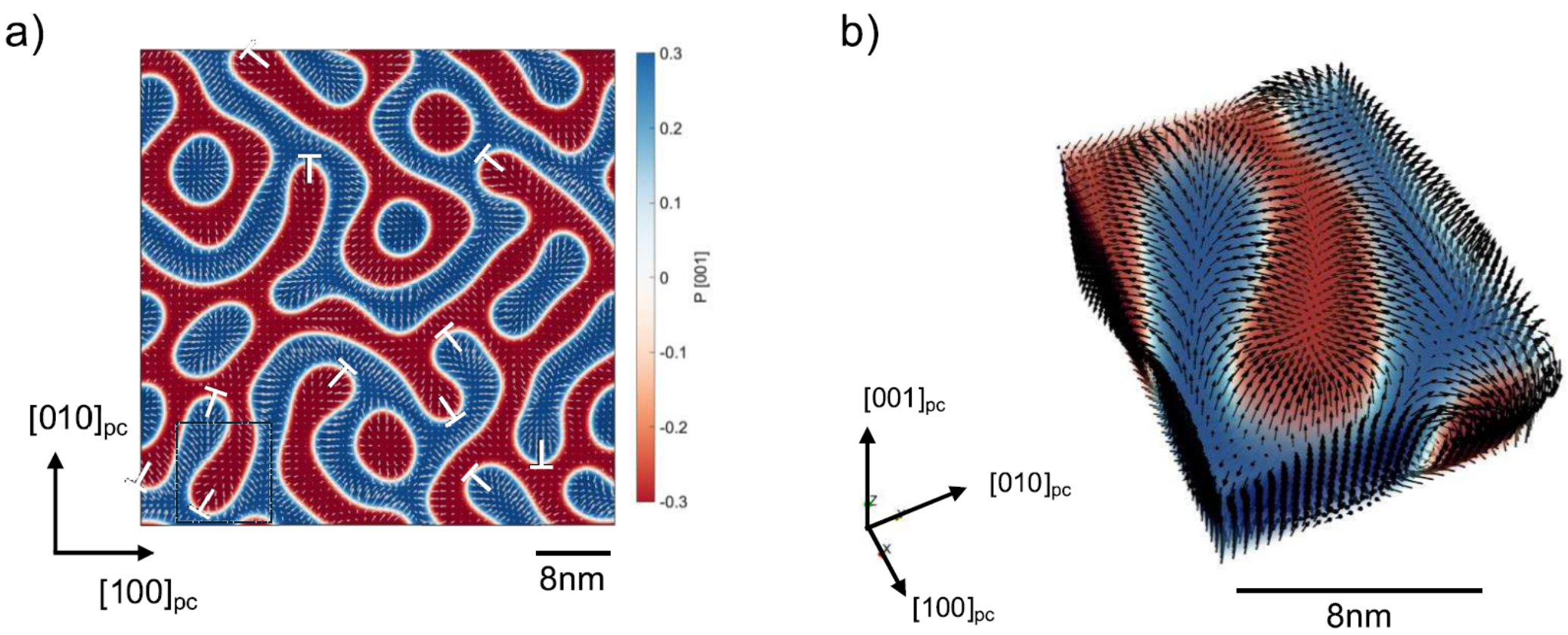

Figure R9: a) Slice projected onto (001) plane colored by the polarization along the [001] direction. Néel-type polar disclinations are highlighted. b) Magnified view of a representative Néel-type polar disclination.

Modification to the manuscript:

- p.7, line 179: "Due to the strong and highly inhomogeneous strain fields around dislocations, the atomic positions and therefore the Ti-displacement vectors can not be precisely determined in their vicinity."
- p.10, line 239: "The periodic vortex tubes are disrupted by dislocation defects which appear as regions of dark contrast in Figure 4.a. The periodic stripe pattern terminates or locally loses coherence in the regions where dislocations are present, indicating that these defects disturb the long-range lateral ordering of the polar vortices."
- p. 13, line 327: "Phase-field simulations indicate that the termination of polar vortex tubes can occur through the formation of polar Néel-type disclinations (Figure S13 of the Supporting Information)."

Modification to the Supporting Information file:

p. 17: we added Figure S12

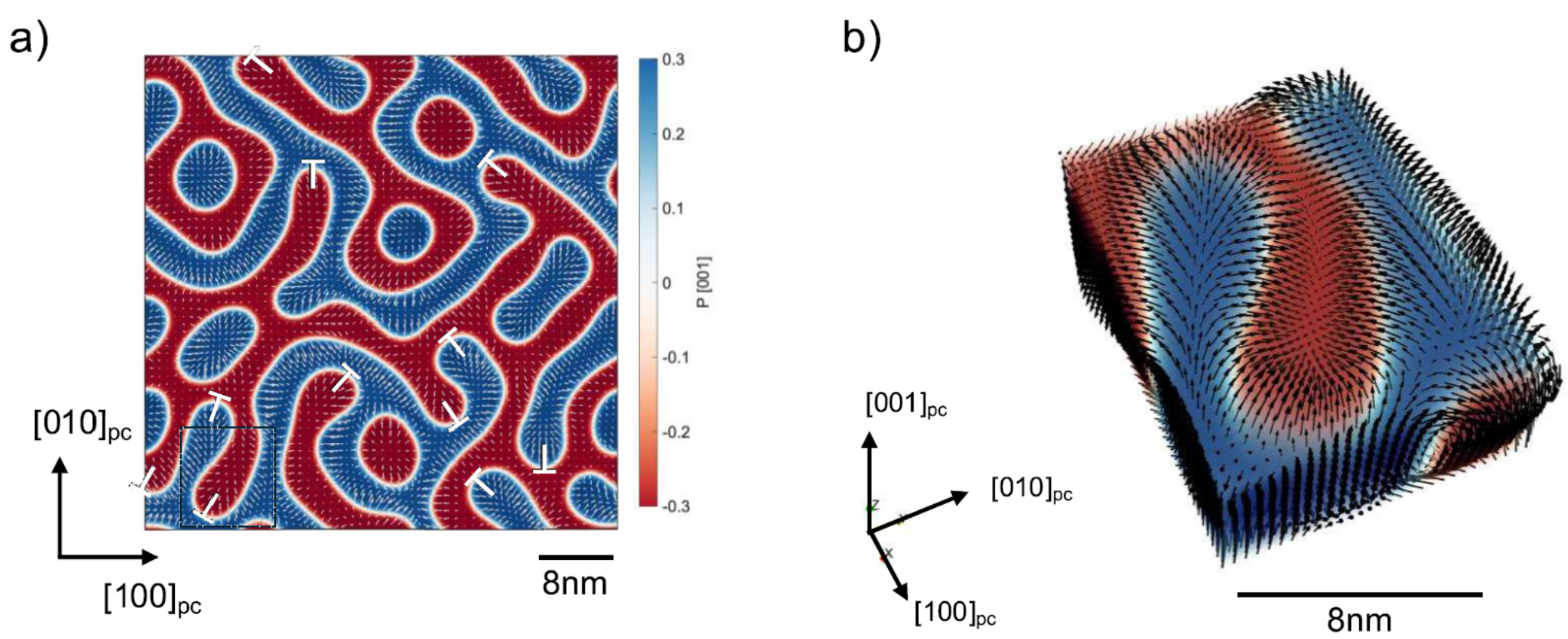

Figure S12: Phase field simulation of a (12 u.c. $BaTiO_3$/ 5 u.c. $SrTiO_3$)$_{10}$ superlattice on silicon Si (001). a) Slice projected onto (001) plane colored by the polarization along the [001] direction. Néel-type polar disclinations are highlighted. b) Magnified view of a representative Néel-type polar disclination.

Comment 13: Some pictures in the paper are not easy to understand, for example: Figure 1e: the intensity of the satellite peak is weak. It is suggested to add arrows or clear labels in the figure, or provide an enhanced contrast version in the supplementary figure.

Response: We have kept the figure as it is in the manuscript and, in the Supplementary file, we have added the figure with magenta arrows that point at the satellites.

Modification to the manuscript

p.6, line 148: **"**Each superlattice reflection is accompanied by in-plane satellite peaks indicating a lateral long-range ordering of ferroelectric domains along the <110>$_{BaTiO3}$ directions with a period of ~ 6.5 nm (Figure 1.e and Figure S6.e,f of the Supporting Information)."

Modifications to the Supporting Information file:

- p.8 and 9: We reorganized Figure S6 to add Figures S6.e,f which highlight the satellites peaks originating from the lateral periodicity of the polar domains.

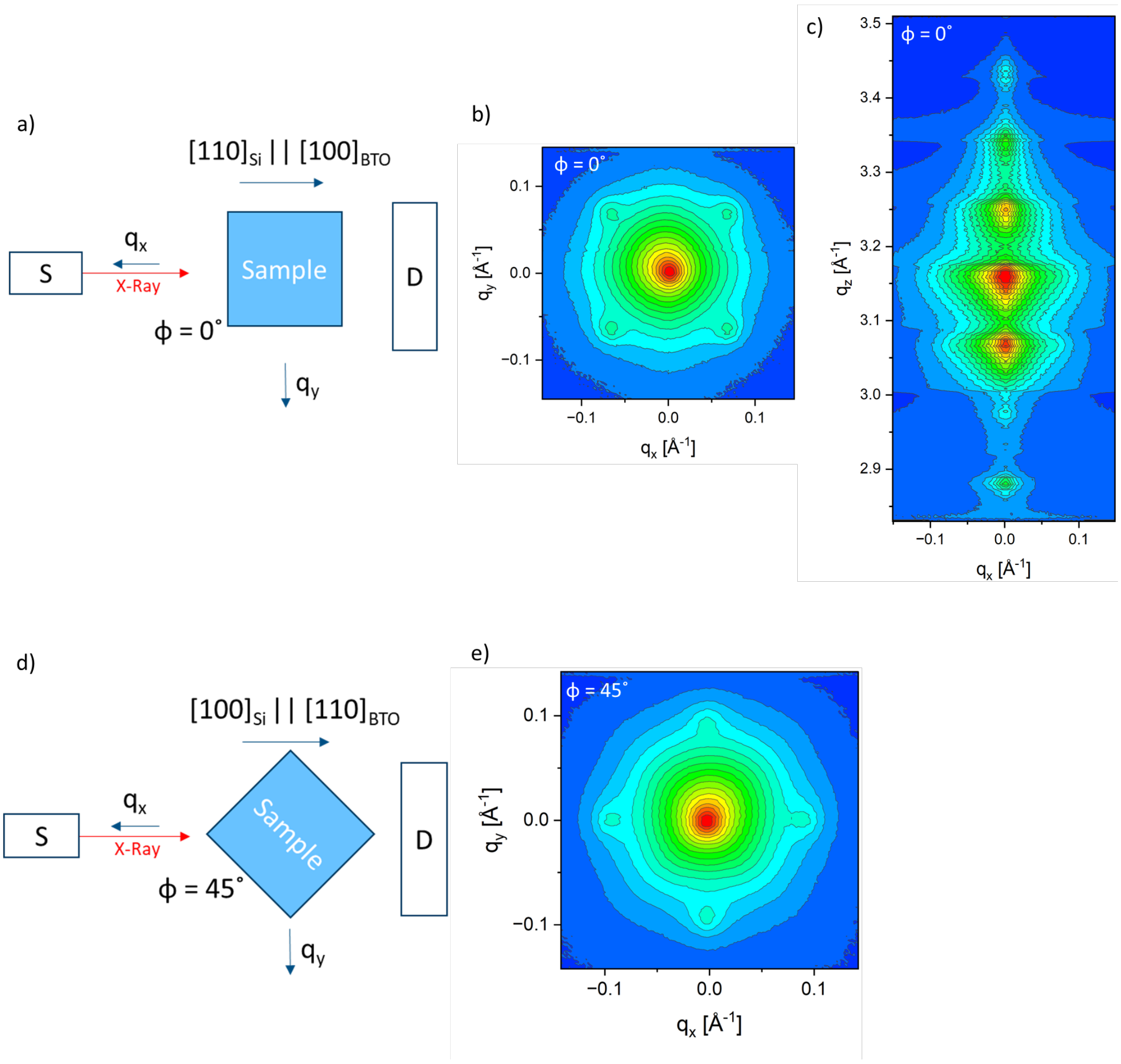

f)

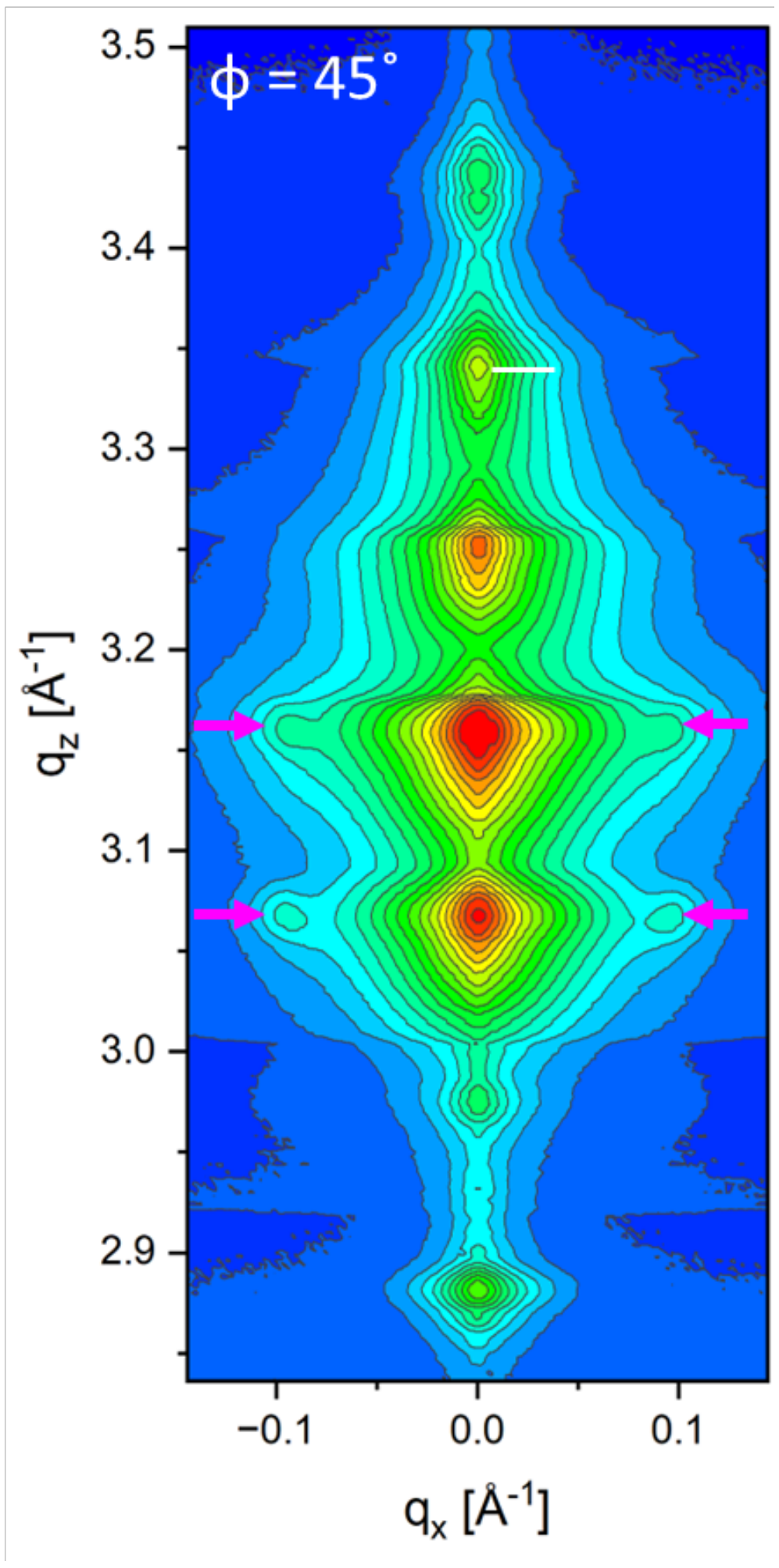

Figure S6: Synchrotron-radiation X-ray diffraction reciprocal space maps on a (12 u.c. $BaTiO_3$ / 5 u.c. $SrTiO_3)_{10}$ superlattice on Si (001). a) Schematic of the sample orientation with respect to the X-ray beam at ϕ = 0°. b, c) Reciprocal space maps recorded with ϕ = 0°, in the q_x-q_y plane for b) and q_x-q_z plane for c). d) Schematic of the sample orientation with respect to the X-ray beam at ϕ = 45°. e, f) Reciprocal space maps recorded with ϕ = 45° in the q_x-q_y plane for e) and in the q_x-q_z plane for f). Magenta arrows highlight the satellite peaks arising from the lateral polar order. Reciprocal space maps shown in the main text were recorded with ϕ = 45°.

Comment 14: Figure 5a: multiple RSM diagrams are densely arranged, and the satellite peaks (marked *) in the line spectrum are not obvious. It is recommended to consider re-arranging or displaying the key temperature RSMs in a larger size separately.

Response: We have rearranged Figure 5 and removed the line profiles extracted from the RSMs (they are not presented in the Supporting Information file). We increased the font of the labels on the axis and tick labels for better visibility.

Modifications to Manuscript:

- p.12: Figure 5 has been rearranged and improved for better readability.

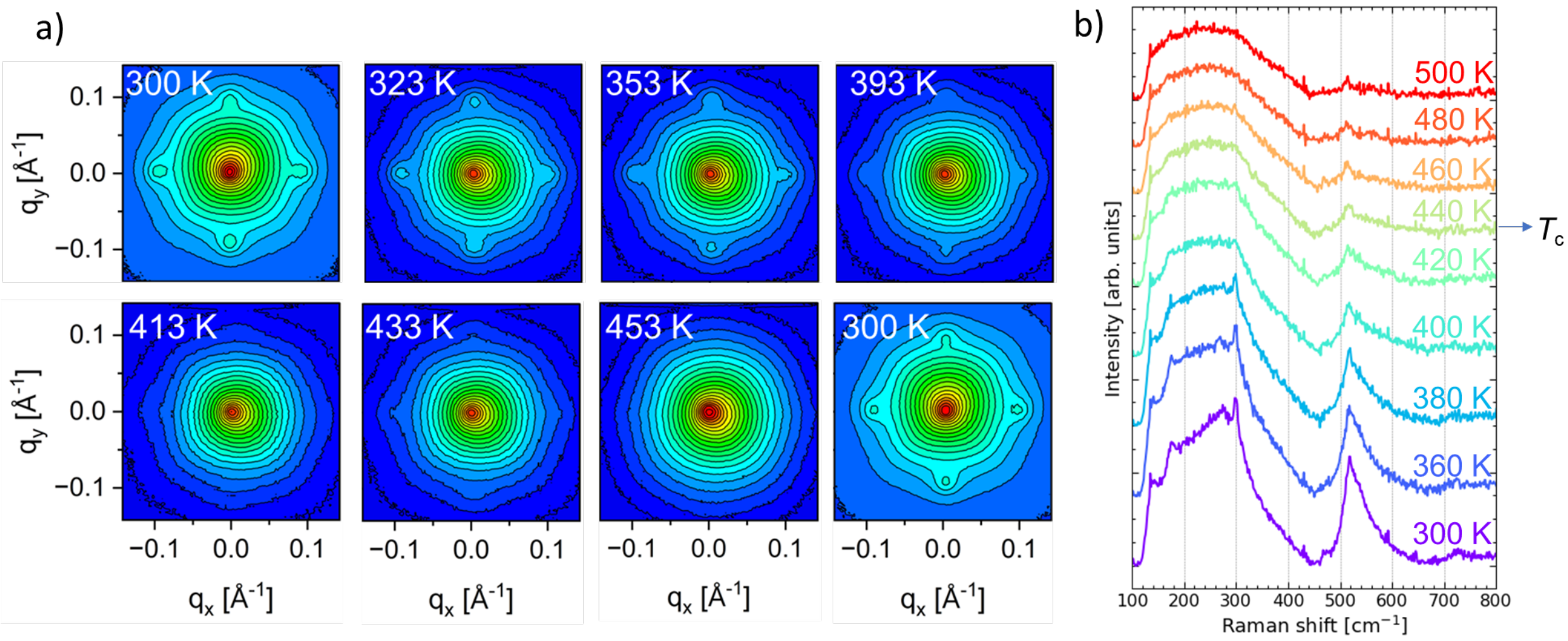

Figure 5: Temperature dependence of the long-range order of polar vortices in a (12 u.c. $BaTiO_3$/ 5 u.c. $SrTiO_3$)$_{10}$ superlattice on silicon Si(001). a) Synchrotron-based reciprocal space maps recorded at different temperatures. The corresponding line profiles at $q_y = 0$ along q_x are presented in Figure S10 of the Supporting Information. b) Temperature dependent Raman spectra showing the disappearance of the Raman modes indicative of tetragonal $BaTiO_3$ at a temperature of ~ 440 K**.** The indexed Raman spectrum recorded at 300 K is presented in Figure S5a of the Supporting information.

Comment 15: Figure 7: The dimensions of subgraphs (a) and (b) are too small, making it difficult to clearly observe the details of the structural evolution. It is recommended to enlarge them or provide enlarged images of the key areas.

Response: Thank you for your comment. We have enlarged the subgraphs (a) and (b) in Figure 7 and made full use of the page width.

Modifications to manuscript:

- p.16: Figure 7: We have enlarged the subgraphs to make full use of the page width.

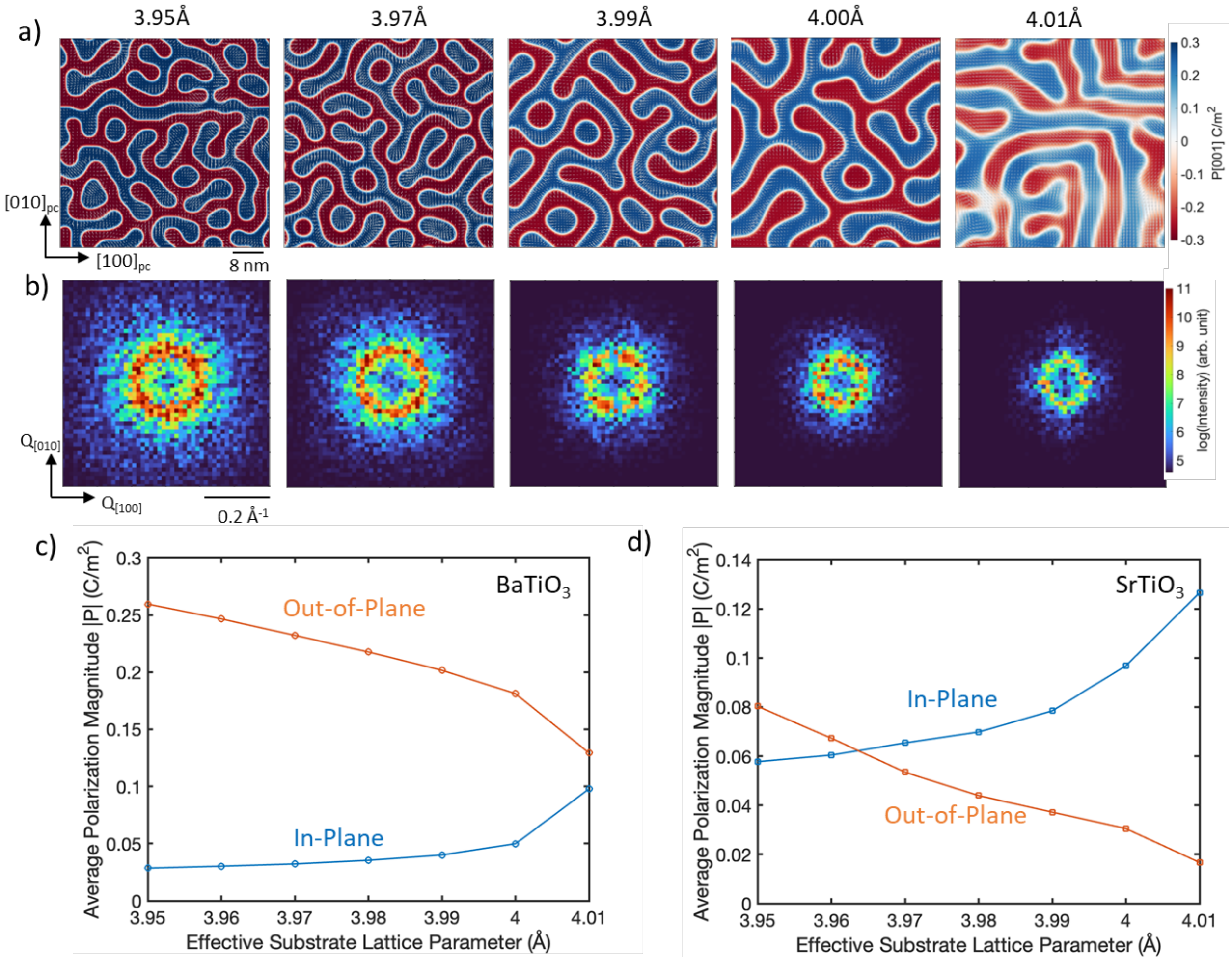

- p.13: Figure 6: The colormap of Fig. 6a was corrected. The original colormap was inverted with respect to the spontaneous polarization direction (red was supposed to be negative z polarization but instead it was positive z due to different conventions between visualization software). The arrows in figure c) were changed from black to gray to be more visible.

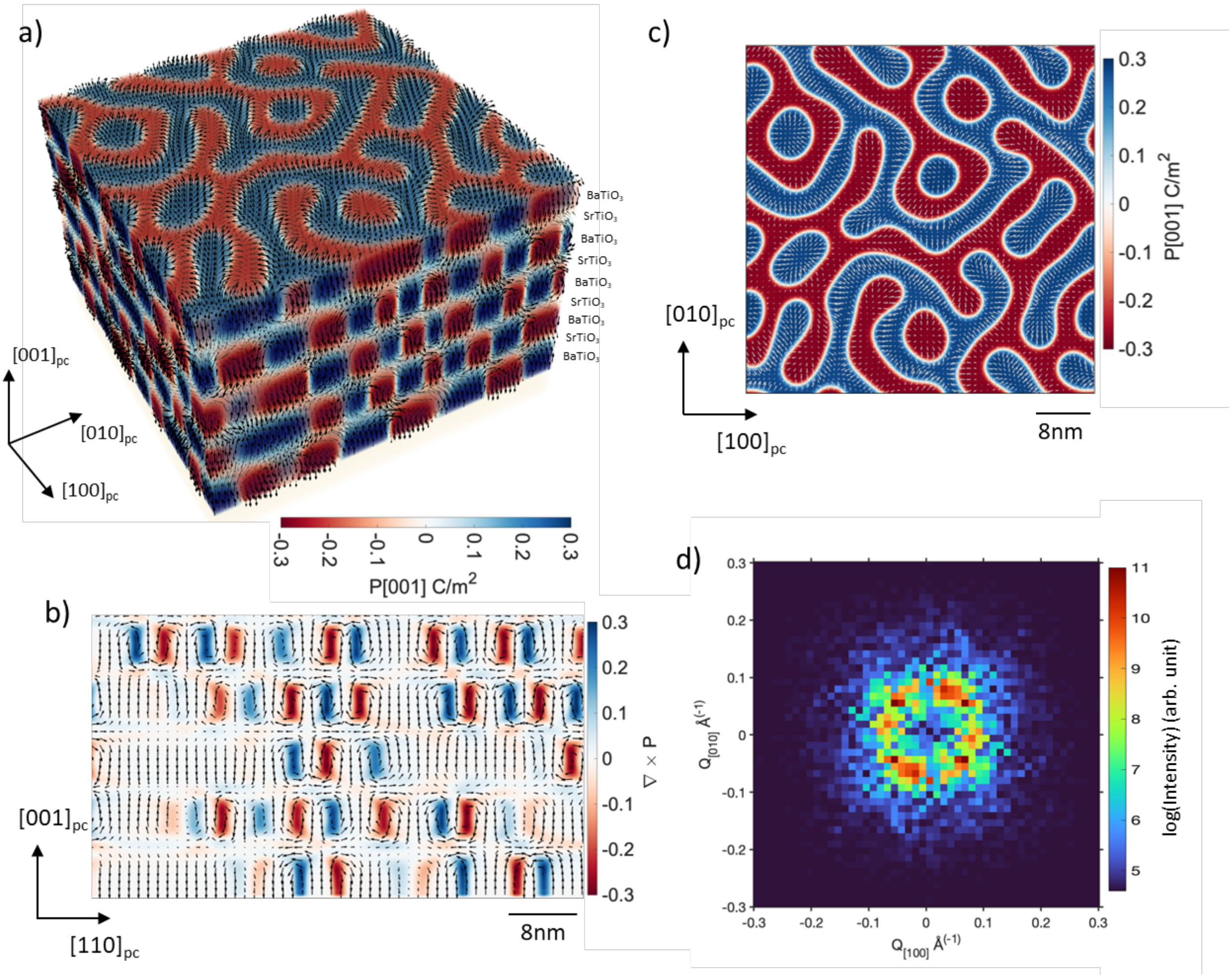

Figure 6: Phase-field simulations of a (12 u.c. $BaTiO_3$/ 5 u.c. $SrTiO_3$)$_{10}$ superlattice on silicon Si(001). a) Three-dimensional rendering of the polarization distribution. b) Two-dimensional slice of the polarization projected onto the (110) plane showing the curl of the polarization. c) Slice projected onto the (001) plane colored by the polarization along the [001] direction. d) Fast Fourier transform (FFT) of the (001) polarization slice showing distinct peaks corresponding to preferential ordering along the <110> directions.

Comment 16: The "Methods" section can be more concise and focus on the key points: The description of the FIB preparation process is overly detailed (occupying nearly two pages), and it repeats similar steps for different devices. It is recommended to significantly simplify it, merge the common steps, and use the saved space for in-depth discussion sections.

Response: We do think that the method section should describe precisely how the experiments were performed (here the FIB lamellae preparation) and give to the readers enough information to repeat the experiments. Since the lamellae were prepared on three different high-end equipment in Berlin, we thought that we should carefully describe the FIB preparation on the three tools. It is indeed detailed and it repeats indeed some similar steps, but some are different and we thought it is important to be precise. However, to respond favorably to your request, we have now shortened the description of the FIB preparation process.

Modifications to the manuscript:

p.21, line 534: "**Focused Ion Beam preparation of the lamellae.** The FIB preparation of the STEM lamellae were performed at three different locations. Cross-sectional and planar TEM lamellae were prepared using Ga^+-source FIB-SEM dual-beam systems (Thermo Fisher Helios

NanoLab G3 and Helios 600i). Specimens were oriented along the [110] or [100] zone axis of the Si substrate. The superlattice surface was protected by Electron Beam Induced Deposition (EBID) SiO_x or Pt followed by milling at 30 kV. Lamellae were extracted using standard lift-out procedures and mounted on Cu grids or half-grids.
To minimize bending and curtaining effects, some cross-sectional lamellae were prepared by the non-standard upside-down preparation technique, i.e. with an ion beam milling direction from the Si substrate to the surface[87]. The preparation was finalized with low energy milling steps at 5 and 2 keV (and down to 1 kV in some cases), minimizing the damage layer on the lamella sidewalls and achieving an estimated lamella thickness in the range of 20-40 nm.
For planar TEM specimens, samples were mechanically thinned from the substrate side to a thickness of approximately 40 µm and affixed to a copper half-ring. Electron-transparent windows, each approximately 30 × 30 μm^2 in size, were milled into the sample by FIB using stepwise reduction of the current (0.44 nA down to 10 pA) and voltage (30 kV down to 2 kV) and progressive increase of the milling angle (1° up to 6°)."

Reviewer #4

Remarks to the Author: I co-reviewed this manuscript with one of the reviewers who provided the listed reports. This is part of the Nature Communications initiative to facilitate training in peer review and to provide appropriate recognition for Early Career Researchers who co-review manuscripts.

Additional Modifications to the manuscript

We have revisited the XRD data used for determining the average out-of-plane and in-plane lattice parameters of the superlattice. The in-plane scan was performed without a monochromator, by mistake. To ensure accuracy, we remeasured the sample using a monochromator and high-resolution optics. The corrected in-plane lattice parameter is 3.980 Å (in-plane).

Modifications to the manuscript:

- p.5, line 137: "The average out-of-plane and in-plane lattice parameters of the superlattice are 3.992 Å and 3.982 Å, respectively."

Modifications to Supporting Information file:

- p.6: Figure S4.c) has been replaced by the scan performed with a monochromator.

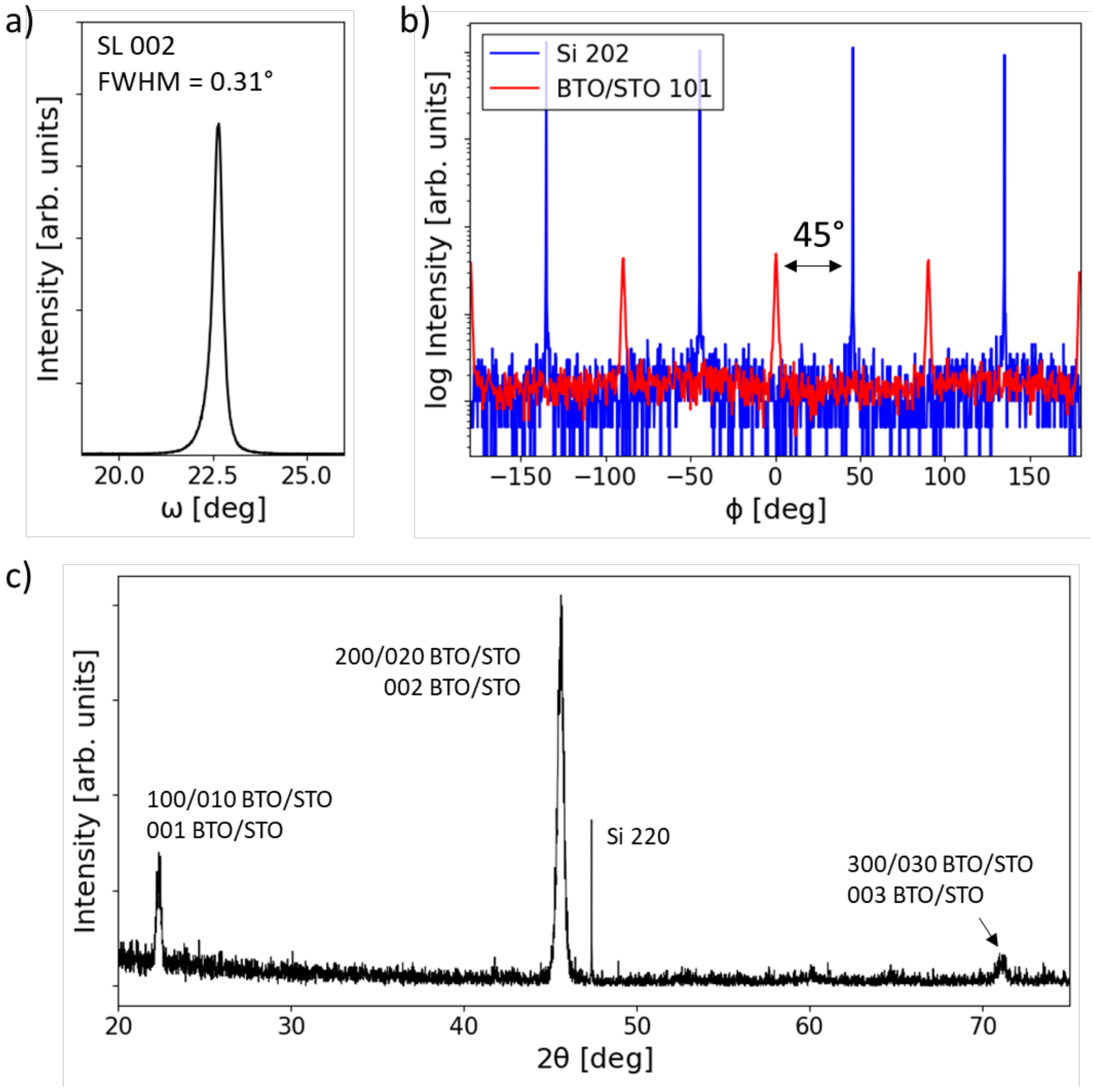
a)
SL 002
FWHM = 0.31°
Intensity [arb. units]
20.0
22.5
25.0
ω [deg]
b)
Si 202
BTO/STO 101
log Intensity [arb. units]
45°
−150
−100
−50
0
50
100
150
ϕ [deg]
c)
Intensity [arb. units]
200/020 BTO/STO
002 BTO/STO
100/010 BTO/STO
001 BTO/STO
Si 220
300/030 BTO/STO
003 BTO/STO
20
30
40
50
60
70
2θ [deg]

REVIEWER COMMENTS

We thank the reviewers for their thorough reviews, questions and comments. We appreciate their constructive criticism and suggestions.

We addressed the new comments raised and respond below.

Reviewer #1

All my concerns have been addressed in the revisions.

We thank Reviewer 1 for his/her positive evaluation of our manuscript.

Reviewer #2

The authors have carefully addressed the comments and resolved several issues. However, a few statements remain insufficiently supported or internally inconsistent and should be revised.

Comment 1: The authors describe the vortex periodicity as " reversible and non-hysteretic" with temperature. The return of the satellite peak position to its original position after cooling supports this reversibility, but the authors claim they cannot provide complete satellite peak measurements for the entire cooling process. A system may exhibit substantial hysteresis during heating and cooling while still recovering its initial room-temperature configuration. The author should revise the relevant descriptions.

Response: We thank the reviewer for this comment. We agree that the statement is not based on experimental data and that a hysteresis can not be excluded regarding the periodicity of the vortices. We have therefore removed the sentence "Hence, the change in periodicity with temperature is reversible and non-hysteretic."

Modification to the manuscript:

- p.10, line 260: We removed "Hence, the change in periodicity with temperature is reversible and non-hysteretic."
- p.11, line 262: We added "As we do not have intermediate data upon cooling down for the vortex periodicity, we can not conclude whether the vortex formation is hysteretic or not with temperature.

Comment 2: The PFM results do not provide sufficient evidence that the vortices are unwound under an applied bias. PFM resolution (~20 nm) is much larger than the vortex diameter (~3.5 nm), and the authors also report significant electrostatic charging and strong leakage. Therefore, the butterfly shape in the amplitude signal, 180° phase change, and stable written contrast may include electrostatic, charge-injection, ionic, electrochemical, and piezoelectric contributions. The ±6 V written regions only demonstrate a persistent bias-induced contrast, not vortex unwinding. The statement "it is possible to unwind vortices" should therefore be revised.

Response: We thank the reviewer for this comment. We agree with it. We have removed the sentence "These experiments suggest that it is possible to unwind vortices under an applied bias and that a remanent polarization can be stabilized locally" and rewrote this section of the text to comment more on the amplitude signal and the possible other origins for the contrast (with reference 86 added – N. Balke et al., ACS Nano (2015)). We added that we can not conclude on the unwinding of the vortices.

Modification to the manuscript:

p. 19: PFM results discussion (line 465 – 477):

- We removed the sentence "These experiments suggest that it is possible to unwind vortices under an applied bias and that a remanent polarization can be stabilized locally."
- p.19, line 465: "The electrical behavior of the superlattices could not be investigated by macroscopic measurements due to large leakage currents in $BaTiO_3$. Switching spectroscopy PFM shows a butterfly shape in the amplitude signal (Figure S13.a,b of the Supporting Information), and a hysteretic phase with a change of 180 degrees, which is the typical response of a ferroelectric material. However, such a response can also be obtained in non-ferroelectric materials[86]. By scanning the sample surface with $\pm$ 6 V DC tip bias in adjacent regions (Figure S13.c,d of the Supporting Information), we observe a 180 degrees phase difference for regions poled with opposite biases. The amplitude signal shows a zero response at the interface regions, which could correspond to ferroelectric domain walls. However, the written regions exhibit a strong contrast in amplitude, which may originate from electrostatic, charge injection, ionic and/or electrochemical contributions[86], effects that are likely enhanced by the fact that the superlattice terminates with a $SrTiO_3$ layer. Hence, the PFM measurements do not allow to conclude on the ability to unwind the vortices under an applied electrical field."

Comment 2: Several inconsistencies between the manuscript and the response letter should be verified. The patterned top electrode is described as Pt in one place and W in another. In addition, the corrected in-plane lattice parameter is reported as 3.980 Å in the response letter but as 3.982 Å in the revised manuscript.

Response: We thank the reviewer for spotting these mistakes. The patterned top electrode was Pt. The metal of the top electrodes of the capacitors is not mentioned in the manuscript since we could not perform macroscopic measurements. There was indeed a mistake in the response to Reviewer 3, Comment 6.

The corrected in-plane lattice parameter is 3.982 Å, which was correctly mentioned both in the manuscript and Supporting Information files. There were indeed mistakes in the response to the Reviewer 3.

Reviewer #4

I co-reviewed this manuscript with one of the reviewers who provided the listed reports. This is part of the Nature Communications initiative to facilitate training in peer review and to provide appropriate recognition for Early Career Researchers who co-review manuscripts.

We thank Reviewer #4 and his/her colleague for the thorough reading and comments, which were addressed in the previous revised manuscript.

Other additions:

We realized that we did not add the description of the PFM measurements in the Method Section in the previous revised manuscript. We have now added it.

p.21, line 539: " **Piezoresponse force microscopy.** PFM measurements were performed with a NX10 microscope from Park Systems, in the dual frequency resonance tracking (DFRT) mode (external UHFLI lock-in amplifier from Zurich Instruments). Platinum/iridium coated tips (PPP-EFM, Nanosensors) with a force constant of ~2.8 N/m were used. For the writing/reading experiments, the DC voltage was applied to the tip, and the AC modulation (0.6 V, ~350 kHz) was applied to the chuck."
